# Design and synthesis of novel rigid dibenzo[*b,f*]azepines through ring closure technique as promising anticancer candidates against leukaemia and acting as selective topoisomerase II inhibitors and DNA intercalators

**DOI:** 10.1080/14756366.2022.2157825

**Published:** 2023-01-11

**Authors:** Mohammed Farrag El-Behairy, Walaa Hamada Abd-Allah, Mohamed M. Khalifa, Mohamed S. Nafie, Mohamed A. Saleh, Mohammed S. Abdel-Maksoud, Tarfah Al-Warhi, Wagdy M. Eldehna, Ahmed A. Al‐Karmalawy

**Affiliations:** a Department of Organic and Medicinal Chemistry, Faculty of Pharmacy, University of Sadat City, Menoufiya, Egypt; b Pharmaceutical Chemistry Department, Collage of Pharmaceutical Science and Drug Manufacturing, Misr University for Science and Technology, Giza, Egypt; c Pharmaceutical Medicinal Chemistry and Drug Design Department, Faculty of Pharmacy (Boys), Al-Azhar University, Cairo, Egypt; d Chemistry Department, Faculty of Science, Suez Canal University, Ismailia, Egypt; e Department of Clinical Sciences, College of Medicine, University of Sharjah, Sharjah, The United Arab Emirates; f Department of Pharmacology and Toxicology, Faculty of Pharmacy, Mansoura University, Mansoura, Egypt; g Medicinal and Pharmaceutical Chemistry Department, Pharmaceutical and Drug Industries Research Institute, National Research Centre (ID: 60014618), Giza, Egypt; h Department of Chemistry, College of Science, Princess Nourah bint Abdulrahman University, Riyadh, Saudi Arabia; i Department of Pharmaceutical Chemistry, Faculty of Pharmacy, Kafrelsheikh University, Kafrelsheikh, Egypt; j Pharmaceutical Chemistry Department, Faculty of Pharmacy, Ahram Canadian University, Giza, Egypt

**Keywords:** Dibenzo[bf]azepines, rigidification, ring closure, topoisomerase II, in vivo, SAR

## Abstract

In this research, two novel series of dibenzo[*b,f*]azepines (14 candidates) were designed and synthesised based on the rigidification principle and following the reported doxorubicin’s pharmacophoric features. The anti-proliferative activity was evaluated at the NCI against a panel of 60 cancer cell lines. Further, the promising candidates (**5a–g**) were evaluated for their ability to inhibit topoisomerase II, where **5e** was noticed to be the most active congener. Moreover, its cytotoxicity was evaluated against leukaemia SR cells. Also, **5e** arrested the cell cycle at the G1 phase and increased the apoptosis ratio by 37.34%. Furthermore, *in vivo* studies of **5e** showed the inhibition of tumour proliferation and the decrease in its volume. Histopathology and liver enzymes were examined as well. Besides, molecular docking, physicochemical, and pharmacokinetic properties were carried out. Finally, a SAR study was discussed to open the gate for further optimisation of the most promising candidate (**5e**).

**Highlights**
- Two novel series of dibenzo[*b,f*]azepines were designed and synthesised based on the rigidification principle in drug design.
- The anti-proliferative activity was evaluated at the NCI against a panel of 60 cancer cell lines.
- **5e** was the most active anti-topo II congener (IC_50_ = 6.36 ± 0.36 µM).
- **5e** was evaluated against leukaemia SR cells and its cytotoxic effect was confirmed (IC_50_ = 13.05 ± 0.62 µM).
- *In vivo* studies of **5e** significantly inhibited tumour proliferation by 62.7% and decreased tumour volume to 30.1 mm^3^ compared to doxorubicin treatment.

## Graphical Abstract

**Figure:**
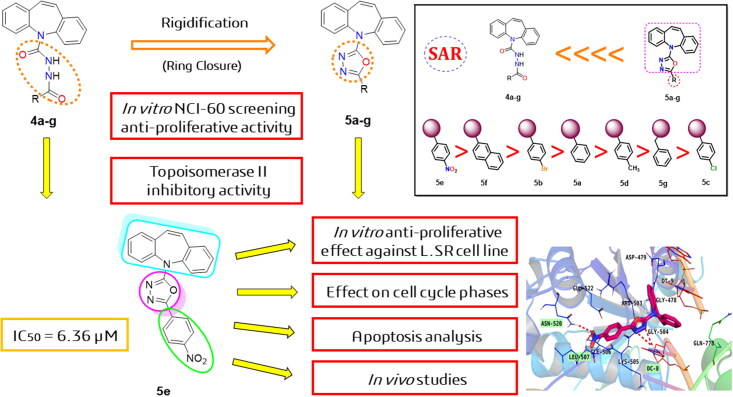

## Introduction

Among the leading causes of death, cancer remains one of the most severe health problems worldwide^1^^,^^2^. It starts when abnormal cells of the body grow uncontrollably in any organ or tissue and then extend outside their normal boundaries to invade adjacent portions of the body and/or spread to more distant organs in metastasis^3^^,^^4^. Globally, the total count of cancer death is one in six deaths (9.6 million) in 2018^5^. However, prostate, lung, stomach, colorectal, and liver cancers are the most common in men, while, breast, colorectal, cervical, and thyroid ones are common among women^6^. The strategy of finding novel chemotherapeutic anticancer agents with improved potency, selectivity, and minimised toxicity is still the target of a large group of researchers^7^^,^^8^. Increasing the understanding of the apoptotic pathway enabled scientists to develop more effective anticancer small molecules.

Multitarget therapies for complicated diseases, including cancer, have gained scientists’ interest over the last few decades^2^^,^^9^^,^^10^. Although cancer treatment faces many challenges, the current chemotherapeutics drugs can effectively prolong patient lives or even completely cure the disease^11^. However, in the hope of maximising their effects, using a combination of more than one drug may be a well-established approach^12–14^. In addition, using a drug that can target two different pathways of cancer development may potentiate the activity and minimise drug resistance^12^. The advantages of the last approach over the former one encouraged us to move towards the design of a multitarget compound.

Among the different chemotherapeutic agent classes still, the DNA-interfering drugs are of great importance either through direct binding to the DNA itself or the enzymes needed for normal DNA functions leading ultimately to cell death^15^^,^^16^. DNA intercalation is a mechanism that gained much attention owing to its hopeful anticancer activity. These agents are distinguished by their ability to intercalate between two base pairs of a DNA molecule, a process that causes structural changes in the DNA molecule that hinders DNA replication^17^. However, A well-defined structural study of several FDA-approved DNA intercalators revealed that the intercalation process is stabilised by the presence of different hydrophobic interactions between the compound’s planner system, chromophore, and the DNA base pairs^18^^,^^19^.

On the reverse side, DNA topoisomerases (topo) are nuclear enzymes that change the topological state of the DNA^20^^,^^21^. DNA replication as well as transcription requires the DNA helix to unwind in a process that causes helical tension in the rest of the DNA molecule^21^^,^^22^. The role of topoisomerase is to free this tension upon the formation of either a single-stranded break (topo I) or a double-stranded break (topoisomerase II) in the DNA double helix^23^. Thus, human topoisomerase II has been considered a promising target for many anticancer agents to control different tumour types.

**Figure 1.**
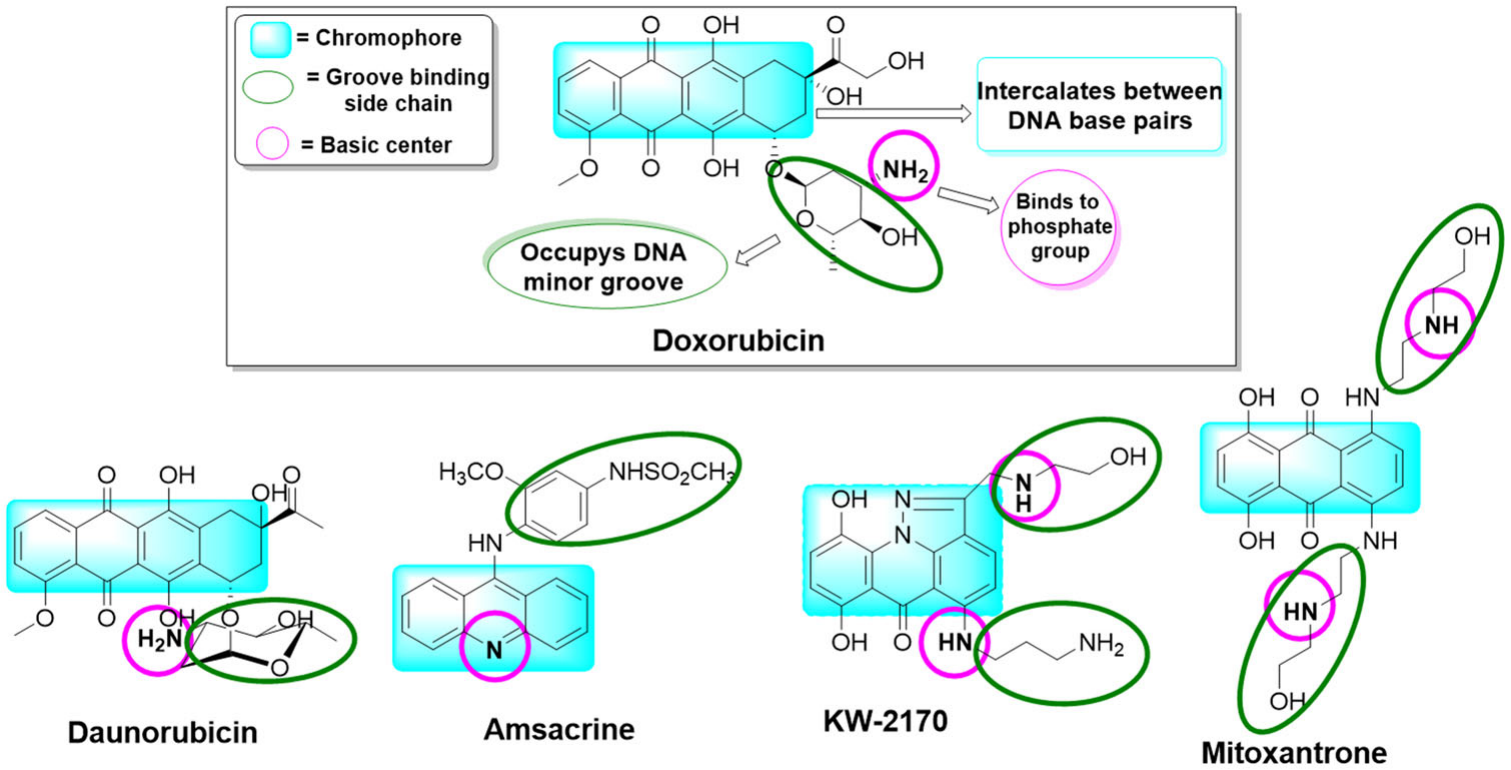
A group of previously reported drugs as topoisomerase II inhibitors and DNA intercalators, bear the same doxorubicin’s pharmacophoric features.

Forced by the former collected data and to continue our previous work^16^^,^^18^^,^^24–26^, we decided to design a multitarget scaffold that may act as both a topoisomerase II inhibitor and DNA intercalator aiming to find new drugs with maximum activity and minimum resistance and side effects. Investigation of drugs that act as DNA intercalators and topoisomerase II inhibitors (Figure 1) showed that the main part that contributes to their action is a planner system that intercalates between the DNA base pairs^27^. In addition, a basic centre linked to the planner system gives the compound the advantage of being protonated at the physiological pH and ionically interacts with the receptor’s phosphate anionic centre^28^^,^^29^.

Deep research in the literature clarified that some heteroaromatic systems bearing a dibenzo[*b,f*]azepine system were proved to be effective topoisomerase II inhibitors and DNA intercalators^30^. In addition, the oxadiazole moiety showed a noticed topoisomerase II inhibitory^31^. Based on these findings, we herein planned to build a new scaffold linking the two mentioned moieties in addition to a groove-binding moiety to obtain potent anticancer candidates.

### The rationale for work design

Our lab teamwork started to design and then synthesise two novel series of dibenzo[*b,f*]azepine derivatives in the hope of finding new promising anticancer agents. This was done based on the reported doxorubicin’s pharmacophoric features, an FDA-approved topoisomerase II inhibitor, and DNA intercalator anticancer drug, Figure 1.

It is worth mentioning that doxorubicin and the other known topoisomerase II inhibitors and DNA intercalators shared three typical features, including, (a) a polyaromatic planner skeleton, chromophore, to be sandwiched between the base pairs of DNA, (b) an easily protonated cationic species to interact with the receptor’s sugar-phosphate moiety and, (c) a side chain to occupy the receptor’s minor groove^16^.

**Figure 2.**
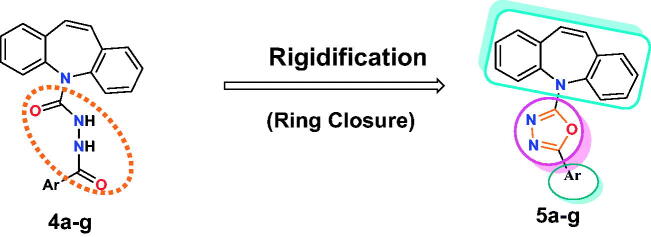
Schematic representation of main pharmacophoric features of the dibenzo[*b,f*]azepine derivatives (**5a–g**) as topoisomerase II inhibitors and DNA intercalators (like doxorubicin) produced by the rigidification of candidates (**4a–g**) using the ring closure principle.

Moreover, guided by the rigidification principle in drug design, which results in increasing the new compounds’ rigidity to improve their binding, increasing their selectivity, and decreasing their side effects^32^. Herein, we synthesised the open analogues of *N*′-benzoyl-5*H*-dibenzo[*b,f*]azepine-5-carbohydrazide candidates (**4a–g**) followed by their chemical conversion into the corresponding closed analogues of 2-(5*H*-dibenzo[*b,f*]azepin-5-yl)-5-phenyl-1,3,4-oxadiazole derivatives (**5a–g**), Figure 2. This was done to study the effect of decreasing the flexibility of compounds (**4a–g**) on the anticancer activity by comparing it to those of the newly designed rigid candidates (**5a–g**).

**Scheme 1.**
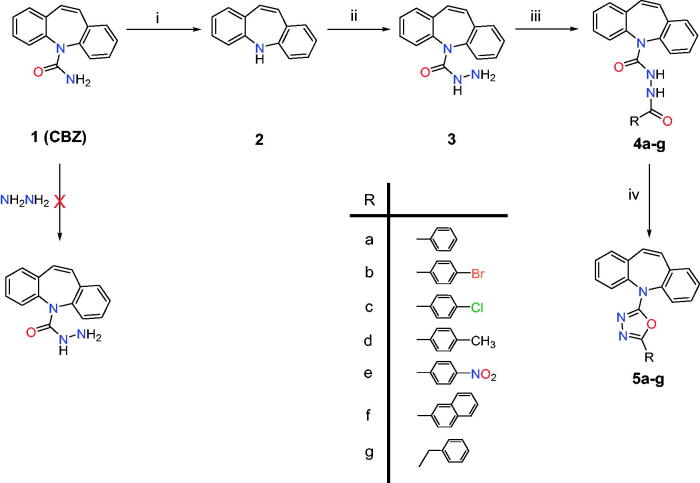
Synthesis of the novel open dibenzo[*b,f*]azepine analogues (**4a–g**) and their corresponding target closed ones (**5a–g**). (i) NaOH/H_2_O/Reflux, (ii) Phosgene/Toluene/NH_2_NH_2_/24 h/RT, (iii) Acid chloride/CHCl_3_/18 h/RT, and (iv) POCl3/30 min/Reflux.

Furthermore, the former criteria of DNA intercalators and topoisomerase II inhibitors were, herein, achieved *via* applying the cross-hybridization strategy. Thus, the dibenzo[*b*,*f*]azepine scaffold was chosen to play the role of the planner polyaromatic system while, an oxadiazole moiety linked to different moieties including, phenyl, 4-bromophenyl, 4-chlorophenyl, *p*-tolyl, 4-nitrophenyl, naphthalenyl, and benzyl were introduced to act as a cationic centre and groove binding moieties, respectively, Figure 2 and Scheme 1.

## Materials and methods

### Chemistry

#### General

The general chemistry protocol was applied as described in the supplementary information (**SI 1**). Moreover, the spectral data of compounds (**4a–g** and **5a–g**), (IR, ^1^H NMR, ^13^C NMR, and Mass spectroscopy) are depicted in the supporting information (**SI 2**).

#### General procedure for the synthesis of 5H-dibenzo[b,f]azepine (2)

Carbamazepine (CBZ, 12 g) was suspended in water (600 ml) then NaOH (32 g, 20 mol equiv.) was added. The mixture was then heated under reflux. The reaction was followed by TLC until no more start then filtered while hot to afford 9.4 g (95%) of Compound **2** as an orange solid. The melting point of Compound **2** was 203 °C–205 °C similar to that recorded in literature^33^.

#### General procedure for the synthesis of 5H-dibenzo[b,f]azepine-5-carbohydrazide (3)

Compound **2** (9.5 g) was suspended in toluene (300 ml) followed by the addition of phosgene (50 ml, 12.5% w/v in toluene) and triethylamine (7.5 ml, 5 g). The mixture was stirred at room temperature for 24 h. Then, excess hydrazine hydrate (100 ml) was added to the mixture and stirred for 1 h then cooled and the product (6.0 g, 48%) was filtered off to produce a white solid of Compound **3** with a melting point of 192 °C–194 °C^34^.

#### *General procedure for the synthesis of N*′*-benzoyl-5H-dibenzo[b,f]azepine-5-carbohydrazide derivatives (4a–g)*

To a stirred solution of compound **3** (0.75 g, 3 mmol) in chloroform, appropriate acid chloride (3 mmol) was added followed by triethylamine (0.3 g, 0.42 ml, 3 mmol). The reaction mixture was stirred at room temperature for 18 h and then washed with NaHCO_3_ (10%, 20 ml) and water. The organic layer was then separated, dried with Na_2_SO_4_, and evaporated under vacuum to afford crude products **4a–g,** which were further purified by crystallisation from ethanol.

##### *N*′-Benzoyl-5*H*-dibenzo[*b*,*f*]azepine-5-carbohydrazide (4a)

Yield (0.9 g, 90%), white solid, m.p. 222 °C–224 °C. ^1^H NMR (400 MHz, DMSO-D6) *δ* 10.08 (s, 1H), 7.82–7.76 (m, 2H), 7.73 (s, 1H), 7.53–7.38 (m, 9H), 7.33 (ddd, *J* = 8.2, 5.8, 2.6 Hz, 2H), 7.00 (s, 2H); ^13^C NMR (101 MHz, DMSO-D6) *δ* 166.36, 155.92, 140.06, 135.29, 133.26, 132.11, 130.91, 129.99, 129.77, 129.42, 129.09, 128.89, 127.98, 127.84; ESI^–^-MS (m/z, %): 354 ([M-H]^–^, 100%); 355 ([M]^–^, 28%).

##### *N*′-(4-Bromobenzoyl)-5*H*-dibenzo[*b*,*f*]azepine-5-carbohydrazide (4b)

Yield (0.86 g, 66%), white solid, m.p. 237 °C–239 °C. IR (KBr, cm^−1^): 3375 (NH), 3259 (NH), 1658 (broad, C = O); ^1^H NMR (400 MHz, DMSO-D6) *δ* 10.16 (s, 1H), 7.77 (s, 1H), 7.72 (d, *J* = 8.6 Hz, 2H), 7.64 (d, *J* = 8.6 Hz, 2H), 7.49–7.39 (m, 6H), 7.33 (ddd, *J* = 8.2, 5.4, 3.1 Hz, 2H), 6.99 (s, 2H); ^13^C NMR (101 MHz, DMSO-D6) *δ* 165.50, 155.84, 139.99, 135.26, 132.00, 130.89, 129.93, 129.76, 129.40, 128.00, 125.94; ESI-MS (m/z, %): 432 (^79^Br), ([M-H]^–^, 100%) 433 (^79^Br) ([M]^–^, 26%) , 434 (^81^Br) ([M-H]^–^, 100%), 435 (^81^Br) ([M]^–^, 26%).

##### *N*′-(4-Chlorobenzoyl)-5*H*-dibenzo[*b*,*f*]azepine-5-carbohydrazide (4c)

Yield (0.8 g, 70%), white solid, m.p. 229 °C–231 °C. ^1^H NMR (400 MHz, DMSO-D6) *δ* 10.16 (s, 1H), 8.28 (s, 1H), 7.79 (t, *J* = 7.7 Hz, 2H), 7.50 (d, *J* = 8.6 Hz, 2H), 7.47–7.39 (m, 6H), 7.33 (ddd, *J* = 8.2, 5.5, 2.9 Hz, 2H), 6.99 (s, 2H); ^13^C NMR (101 MHz, DMSO-D6) *δ* 165.37, 155.86, 140.01, 136.99, 135.27, 132.01, 130.89, 129.99, 129.76, 129.40, 129.06, 128.00, 79.72; ESI-MS (m/z, %): 388 ([M-H]^–^, 100%); 389 ([M]^–^, 30%), 390 ([M + H]^–^, 34%).

##### *N*′-(4-Methylbenzoyl)-5*H*-dibenzo[*b*,*f*]azepine-5-carbohydrazide (4d)

Yield (0.62 g, 60%), white solid, m.p. 151 °C–153 °C. ^1^H NMR (400 MHz, DMSO-D6) *δ* 10.00 (s, 1H), 7.69 (d, *J* = 8.2 Hz, 3H), 7.44 (ddd, *J* = 9.0, 5.1, 0.9 Hz, 6H), 7.36–7.29 (m, 2H), 7.22 (d, *J* = 8.0 Hz, 2H), 6.99 (s, 2H), 2.29 (s, 3H). ^13^C NMR (101 MHz, DMSO-D6) *δ* 166.23, 155.95, 142.04, 140.06, 135.28, 130.90, 130.44, 129.98, 129.75, 129.41, 127.97, 127.86, 21.50. ESI-MS (m/z, %): 368 ([M-H]^–^, 100%); 369 ([M]^–^, 30%).

##### *N*′-(4-Nitrobenzoyl)-5*H*-dibenzo[*b*,*f*]azepine-5-carbohydrazide (4e)

Yield (0.9 g, 81%), yellow solid, m.p. 241 °C–243 °C. IR (KBr, cm^−1^): 3402 (NH), 3251 (NH), 1697 (C = O), 1658 (C = O); ^1^H NMR (400 MHz, DMSO-D6) *δ* 10.40 (s, 1H), 8.27 (d, *J* = 9.0 Hz, 2H), 7.99 (d, *J* = 9.0 Hz, 2H), 7.90 (s, 1H), 7.49–7.40 (m, 6H), 7.38–7.29 (m, 2H), 7.00 (s, 2H); ^13^C NMR (101 MHz, DMSO-D6) *δ* 164.87, 155.74, 149.77, 141.98, 139.95, 138.96, 135.27, 130.90, 130.02, 129.78, 129.40, 129.24, 128.04, 124.20; ESI-MS (m/z, %): 399 ([M-H]^–^, 100%); 400 ([M]^–^, 30%), 401 ([M + H]^–^, 10%).

##### *N*′-(2-Naphthoyl)-5*H*-dibenzo[*b*,*f*]azepine-5-carbohydrazide (4f)

Yield (0.82 g, 80%), white solid, m.p. 243 °C–245 °C. ^1^H NMR (400 MHz, DMSO-D6) *δ* 9.99 (s, 1H), 8.36–8.24 (m, 1H), 8.00–7.86 (m, 3H), 7.60–7.41 (m, 10H), 7.40–7.30 (m, 2H), 7.02 (s, 2H); ^13^C NMR (101 MHz, DMSO-D6) *δ* 168.59, 155.91, 140.08, 135.34, 133.77, 133.53, 130.95, 130.49, 130.47, 130.04, 129.80, 129.52, 128.56, 128.02, 127.13, 126.84, 126.23, 125.81, 125.47; ESI-MS (m/z, %): 404 ([M-H]^–^, 40%); 405 ([M]^–^, 26%).

##### *N*′-(2-Phenylacetyl)-5*H*-dibenzo[*b*,*f*]azepine-5-carbohydrazide (4g)

Yield (0.35 g, 30%), white solid, m.p. 134 °C–136 °C. IR (KBr, cm^−1^): 3379 (NH), 3290 (NH), 1666 (broad, C = O); ^1^H NMR (400 MHz, DMSO-D6) *δ* 9.73 (d, *J* = 1.5 Hz, 1H), 7.61 (d, *J* = 1.5 Hz, 1H), 7.40 (ddd, *J* = 4.8, 3.6, 1.1 Hz, 6H), 7.34–7.27 (m, 2H), 7.22 (d, *J* = 4.4 Hz, 4H), 7.15 (dd, *J* = 8.1, 5.3 Hz, 1H), 6.96 (s, 2H), 3.34 (s, 2H); ^13^C NMR (101 MHz, DMSO-D6) *δ* 169.82, 155.68, 140.02, 136.30, 135.26, 130.85, 129.94, 129.72, 129.60, 129.39, 128.61, 127.94, 126.87, 45.82; ESI-MS (m/z, %): 368 ([M-H]^–^, 100%); 369 ([M]^–^, 26%).

#### General procedure for synthesis of 2–(5H-dibenzo[b,f]azepin-5-yl)-5-phenyl-1,3,4-oxadiazole derivatives 5a–g

A solution of an appropriate derivative **4a**–**g** in phosphorusoxychloride (POCl_3_, 5.0 ml) was refluxed for 30 min. The solution was then diluted carefully with water then NaHCO_3_ was added till being alkaline. The precipitate was filtered, washed with water, and recrystallized from ethanol to afford compounds **5a–g**.

##### 2-(5 *h*-Dibenzo[*b*,*f*]azepin-5-yl)-5-phenyl-1,3,4-oxadiazole (5a)

Yield (0.25 g, 65%), white solid, m.p. 209 °C–211 °C, ^1^H NMR (400 MHz, CHLOROFORM-D) *δ* 7.77–7.71 (m, 2H), 7.60 (d, *J* = 7.9 Hz, 2H), 7.53–7.45 (m, 2H), 7.37 (t, *J* = 6.1 Hz, 7H), 6.91 (s, 2H); ^13^C NMR (101 MHz, CDCl_3_) *δ* 162.82, 159.38, 140.04, 134.60, 130.73, 130.58, 129.82, 129.77, 129.51, 129.11, 128.72, 128.22, 128.12, 127.87, 125.91, 124.31; APCI^+^-MS (m/z, %) 338 ([M + H]^+^ 100%).

##### 2-(4-Bromophenyl)-5-(5*H*-dibenzo[*b*,*f*]azepin-5-yl)-1,3,4-oxadiazole (5b)

Yield (0.3 g, 70%), white solid, m.p. 185 °C–187 °C. ^1^H NMR (400 MHz, CHLOROFORM-D) *δ* 7.61–7.56 (m, 4H), 7.52–7.45 (m, 4H), 7.38 (dd, *J* = 5.9, 1.9 Hz, 4H), 6.91 (s, 2H); ^13^C NMR (101 MHz, CDCl_3_) *δ* 162.96, 158.66, 139.91, 134.57, 132.01, 130.71, 129.85, 129.79, 128.18, 127.81, 127.30, 124.92, 123.27; APCI^+^-MS (m/z,%): 416 (^79^Br) ([M]^+^ 100%), 417 (^79^Br) ([M + H]^+^ 28%), 418 (^81^Br) ([M]^+^ 100%), 419 (^81^Br) ([M + H]^+^ 100%).

##### 2-(4-Chlorophenyl)-5-(5*H*-dibenzo[*b*,*f*]azepin-5-yl)-1,3,4-oxadiazole (5c)

Yield (0.26 g, 68%), white solid, m.p. 174 °C–176 °C. ^1^H NMR (400 MHz, CHLOROFORM-D) *δ* 7.64 (s, 2H), 7.56 (s, 2H), 7.47 (d, *J* = 3.6 Hz, 2H), 7.42–7.25 (m, 6H), 7.00–6.82 (m, 2H); ^13^C NMR (101 MHz, CDCl_3_) *δ* 162.94, 158.58, 139.92, 136.59, 134.57, 130.71, 129.85, 129.80, 129.07, 128.18, 127.81, 127.14, 124.15, 122.82; APCI^+^-MS (m/z, %): 372 ([M + H]^+^ 100%).

##### 2-(5 *h*-Dibenzo[*b*,*f*]azepin-5-yl)-5-(p-tolyl)-1,3,4-oxadiazole (5d)

Yield (0.22 g, 73%), white solid, m.p. 239 °C–241 °C. ^1^H NMR (400 MHz, CHLOROFORM-D) *δ* 7.61 (dd, *J* = 12.9, 8.1 Hz, 4H), 7.52–7.44 (m, 2H), 7.37 (d, *J* = 4.1 Hz, 4H), 7.15 (d, *J* = 8.0 Hz, 2H), 6.90 (s, 2H), 2.34 (s, 3H); ^13^C NMR (101 MHz, CDCl_3_) *δ* 162.68, 159.58, 140.89, 140.18, 134.61, 130.75, 129.79, 129.73, 129.40, 128.03, 127.88, 125.89, 121.60, 21.55; APCI^+^-MS (m/z, %): 352 ([M + H]^+^ 100%).

##### 2-(5 *h*-Dibenzo[*b*,*f*]azepin-5-yl)-5–(4-nitrophenyl)-1,3,4-oxadiazole (5e)

Yield (0.39 g, 92%), green solid, m.p. 146 °C–148 °C. IR (KBr, cm^−1^): 1604 (C = N), 1573 (C = N). ^1^H NMR (400 MHz, CHLOROFORM-D) *δ* 8.24–8.22 (m, 1H), 8.22–8.20 (m, 1H), 7.91–7.89 (m, 1H), 7.89–7.87 (m, 1H), 7.60 (s, 1H), 7.58 (s, 1H), 7.54–7.47 (m, 2H), 7.41 (dd, *J* = 1.9, 0.8 Hz, 2H), 7.40 (d, *J* = 0.9 Hz, 2H), 6.92 (s, 2H); ^13^C NMR (101 MHz, CDCl_3_) *δ* 163.52, 157.68, 148.63, 139.49, 134.50, 130.68, 129.95, 129.91, 129.77, 128.44, 127.70, 126.48, 124.15; APCI^+^-MS (m/z, %): 383 ([M + H]^+^ 100%).

##### 2-(5 *h*-Dibenzo[*b*,*f*]azepin-5-yl)-5-(naphthalen-2-yl)-1,3,4-oxadiazole (5f)

Yield (0.27 g, 68%), white solid, m.p. 201 °C–203 °C. ^1^H NMR (400 MHz, CHLOROFORM-D) *δ* 9.15 (d, *J* = 8.7 Hz, 1H), 7.91–7.81 (m, 2H), 7.75 (dd, *J* = 7.3, 1.2 Hz, 1H), 7.66 (d, *J* = 7.9 Hz, 2H), 7.60–7.46 (m, 4H), 7.39 (d, *J* = 4.3 Hz, 5H), 6.93 (s, 2H); ^13^C NMR (101 MHz, CDCl_3_) *δ* 162.61, 159.39, 140.13, 134.64, 133.75, 131.44, 130.78, 129.86, 129.78, 128.46, 128.13, 127.92, 127.76, 126.93, 126.47, 124.73, 120.76; APCI^+^-MS (m/z, %): 388 ([M + H]^+^ 100%).

##### 2-Benzyl-5-(5*H*-dibenzo[*b*,*f*]azepin-5-yl)-1,3,4-oxadiazole (5g)

Yield (0.2 g, 60%), white solid, m.p. 189 °C–191 °C. ^1^H NMR (400 MHz, CHLOROFORM-D) *δ* 7.46 (d, *J* = 7.9 Hz, 2H), 7.44–7.38 (m, 2H), 7.33 (dd, *J* = 4.6, 1.0 Hz, 4H), 7.26 (ddd, *J* = 6.7, 5.3, 2.6 Hz, 3H), 7.19 (dd, *J* = 7.8, 1.6 Hz, 2H), 6.87 (s, 2H), 3.96 (s, 2H); ^13^C NMR (101 MHz, CDCl_3_) *δ* 163.25, 159.55, 140.13, 134.55, 134.41, 130.70, 129.73, 129.66, 128.70, 128.66, 127.97, 127.81, 127.20, 31.78; APCI^+^-MS (m/z, %): 352 ([M + H]^+^ 100%).

### Biological activity

#### In vitro NCI-60 one-dose screening anti-proliferative activity

Anti-proliferative effects of the counterparts **4a–g** and **5a–g** were screened against 60 human cancer cell lines, following the Developmental Therapeutics Program protocol, National Cancer Institute^35^.

#### Topoisomerase II inhibitory assay

All the promising anticancer closed analogues (**5a–g**) were then investigated to measure the topoisomerase II inhibitory effect using the reported procedure by Patra *et al.*^36^ and doxorubicin as a reference drug.

#### Anti-proliferative effect against SR cell line

The anti-proliferative effect of the **5e** counterpart against the SR cell line was estimated using the MTT assay protocol^37^.

#### Effect on cell cycle phases

This test was performed using propidium iodide (PI) staining following the detailed procedure in the supporting information (**SI 3**)^38^.

#### Apoptosis analysis

The apoptotic analysis was performed according to the detailed description in the supporting information (**SI 4**)^39^.

#### In vivo studies

The full methodology of tumour induction, treatments, and blood measurements was carried out according to the described details in the supporting information (**SI 5**). Moreover, the experimental protocol was approved by the Research Ethics Committee at Suez Canal University (Approval number REC139/2022, Chemistry Department, Faculty of Science, Suez Canal University).

### In silico studies

#### Molecular modelling

Discovery studio software 4.1^40^ was used to carry out molecular docking studies at the Misr University for Science and Technology. The selected poses of the most promising candidates were visualised using PyMOL program software^41^. This study was achieved to examine the binding interactions of the target entities against topoisomerase II complexed with DNA by using **EVP** and doxorubicin as reference standards.

##### The tested candidates’ preparation

The ChemDraw Professional 16.0 was used to sketch the target compounds. Then, the target compounds, doxorubicin, and **EVP** were inserted into a single database and exposed to force field energy minimisation^42^^,^^43^.

##### Topoisomerase II-DNA complex preparation

The X-ray structure of human topoisomerase II complexed with DNA was extracted from the protein data bank (ID: 3QX3). The downloaded complex was prepared by the deletion of water residues followed by 3 D protonation and fixation of the missed amino acids^44^.

##### Docking into topoisomerase II-DNA target

Docking procedures were achieved and the proper binding pose was selected. This was carried out using Discovery studio software 4.1 protocols^40^. The docking poses were accomplished according to their binding energies. Briefly, the best binding pose was selected according to some criteria like having the crucial previously mentioned binding amino acids.

#### Physicochemical and pharmacokinetic properties

The physicochemical and pharmacokinetic parameters were evaluated using SwissADME (http://swissadme.ch/index.php), a free online web tool^45^^,^^46^.

## Results and discussion

### Chemistry

Iminostilbene **2** was synthesised with a very good yield (95%) from CBZ, for the first time, *via* basic hydrolysis using aqueous NaOH. Then, carbohydrazide **3** was prepared following the reported procedures^34^^,^^47^. Indeed, carbohydrazide **3** could be prepared from CBZ or compound **2** *via* diverse chemical pathways. Hydrazinolysis of CBZ would provide compound **3** in one step; however, it did not work. Moreover, the reaction of **2** with alkylchloroformates followed by hydrazinolysis led to compound **3**. Also, the reaction of **2** with phosgene followed by the addition of hydrazine to the produced carbonyl chloride will afford compound **3** in a “two-step one-pot” reaction instead of two isolated reactions in the former method (Scheme 1). The latter method has been adopted to prepare compound **3** with a good yield. Further reaction of **3** with different acid chlorides at room temperature in chloroform and the presence of triethylamine afforded compounds (**4a–g**).

Compounds **4a–g** have been characterised by NMR and Mass spectroscopy as described in the experimental section and supporting information. In ^13^C-NMR, two signals corresponding to carbonyl carbon have been observed at *δ*_C_ 164–169 and *δ*c 153–157 ppm. Also, all aromatic carbons were observed at *δ*_C_ 100–150 ppm, and the aliphatic methyl of **4d** and methylene of **4g** were detected at *δ*_C_ 21.5 and 45 ppm, respectively. The ^1^H-NMR showed only aromatic protons for all compounds except compound **4d**, which displayed methyl signal at *δ*_H_ 2.29 ppm, and compound **4g,** where a very characteristic triplet resulted from overlapped doublet of doublet signals of the prochiral methylene group was noted at *δ*_H_ 1.15 ppm. The most characteristic signal of compounds **4a–g** is the amidic NH singlet signal at *δ*_H_ 10.0 ppm, while the other amidic NH was observed at *δ*_H_ 7.0–8.0 ppm, very close to aromatic protons. The mass spectra of compounds **4a–g** were performed using negative mode ESI and showed the molecular ion signal as a minor peak, but the [M-H]^–^ was present in all spectra as the base peak.

Compounds **5a–g** (1,3,4-oxadiazole derivatives) have been accomplished through intramolecular cyclodehydration of compounds **4a–g**. Heating in solvents like pyridine, or DMF is a method of Cyclodehydration. Moreover, cyclisation is performed in presence of catalysts such as SOCl_2_, P_2_O_5_, 1-ethyl-3–(3-dimethylaminopropyl)carbodiimide (EDC), POCl_3_, H_2_SO_4_, triphenylphospine, Burgess reagent, or triflic anhydride^48^. In this study, POCl_3_ was used as a solvent and cyclo-dehydrating agent. Besides, the addition of water to the hot reaction (very slowly) to hydrolyse POCl_3_ was a very important step in getting filterable powder.

Spectral confirmation of compounds **5a–g** has been performed where the NH bands have disappeared from ^1^H-NMR. In ^13^C-NMR two imine signals of the oxadiazole ring were revealed at *δ* 157–164 ppm. The mass spectra of compounds **5a–g** have been performed in positive APCI and showed the molecular ion peak as the base peak for all compounds.

### Biological activity

#### In vitro NCI-60 one-dose screening anti-proliferative activity

The anti-tumour activity of the fourteen synthesised congeners was investigated at the NCI against a panel of 60 cancer cell lines (NCI-60 Cell One-Dose Screen). The tested dibenzo[*b*,*f*]azepine derivatives were screened at a single dose level (10 μM), Table 1.

**Table 1.** Growth inhibition % of the tested compounds (**4a–g** and **5a–g**) against different 60 cell lines (NCI-60).

Subpanel tumour cell lines	Compounds													
	4a	4b	4c	4d	4e	4f	4g	5a	5b	5c	5d	5e	5f	5g
Leukaemia														
CCRF-CEM	–	–	–	–	–	–	–	26.09	37.32	30.43	26.62	–	28.17	15.64
HL-60(TB)	–	–	–	–	–	10.04	–	13.19	34.63	38.12	33.79	30.06	–	10.63
K-562	–	–	–	–	–	12.83	–	32.25	54.54	53.99	47.81	25.26	33.98	14.86
MOLT-4	–	–	–	–	–	–	–	16.54	35.64	20.40	20.98	19.35	16.32	10.86
RPMI-8226	–	–	–	–	–	–	–	67.73	17.48	19.19	52.00	–	12.08	27.84
SR	–	–	–	–	–	12.77	–	–	72.97	67.50	60.59	30.12	58.44	17.55
Non-small cell lung cancer														
A549-ATCC	–	18.50	–	–	–	–	–	–	29.84	20.34	12.45	16.51	28.19	24.67
EKVX	–	–	–	–	–	11.01	13.76	–	27.55	20.83	–	–	28.86	2.24
HOP-62	–	–	–	–	–	–	–	15.34	22.73	15.39	–	–	–	18.40
HOP-92	–	–	–	–	–	18.58	–	18.28	33.48	23.99	14.33	22.82	20.92	–
NCI-H226	–	–	–	–	–	–	–	–	28.26	27.52	21.65	13.72	21.42	–
NCI-H23	–	–	–	–	–	–	–	–	–	–	–	–	12.36	–
NCI-H322M	–	–	–	–	–	–	–	50.19	–	10.54	–	4.97	9.52	–
NCI-H460	–	–	–	–	–	–	–	–	63.47	57.26	58.60	1.84	25.09	17.45
NCI-H522	–	–	–	–	–	14.06	–	–	26.33	22.24	17.10	15.03	24.20	–
Colon cancer														
COLO 205	–	–	–	–	–	–	–	–	15.40	–	19.42	–	–	–
HCC-2998	–	–	–	–	–	–	–	33.39	–	–	–	–	–	–
HCT-116	–	–	–	–	–	–	–	54.24	52.18	47.74	45.78	–	27.26	31.40
HCT-15	–	–	–	–	–	–	–	–	66.47	63.52	61.14	30.73	57.04	4.03
HT29	–	–	–	–	–	11.44	–	–	18.42	–	–	–	–	–
KM12	–	–	–	–	–	–	–	–	21.42	18.46	13.08	–	–	–
SW-620	–	11.82	–	–	–	–	–	–	–	–	–	–	–	19.70
CNS cancer														
SF-268	–	15.10	–	–	–	–	–	–	24.68	24.70	10.10	16.05	21.94	10.68
SF-295	–	–	–	–	–	–	–	–	30.39	29.55	22.01	–	19.93	–
SF-539	–	–	–	–	–	–	–	–	17.17	–	–	–	–	11.46
SNB-19	–	–	–	–	–	–	–	33.22	15.56	11.15	–	–	–	–
U251	–	–	–	–	–	–	–	20.15	47.02	42.56	31.80	––	32.33	10.12
Melanoma														
LOX IMVI	–	–	–	–	–	–	–	–	38.76	35.74	25.60	–	18.73	–
MALME-3M	–	–	–	–	–	–	–	–	–	–	–	–	–	–
M14	–	–	–	–	–	–	–	12.06	–	–	–	–	–	–
MDA-MB-435	–	–	–	–	–	–	–	–	–	–	–	–	–	–
SK-MEL-2	–	–	–	–	–	–	–	–	–	–	–	–	–	–
SK-MEL-28	–	–	–	–	–	–	–	28.02	14.13	–	–	–	–	–
SK-MEL-5	–	–	–	–	–	–	–	–	53.17	47.97	34.61	13.66	15.84	–
UACC-257	–	14.38	12.55	–	–	–	–	13.62	–	–	–	–	–	19.15
UACC-62	–	–	–	–	–	18.93	–	–	32.21	31.29	20.91	29.97	12.77	10.82
Ovarian cancer														
IGROV1	–	–	–	–	–	–	–	–	21.55	19.19	–	–	29.07	–
OVCAR-3	–	–	–	–	–	–	–	–	–	–	–	–	2.22	12.33
OVCAR-4	–	–	–	–	–	–	–	–	19.60	22.10	–	21.82	–	–
OVCAR-5	–	–	–	–	–	–	–	–	–	–	–	–	–	–
OVCAR-8	–	–	–	–	–	–	–	–	28.56	24.00	11.53	1.15	9.31	16.50
NCI/ADR-RES	–	–	–	–	–	–	–	–	23.41	14.54	–	–	12.05	–
SK-OV-3	–	–	–	–	–	–	–	–	–	–	–	–	–	–
Renal cancer														
786-0	–	21.12	–	–	–	–	–	19.99	11.30	–	–	–	–	18.78
A498	–	–	–	–	–	–	–	–	13.39	12.95	–	14.79	–	–
ACHN	–	13.98	13.31	–	–	–	–	16.72	24.77	15.52	–	–	10.51	27.70
CAKI-1	–	–	–	–	–	–	–	–	31.88	28.47	16.19	29.70	33.13	–
RXF 393	–	–	–	–	–	–	–	–	24.94	17.92	–	–	10.44	–
SN12C	–	–	–	–	–	–	–	–	24.19	21.89	–	–	15.71	–
TK-10	–	–	13.25	10.32	10.01	–	–	16.05	–	–	–	–	20.26	21.15
UO-31	–	–	–	–	–	12.04	–	39.35	33.49	33.76	–	30.72	24.71	30.67
Prostate cancer														
PC-3	–	–	–	–	–	25.11	–	–	48.54	44.08	41.40	24.58	40.89	–
DU-145	–	19.68	–	–	–	–	–	12.42	–	–	–	–	–	16.34
Breast cancer														
MCF-7	–	–	–	–	–	13.97	–	14.45	30.47	35.65	15.73	17.44	24.40	–
MDA-MB-231-ATCC	–	–	–	–	–	–	–	–	22.27	19.78	9.21	15.31	–	–
HS 578 T	–	–	–	–	–	–	–	–	14.36	–	13.16	–	–	24.07
T-47D	–	–	–	–	–	12.61	–	–	21.46	–	–	18.42	18.29	12.00
MDA-MB-468	–	–	–	–	–	12.30	–	26.09	17.77	16.69	–	11.44	11.13	15.64

(–) Growth inhibition % for samples that is not active.

Notably, the open analogues of dibenzo[*b,f*]azepine carbohydrazide derivatives (**4a–g**) showed very weak anticancer activities towards the tested 60 cancer cell lines. However, the corresponding closed analogues of dibenzo[*b,f*]azepin oxadiazole derivatives (**5a–g**) achieved remarkable anticancer activities.

Collectively, the most responsive cell line among the tested cells was leukaemia cancer cell lines, in particular, the SR cell line. The growth inhibition GI % of the tested congeners **5b, 5c, 5d, 5e, 5f,** and **5 g** against SR cells were 72.97%, 67.50%, 60.59%, 30.12%, 58.44%, and 17.55%, respectively.

#### Topoisomerase II inhibitory activity

Aiming to confirm the suggested mechanism, the synthesised closed analogues of dibenzo[*b,f*]azepin oxadiazole congeners (**5a–g**) with the highest anti-tumour activities were all examined for their ability to inhibit topoisomerase II *in vitro*. Therefore, following the procedure reported by Patra *et al*., the topoisomerase II activity was evaluated^36^. Doxorubicin, an FDA-approved topoisomerase II inhibitor, was co-assayed as a reference. The IC_50_ values of the tested congeners and doxorubicin were recorded and represented in Table 2.

**Table 2.** Topoisomerase II activity of the tested candidates (**5a–g**) against doxorubicin.

Ser	Candidate	Topoisomerase II (IC_50_) µM	SD±
1	**5a**	15.63	0.86
2	**5b**	10.24	0.58
3	**5c**	31.76	1.78
4	**5d**	16.83	0.94
5	**5e**	6.362	0.36
6	**5f**	9.561	0.53
7	**5g**	18.95	1.06
–	Doxorubicin	3.445	0.19

An initial overview revealed that the synthesised members possessed potent to moderate topoisomerase II inhibiting activities with IC_50_ ranging from 6.36 ± 0.36 to 31.76 ± 1.78 µM. Obviously, compound **5e** was noticed to be the most active among the tested new members, as it inhibited topoisomerase II in an IC_50_ value of 6.36 ± 0.36 µM which was quite close to that of doxorubicin (IC_50_ = 3.44 ± 0.19 µM). Furthermore, **5b** and **5f** members exhibited efficient topoisomerase II inhibitory activities with IC_50_ of 10.24 ± 0.58 and 9.561 ± 0.53 µM, respectively, Table 2.

**Figure 3.**
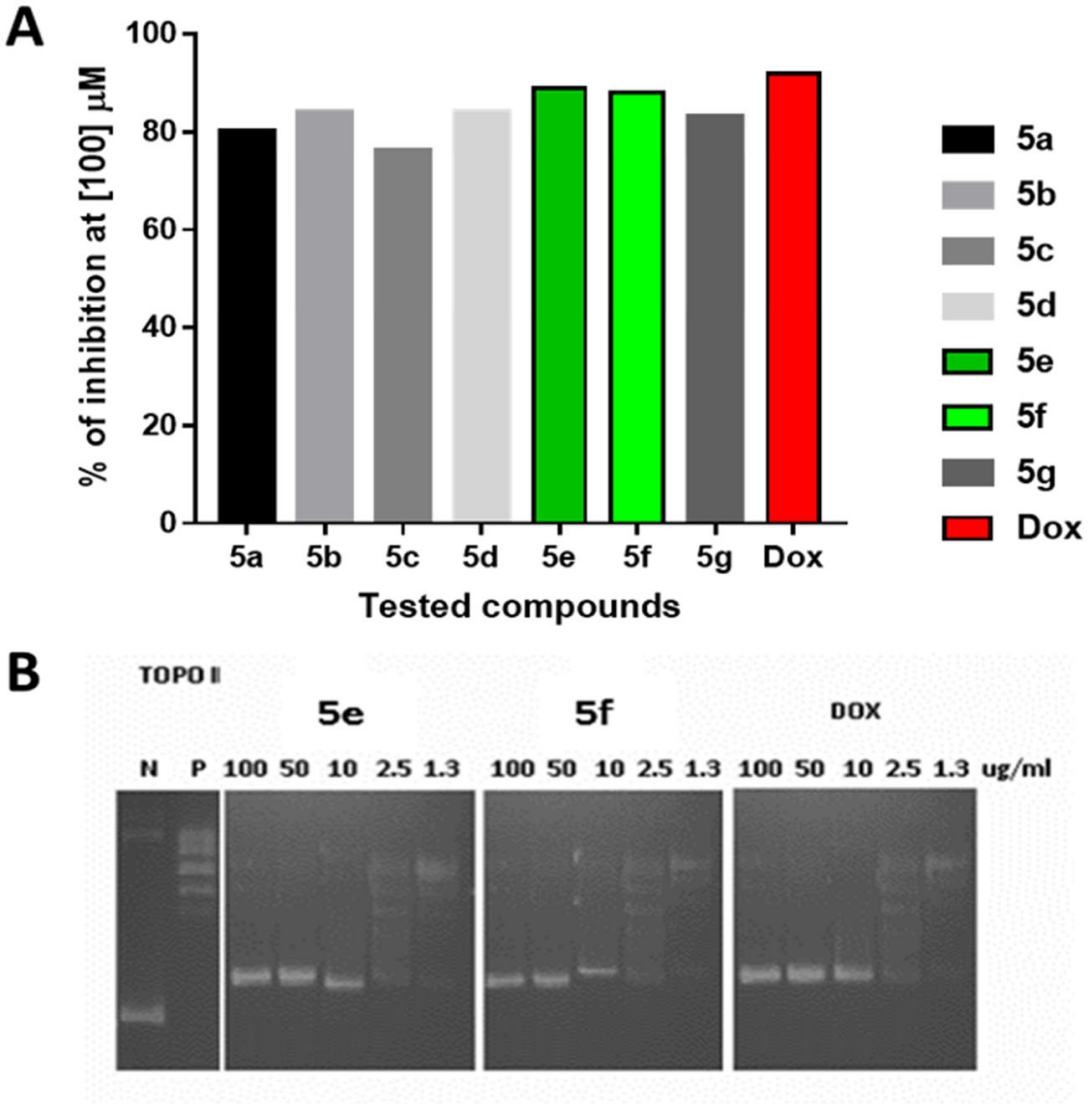
Topoisomerase II inhibitory activity of the tested compounds (**5a–g**) against doxorubicin. (A) Percentage of topoisomerase II inhibition at concentration (100 µM) of the tested compounds; (B) DNA-fragmentation gel images of the most active compounds (**5e** and **5f**) at various concentrations (100–1.3 µg/ml).

As seen in Figure 3, compounds **5e** and **5f** with the promising IC_50_ values exhibited the largest cell inhibition of 89, and 88% inhibition at the concentration of 100 µM.

#### Anti-proliferative effect against SR cancer cell line

Depending on the previous results, the cytotoxic activity of the designed candidate **5e** was then evaluated against leukaemia SR cells. Doxorubicin was also evaluated as a reference drug. The obtained results confirmed the cytotoxic effect of the examined member **5e** with an IC_50_ value of 13.05 ± 0.62 µM against the aforementioned cell line.

Following, the cytotoxic effect of compound **5e** against a normal cell line, PSC-800–011, was also assigned to evaluate the selectivity of the compound towards the cancer cell lines. Results revealed the weak effect of compound **5e** against the normal cell line. However, the selectivity index (SI) of compound **5e** was also calculated to confirm its selectivity. It was known that any sample that has an SI value higher than 3 will be considered to have high selectivity. Thus, the results demonstrated in Table 3 revealed the high selectivity of the tested member.

**Table 3.** The cytotoxicity results of compound **5e** against SR and normal cell lines.

Compound	Cytotoxicity IC_50_ µg/ml		SI
	SR	PSC-800-011	
**5e**	13.05 ± 0.62	43.86 ± 2.68	3.36
Doxorubicin	3.78 ± 0.14	12.83 ± 0.79	3.39

#### Effect on cell cycle phases

**Figure 4.**
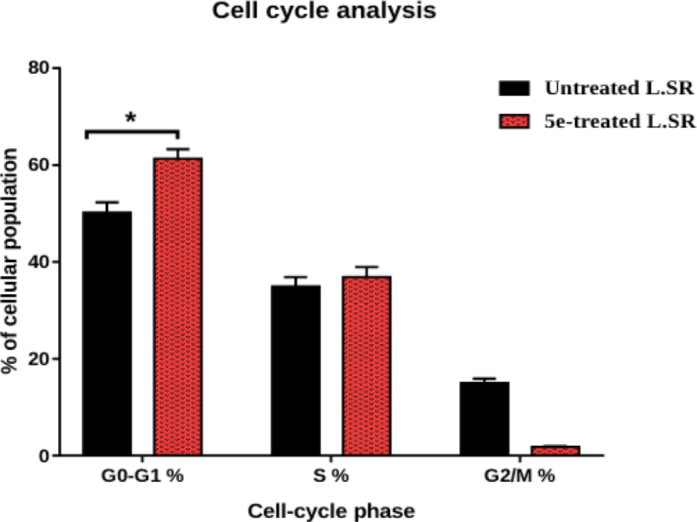
Analysis of cell cycle for **5e**-treated SR cells. *P < 0.05 compared to control by GraphPad prism using unpaired *t*-test.

The topoisomerase II inhibitory evaluation, besides the cytotoxicity results of the new compounds, encouraged us to assess the effect of **5e** (the most promising candidate) on cell cycle progression in SR cells. Notably, the **5e** effects on the cell cycle, besides the %age of the cellular population in each phase are summarised in Figure 4.

Observing the obtained results revealed that member **5e** arrested cell cycle progression at the G1 phase as it caused a significant increase of the cell levels to 61.29% versus 52.84% accumulation of the control cells. It was also disclosed that member **5e** caused an increase in the S phase % of the examined cells to 36.86% instead of 34.18% in the untreated cells. The former results confirmed the potential of **5e** to arrest SR cell progression at the G1 phase effectively. Cell cycle histograms are supported in the supporting information (Figure SI 1).

#### Apoptosis analysis

**Figure 5.**
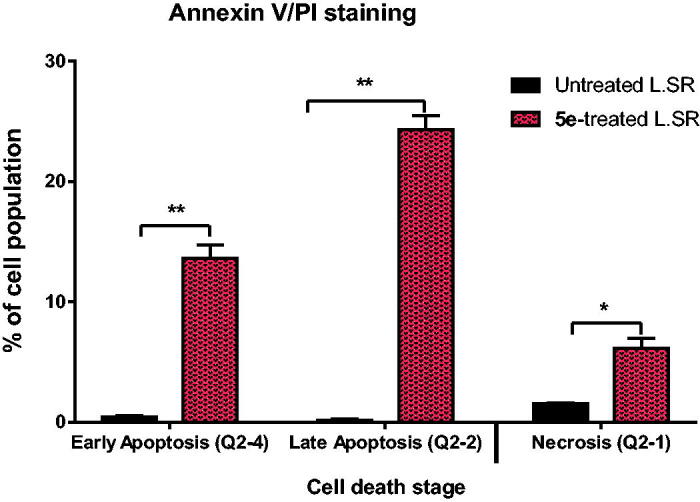
Induction of apoptosis by compound **5e** in SR cells shows both apoptotic (late and early) and necrotic cell death. **P < 0.001 and *P < 0.05 compared to control by GraphPad prism using unpaired *t*-test.

Annexin-V staining assay was applied to determine the apoptosis induced by compound **5e**. Thus, compound **5e** was added to SR cells with a concentration equivalent to its IC_50_ value of 13.05 ± 0.62 µM. According to the results represented in Figure 5, compound **5e** increased the apoptosis ratio by 37.34% compared to the untreated cells. In detail, 13.61% and 24.33% for the early phase and late phase of apoptosis, respectively, with respect to the control (0.44%, and 0.16%, respectively). Annexin V/PI staining histograms are supported in the supporting information (Figure SI 2).

#### In vivo studies

**Figure 6.**
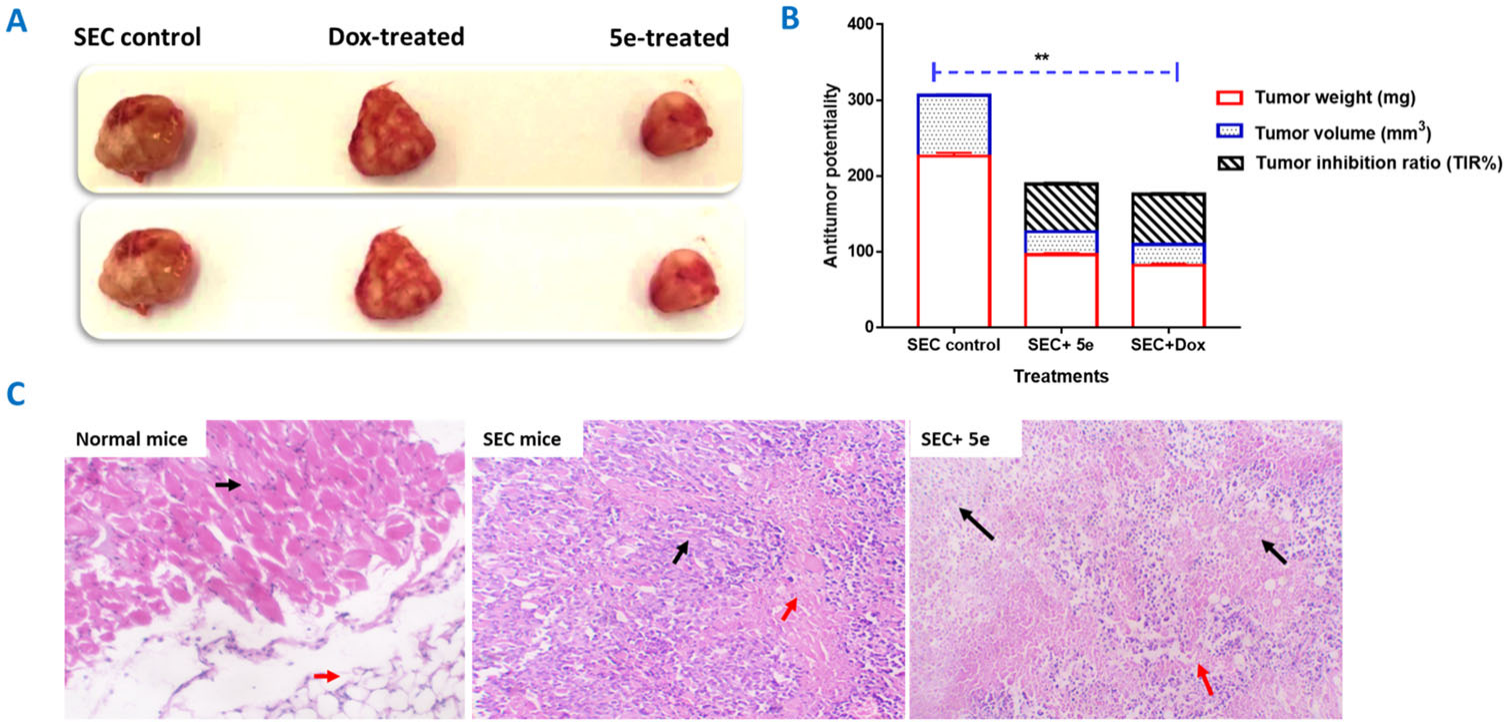
Solid tumour growth was inhibited in SEC-bearing mice by administration of compound **5e** (6 mg/Kg B.W., IP) (SEC model). (A) The tumour mass of different treated groups (morphological representation); “normal control, SEC-group, SEC+ **5e**, and SEC + Dox.” (B) Anti-tumour activity of tumour mass, volume, and TIR% in different treated groups. (C) Comparative morphological examinations of the tumour tissues tested groups. “Values are expressed as Mean ± SEM values of mice in each group (*n* = 7).” Values denoted by sign **(*P* ≤ 0.001) were found to be statistically different from the SEC control when compared with an unpaired *t*-test in GraphPad Prism. (H&E stain, magnification ×400).

Validating the anti-tumour activity of **5e** against Solid Ehrlich carcinoma (SEC) proliferation, tumour mass increased during the experimental period to 226 mg in the solid mass. Figure 6A,B shows that upon treatment both **5e** and doxorubicin had a significant anti-tumour effect, with a decrease in the solid tumour mass to 96.3 mg and 82.3 mg, respectively. Regarding tumour inhibition ratio, compound **5e** treatment significantly recorded a 62.7% compared to 65.8% (doxorubicin treatment). Moreover, compound **5e** decreased the tumour volume to 30.1 mm^3^ compared to 80.6 mm^3^ in the SEC-control, while doxorubicin treatment decreased tumour volume to 27.5 mm^3^.

Following the tumour reduction, histopathological examinations (Figure 6C) of tumour tissues in normal mice with uniform skeletal muscle bundles (Black arrow) with underlying fatty tissue (Red arrow). SEC group showing tumour (Black arrow) is formed of solid groups and irregular glandular structures, composed of malignant epithelial cells, with cellular and nuclear pleomorphism, nuclear hyperchromasia, and increased nucleo-cytoplasmic ratio. There are areas of necrosis (Red arrow). The SEC-treated group showed part of the tumour tissue (about 60%) (Black arrow) was necrotic (Black arrows). There are islands of viable tumour cells (Red arrows).

**Figure 7.**
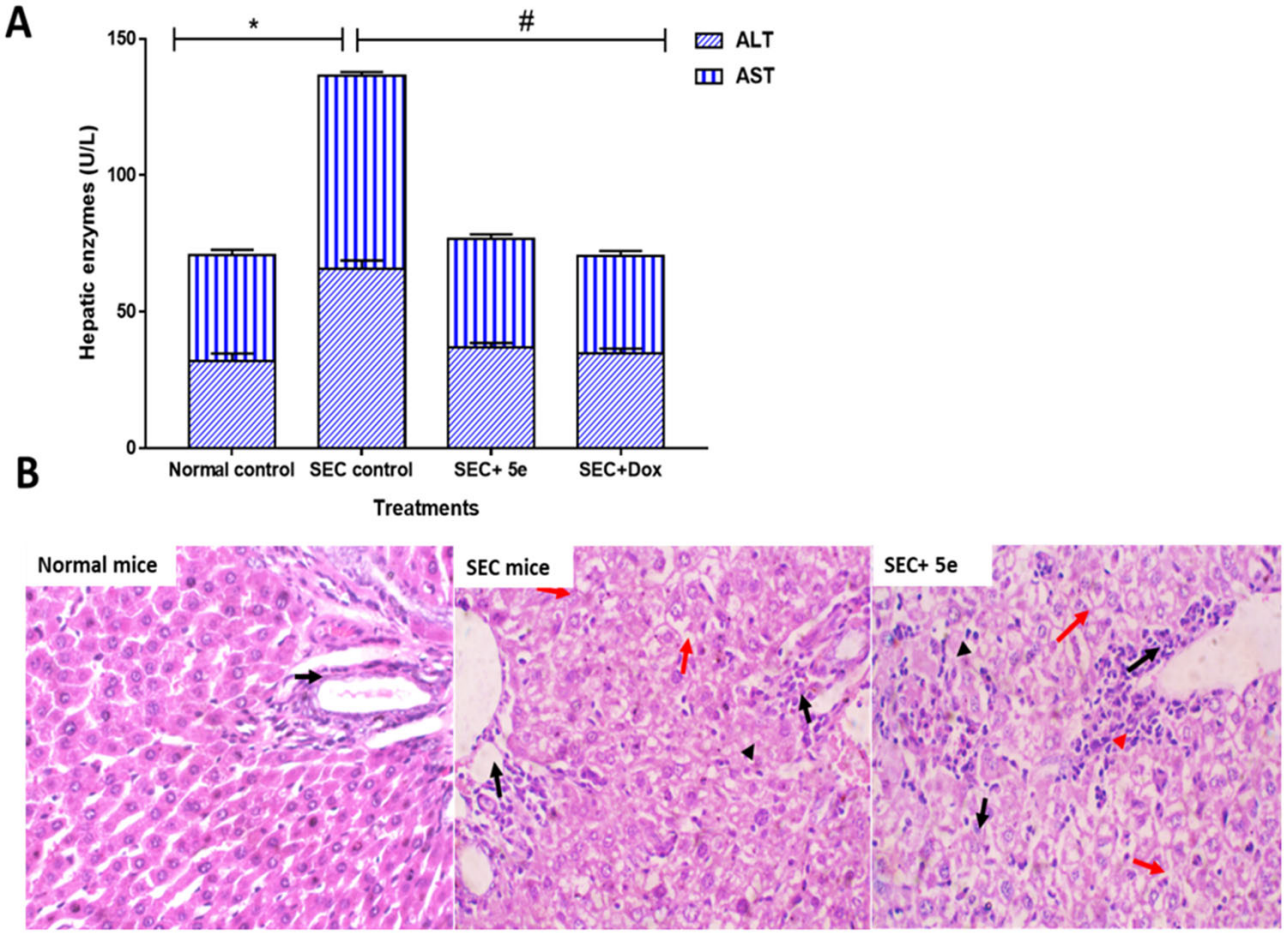
(A): ALT and AST in different SEC-treated groups. “Values are expressed as Mean ± SD of independent trials (*n* = 7),” “*(*P* ≤ 0.05) significantly different between SEC control and normal control, while ^#^(*P* ≤ 0.05) is significantly different between treated groups compared to SEC control.” (B) Histopathological examinations of liver tissues in different treated groups of SEC-bearing mice. (H&E stain, magnification ×400). Normal mice with uniform hepatocytes, and portal tract with uniform portal tract (Black arrows). SEC group show portal tract expansion with chronic inflammatory cells (Black arrows), area of lytic necrosis (Arrowhead), and hydropic degeneration of hepatocytes (Red arrows). Treated **5e** group show portal tract inflammation (Black arrow), with spillage of inflammatory cells into the limiting plate (Red arrowhead), foci of lytic necrosis, and mild lobular inflammation (Black arrowheads). Hepatocytes show mild hydropic degeneration (Red arrows).

ALT and AST (liver enzymes) dramatically elevated to 65.9 and 71 U/L, respectively, due to hepatocellular injury after tumour inoculation. After **5e**-treatment, both liver enzymes significantly lowered to 39.8 and 35.6 U/L, respectively, compared to doxorubicin treatment which decreased the ALT and AST levels to 34.8 and 35.6 U/L, Figure 7A. These findings demonstrated a significant enhancement of hepatocellular functions.

Interestingly, histological investigations of liver sections also indicated considerable improvements, correlating with the improvement in liver enzymes, Figure 7B.

Our results follow previous studies^49^^,^^50^, illustrating the anticancer activity of tested compounds through tumour inhibition, boosting liver enzyme activities, and repairing histopathological damage.

### In silico examinations

#### Molecular modelling

Discovery studio software 4.1^40^ was used to carry out a docking study *via* the selection of the X-ray crystallography of the human topoisomerase II-DNA complex from the PDB database (code: 3QX3). The selected poses of the most promising candidates were visualised using PyMOL program software^41^.

**Figure 8.**
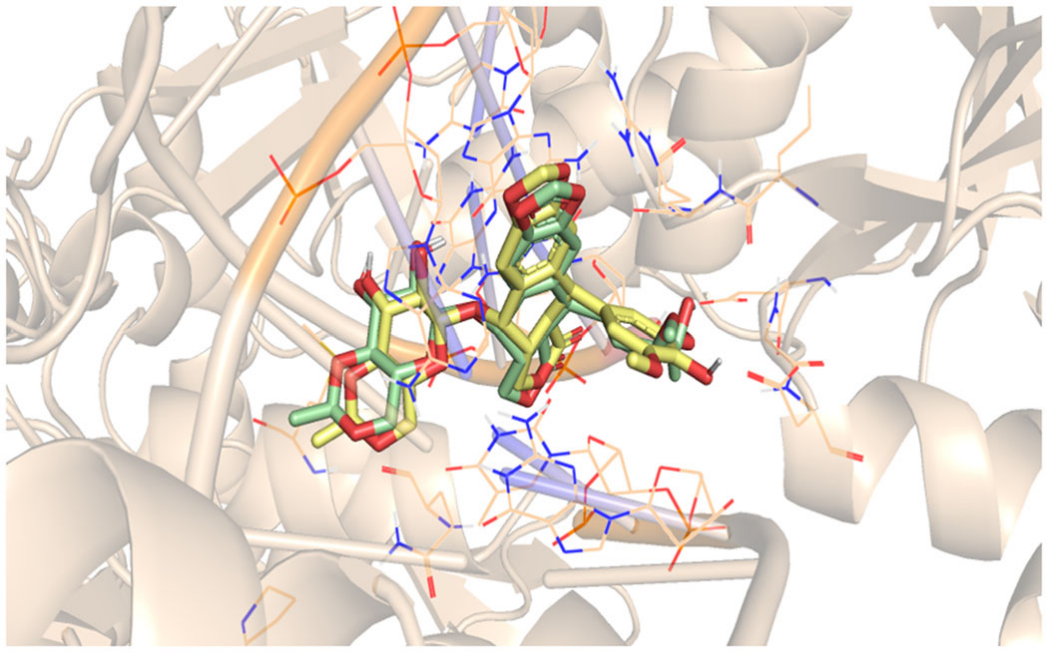
Re-docking of the **EVP** (green) and its docked pose (yellow).

Validation was performed to assure the accuracy of the Discovery studio program. This was accomplished by the re-docking of **EVP** (co-crystallized inhibitor) within the topoisomerase II-DNA pocket, Figure 8.

Molecular docking was applied for the synthesised derivatives to illustrate the binding modes and interactions against the topoisomerase II-DNA complex. Both **EVP** and doxorubicin were used as reference standards. The key binding sites of the topoisomerase II-DNA target comprise the amino acids; ASP479, GLN778, ARG503, and MET782, and the nucleobases; CYT8, ADE12, CYT11, GUA13, and THY9^16^.

Analysing the docking results of the examined candidates on the active site of topoisomerase II-DNA showed that most of the synthesised derivatives achieved good interactions. The docking scores of the closed analogues of dibenzo[*b,f*]azepin oxadiazole derivatives (**5a–g**) were found to be better than those of the dibenzo[*b,f*]azepine carbohydrazide open analogues (**4a–g**). The binding scores of all newly synthesised and examined compounds (**4a–g** and **5a–g**) are described in the supporting information (Table SI 1).

**Figure 9.**
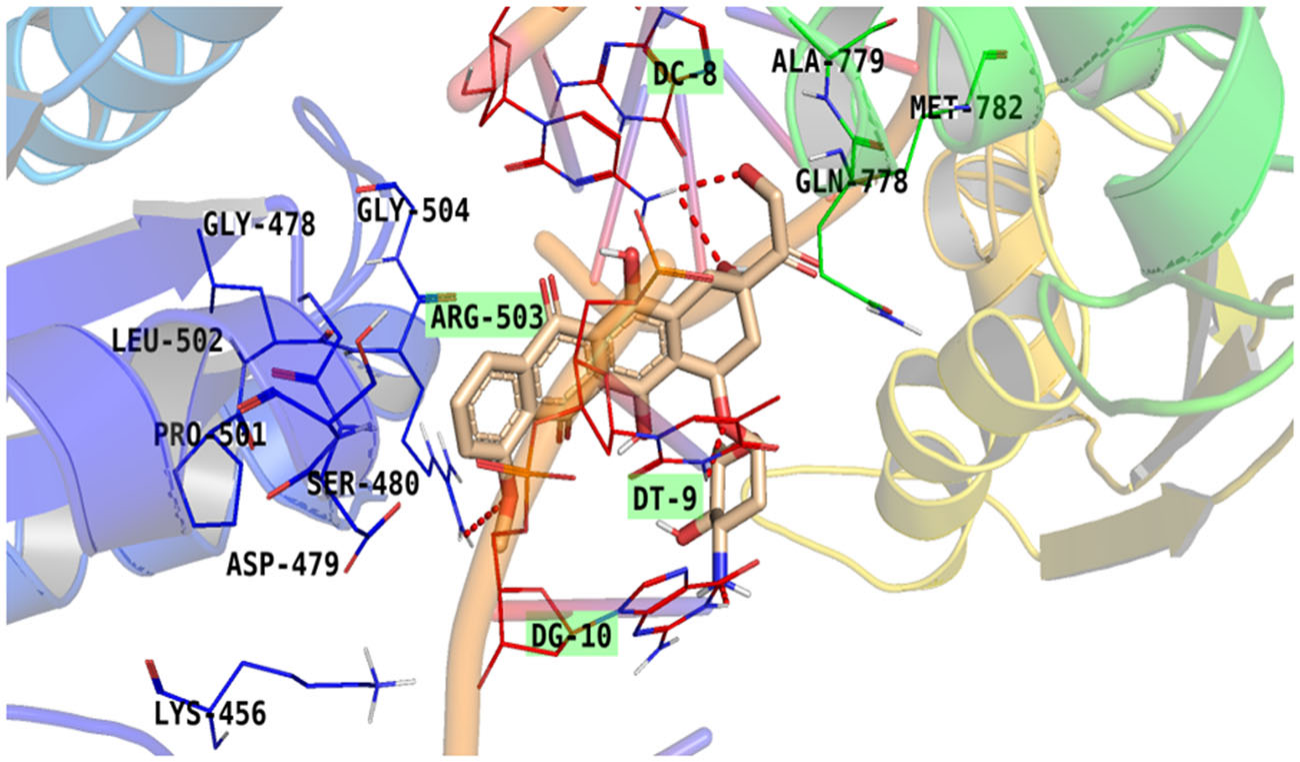
Binding of doxorubicin within the active pocket of topoisomerase II-DNA complex.

Doxorubicin, as a reference standard, showed good binding interaction and bound some key amino acids and nucleobases of the target receptor revealing its potentiality as a topoisomerase II inhibitor and DNA intercalator^16^. The amino group of doxorubicin (at C4 of the pyran ring) showed the formation of an H-bond with DG10. Additionally, the carbonyl group linked to the tetracene nucleus at C9 of doxorubicin is bound to DC9 *via* an H-bond. Moreover, the oxygen atom and hydroxyl group linked to C7 and C6 of the tetracene nucleus, respectively, formed H-bond with DT9. Besides, the oxygen atom of the methoxy group linked to C4 of the tetracene nucleus of doxorubicin was involved in H-bonds with ARG503, which is a crucial amino acid. The aromatic ring formed pi–alkyl interaction with ARG503 and pi–pi interaction with the second aromatic ring. Furthermore, doxorubicin-induced van der Waal interactions with GLY478, LEU502, LYS456, ASP479, GLN778, ALA779, and MET782, Figure 9.

**Figure 10.**
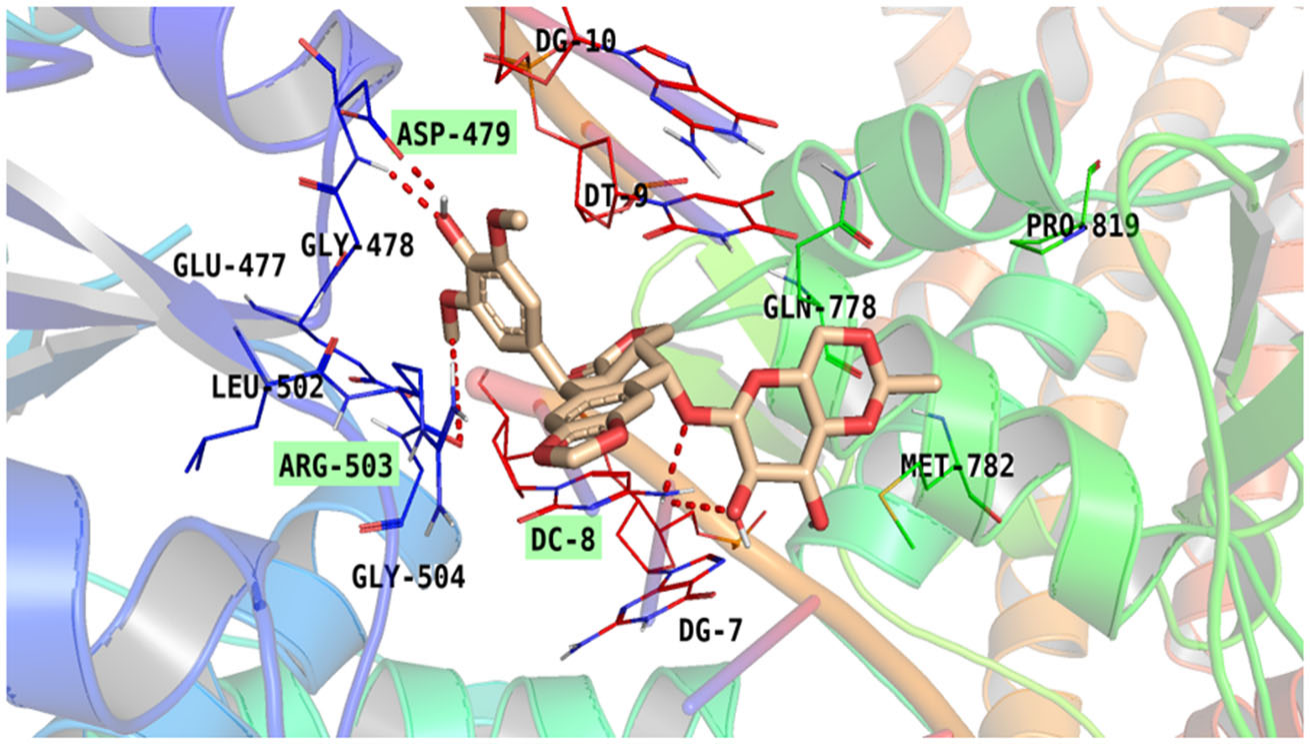
Binding of EVP within the active pocket of topoisomerase II-DNA complex.

Additionally, the co-crystallized **EVP** is bound with some key amino acids and nucleobases of the target receptor. The hydroxyl group (at C17 of the aromatic ring) formed two H-bond with ASP 479. Oxygen atom attached to pyranodioxin moiety formed H-bond with DC8. Also, a hydrogen atom of pyranodioxin moiety formed H-bond with DT8. Additionally, a methoxy group formed H-bond with ARG503. Also, methoxy group pyranodioxin formed pi-alkyl interaction with MET 782. Both aromatic rings formed pi-alkyl interaction with Arg 503. The methyl group of pyranodioxin nucleus formed a pi–alkyl bond with MET 782. Furthermore, it induced a van der Waal bond with GLU477, GLN778, and PRO819, Figure 10.

**Figure 11.**
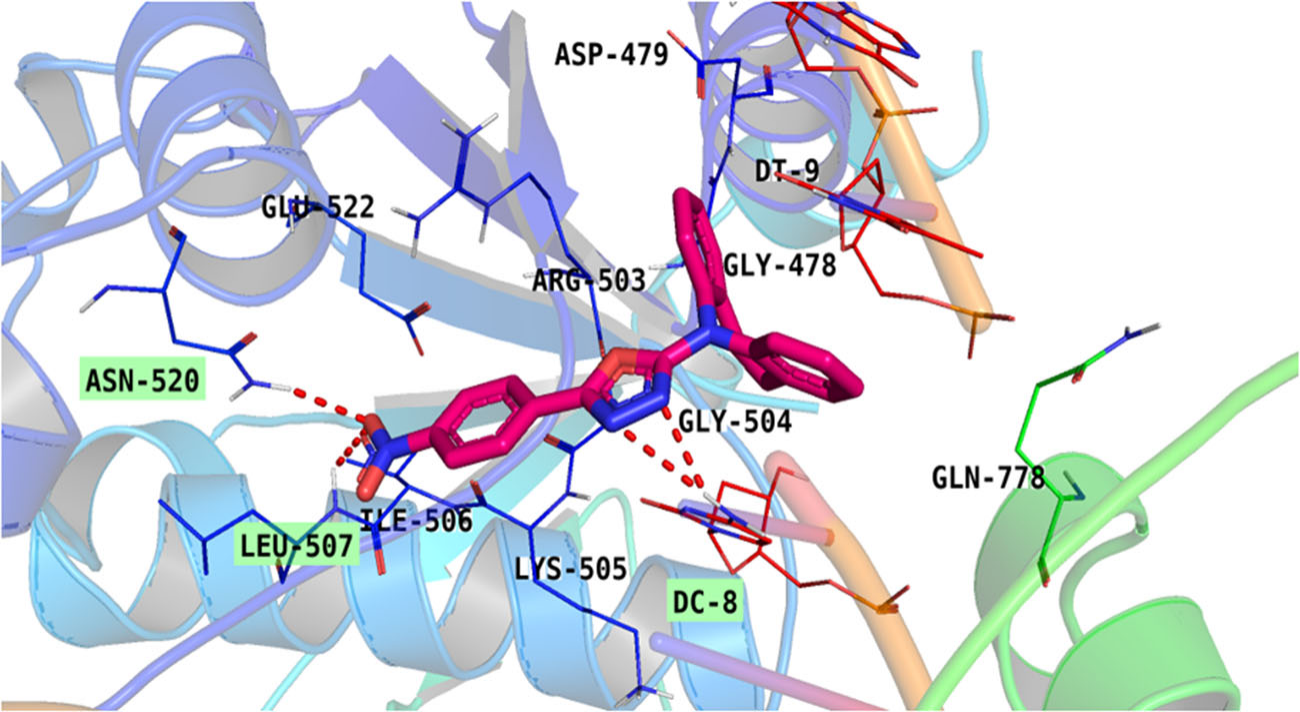
Binding of compound **5e** within the active pocket of topoisomerase II-DNA complex.

Compound **5e** was the most potent compound; it exhibited the best interaction against the topoisomerase II-DNA complex. oxadiazole moiety induced H-bond interaction through nitrogen atom with DC8 and pi-pi stacked interaction. One aromatic group attached to azepine formed a pi–pi bond with ARG503 and the other one formed a pi–pi interaction with DT9. Also, the oxygen atom of the nitro group formed two H-bonds with LEU507 and ASN520 as well as an attractive charge with GLU522. Furthermore, the compound induced van der Waal interaction with GLN778, GLY504, ASP479, DG10, ILE506, and LYS505 Figure 11.

**Figure 12.**
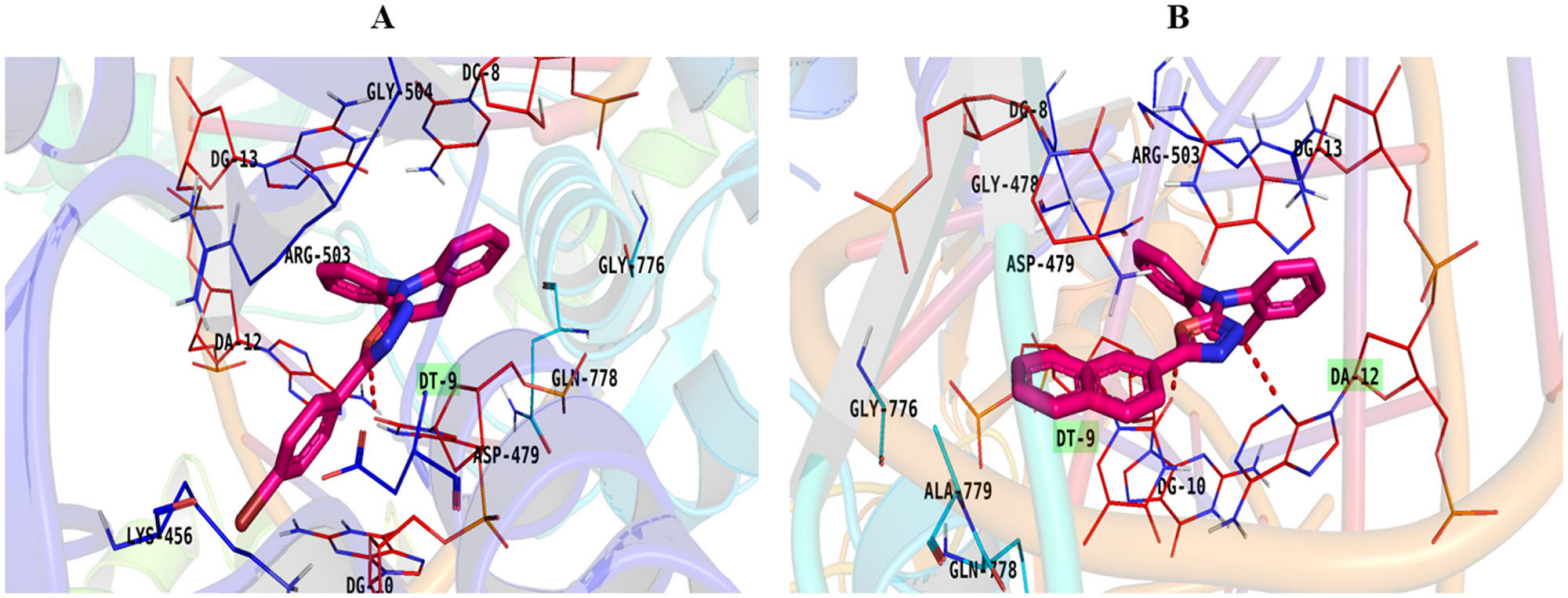
Binding of compounds **5b** (A) and **5f** (B) inside the topoisomerase II-DNA complex.

Moreover, the planner system of congeners **5b** and **5f** was also inserted between the DNA base pairs forming several pi–pi bonds. In addition, the oxadiazole moiety of compound **5b** formed an H-bond with DT9 (Figure 12A), while compound **5f** was stabilised in the active site *via* the formation of two H-bonds with DT9 and DT12 (Figure 12B).

Moreover, the 2D and 3D binding modes of the examined candidates **4a–g** and **5a–g** are represented in the supporting information (Table SI 2).

Briefly, the docking study revealed that series **5a–g** induced promising binding interactions on the active pocket of the topoisomerase II-DNA receptor and had the highest binding scores (Table SI 1), which may be due to cyclisation which confers certain rigidity and selectivity to the target receptor. Additionally, oxadiazole moiety exhibited good fitting to the receptor.

#### Physicochemical and pharmacokinetic properties

Simulation studies provide a reliable approach to investigating the molecule’s ability to act as a drug. Pharmacodynamic and pharmacokinetic properties were investigated online through the SWISSADME tool^46^. Fortuitously, our newly synthesised derivatives obeyed Lipinski’s rule, illustrating their ability to be an orally-active drug with consideration of bioavailability.

Concerning the ADME properties, all the newly synthesised target derivatives showed high GIT absorption. Therefore, these members may be considered to be orally active due to their low lipophilicity. Furthermore, most of the synthesised compounds can penetrate the blood–brain barrier (BBB); so these candidates are expected to be used for CNS tumours. Fortunately, most of the synthesised derivatives are negative substrates for P-glycoprotein (Pgp-) transporter and so are not exposed to its efflux action. Moreover, our target entities could be considered lead candidates for further optimizations in the future. Last but not the least; they exhibited zero alerts in the pan-assay interference structure (PAINS). The calculated pharmacokinetics and physicochemical parameters of the most active compounds (**5a–g**) are illustrated in Tables 4 and 5, respectively. Also, the pharmacokinetics and physicochemical parameters of compounds **4a–g** are represented in supporting information (Tables SI 3 and SI 4, respectively).

**Table 4.** Physicochemical parameters of the synthesised compounds **5a–g**.

Comp.	M.wt	NRB	HBA	HBD	TPSA (Å^2^)	Lipinski	Log *P*	Water solubility	MR
**5a**	371.82	2	3	0	42.16	Yes, 1 violation	5.09	PS	111.29
**5b**	351.40	2	3	0	42.16	Yes, 1 violation	4.89	PS	111.25
**5c**	382.37	3	5	0	87.98	Yes, 1 violation	3.98	MS	115.10
**5d**	387.43	2	3	0	42.16	Yes, 1 violation	5.38	PS	123.79
**5e**	351.40	3	3	0	42.16	Yes, 1 violation	4.66	PS	110.30
**5f**	387.43	2	3	0	42.16	Yes, 1 violation	5.38	PS	123.79
**5g**	351.40	3	3	0	42.16	Yes, 1 violation	4.66	PS	110.30

Num. of rotatable bonds (NRB); H. bond Acceptor (HBA); H. bond donor (HBD); Topological polar surface area (TPSA); Molar Refractivity (MR).

**Table 5.** Pharmacokinetic and ADMET parameters of the synthesised candidates **5a–g**.

Comp.	ADMET parameters					PAINS	Synthetic accessibility
	Log *K*_P_ (Skin permeation)	BBB permeant	CY1A2 inhibitor	GI absorption	p-gp substrate		
**5a**	–4.23 cm/s	Yes	Yes	High	No	0 alert	3.50
**5b**	–4.30 cm/s	Yes	Yes	High	No	0 alert	3.67
**5c**	–4.86 cm/s	Yes	Yes	High	No	0 alert	3.64
**5d**	–3.89 cm/s	No	Yes	High	No	0 alert	3.76
**5e**	–4.35 cm/s	Yes	Yes	High	Yes	0 alert	3.57
**5f**	–3.89 cm/s	No	Yes	High	No	0 alert	3.76
**5g**	–4.35 cm/s	Yes	Yes	High	Yes	0 alert	3.57

BBB, blood-brain barrier; GI absorption, gastrointestinal absorption.

## Structure–activity relationship

**Figure 13.**
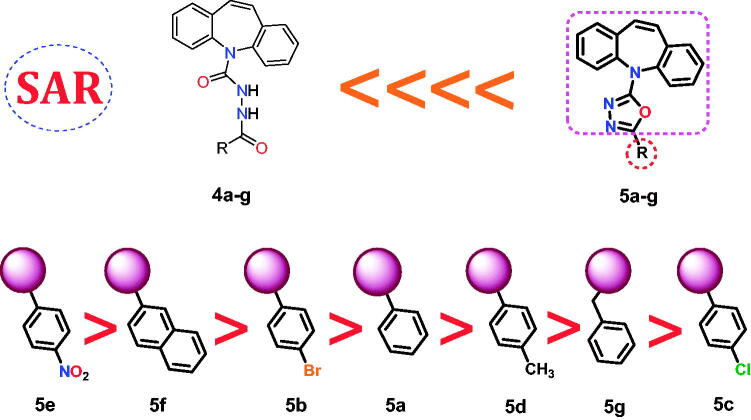
SAR for the newly synthesised derivatives **4a–g** and **5a–g** as topoisomerase II inhibitors and DNA intercalators.

A deep study of the results obtained from the biological testing as well as the docking simulation provided us with the following arrangement of the designed compounds as effective topoisomerase II inhibitors and DNA intercalators, Figure 13:

- It is worth mentioning that the dibenzo[*b,f*]azepin oxadiazole derivatives (**5a–g**) showed superior anticancer activities and docking scores compared to those of dibenzo[*b,f*]azepine carbohydrazide derivatives (**4a–g**).
- Therefore, regarding the above, we can say that the rigidification of (**4a–g**) compounds through their ring closure to produce the most promising anticancer candidates (**5a–g**) improved greatly their binding affinity and selectivity as well.
- The superior topoisomerase II inhibitory effect, besides the cytotoxic activity, was achieved upon linking the oxadiazole part with a nitrophenyl moiety (**5e**). However, the binding pattern of compound **5e** into the active pocket of the enzyme strongly supported its high biological activity.
- Except for the nitrophenyl-containing compound **5e**, the bicyclic groove binding bearing member **5f** possessed a greater topoisomerase II inhibiting effect than that of the monocyclic groove binding bearing members.
- Comparing the halide-containing members revealed that the analogue with bromophenyl moiety **5b** showed a high biological activity than the chlorophenyl moiety bearing one **5c**.
- The unsubstituted phenyl ring of the groove-binding side chain **5a** increased the compound’s activity over the methyl-substituted phenyl **5d** or the benzyl side chain as well **5g**.

## Conclusion

In the present work, fourteen new dibenzo[*b*,*f*]azepine derivatives were designed and synthesised in a continuation of our previous findings to discover potent anticancer small molecules. Herein, the most promising seven anticancer candidates (dibenzo[*b,f*]azepin oxadiazole derivatives (**5a–g**)) were designed and synthesised by molecular rigidification using the ring closure technique of another corresponding new seven open analogues (dibenzo[*b,f*]azepine carbohydrazide derivatives (**4a–g**)). The new counterparts were designed to achieve the basic requirements of the reported topoisomerase II inhibitors and DNA intercalators. The NCI-60 protocol was assigned to study the effect of the designed members on various cell lines. Results revealed the promising effect of the newly designed closed analogues (**5a–g**) on the leukaemia SR cell lines. Following, compounds **5a–g** were examined for their potential to inhibit the topoisomerase II enzyme and doxorubicin was co-assayed as a reference drug. The obtained results confirmed the high efficacy of the designed members against the topoisomerase II enzyme. Notably, compound **5e** was the most potent among the tested members compared to doxorubicin. It inhibited the enzyme with an IC_50_ of 6.362 ± 0.36 µM versus 3.445 ± 0.19 µM of the reference drug. Additionally, the cytotoxicity effect of the **5e** member was evaluated against the SR cell line. Compound **5e** exhibited a significant cytotoxic effect with an IC_50_ value of 13.05 ± 0.62 µM. Moreover, the tested member **5e** inhibited the cell cycle in the G1 phase. Besides, compound **5e** increased the apoptosis ratio by 37.34% more than the untreated control cells. Finally, SAR and docking studies clarified that the rigidification of compounds (**4a–g**) through their ring closure to produce the most promising anticancer candidates (**5a–g**) improved greatly their binding affinity and selectivity as well. Also, the superior topoisomerase II inhibitory effect besides the cytotoxic activity was achieved upon linking the oxadiazole part with a nitrophenyl moiety (**5e**).

## Supplementary Material

Supplemental Material
