# Supplementary material for: Design and synthesis of novel rigid dibenzo[b,f]azepines through ring closure technique as promising anticancer candidates against leukaemia and acting as selective topoisomerase II inhibitors and DNA intercalators
Source: J Enzyme Inhib Med Chem. 2023 Jan 11;38(1):2157825. doi: 10.1080/14756366.2022.2157825 (PMC9848257; doi:10.1080/14756366.2022.2157825)
Supplement: Supplemental Material [file IENZ_A_2157825_SM1466.pdf]

Design and Synthesis of Novel Rigid Dibenzo[b,f]azepines Through Ring Closure Technique as Promising Anticancer Candidates Against Leukemia and Acting as Selective Topoisomerase II Inhibitors and DNA Intercalators

Mohammed Farrag El-Behairy[1], Walaa Hamada Abd-Allah[2], Mohamed M. Khalifa[3], Mohamed S. Nafie[4], Mohamed A. Saleh[5,6], Mohammed S. Abdel-Maksoud[7], Tarfah Al-Warhi[8], Wagdy M. Eldehna[9], and Ahmed A. Al-Karmalawy[10,*]

[1] Department of Organic and Medicinal Chemistry, Faculty of Pharmacy, University of Sadat City, Menoufiya 32897 Egypt.

[2] Pharmaceutical Chemistry Department, Collage of Pharmaceutical Science and Drug Manufacturing, Misr University for Science and Technology, P.O. 77, 6th of October City, Giza, Egypt.

[3] Pharmaceutical Medicinal Chemistry & Drug Design Department, Faculty of Pharmacy (Boys), Al-Azhar University, Cairo, Egypt.

[4] Chemistry Department, Faculty of Science, Suez Canal University, Ismailia 41522, Egypt.

[5] Department of Clinical Sciences, College of Medicine, University of Sharjah, Sharjah 27272, the United Arab Emirates.

[6] Department of Pharmacology and Toxicology, Faculty of Pharmacy, Mansoura University, Mansoura 35516, Egypt.

[7] Medicinal and Pharmaceutical Chemistry Department, Pharmaceutical and Drug Industries Research Institute, National Research Centre (ID: 60014618), Dokki, Giza, Egypt.

[8] Department of Chemistry, College of Science, Princess Nourah bint Abdulrahman University, Riyadh 84428, Saudi Arabia.

[9] Department of Pharmaceutical Chemistry, Faculty of Pharmacy, Kafrelsheikh University, Kafrelsheikh, Egypt.

[10] Pharmaceutical Chemistry Department, Faculty of Pharmacy, Ahram Canadian University, 6th of October City, Giza 12566, Egypt.

*Corresponding author:

Ahmed A. Al-Karmalawy: Email: akarmalawy@acu.edu.eg

ORCID: 0000-0002-8173-6073

Supporting information

SI 1. General chemistry protocol

All melting points were determined using the Electrothermal Capillary melting point apparatus and are uncorrected. Infrared (IR) spectra were recorded as KBr pellets with JASCO FT/IR-6100 Spectrometer and values are represented in cm^{-1}. ^{1}H NMR (400 MHz) and ^{13}C NMR (100 MHz) spectra were carried out on Jeol ECA 400 MHz spectrometer using TMS as the internal standard and chemical shift values were recorded in ppm on the δ scale. The ^{1}H NMR data were represented as follows: chemical shifts, multiplicity (s. singlet, d. doublet, dd. doublet of doublet, t. triplet, m. multiplet), and the number of protons. The ^{13}C NMR data were represented as chemical shifts. Mass spectral data were obtained by expression-coupled with express-plate Compact Mass Spectrometer (ESI, APCI/TLC/MS), Advion, USA. Silica gel TLC (thin layer chromatography) cards from Merck (silica gel precoated aluminum cards with the fluorescent indicator at 245 nm) were used for thin layer chromatography. Visualization was performed by illumination with a UV light source (254 nm). Column chromatography was carried out on silica gel 60 (0.063-0.200 mm) obtained from Merck.

SI 2. Spectral data of compounds (**4a-g** and **5a-g**), (IR, ^{1}H NMR, ^{13}C NMR, and Mass spectroscopy)

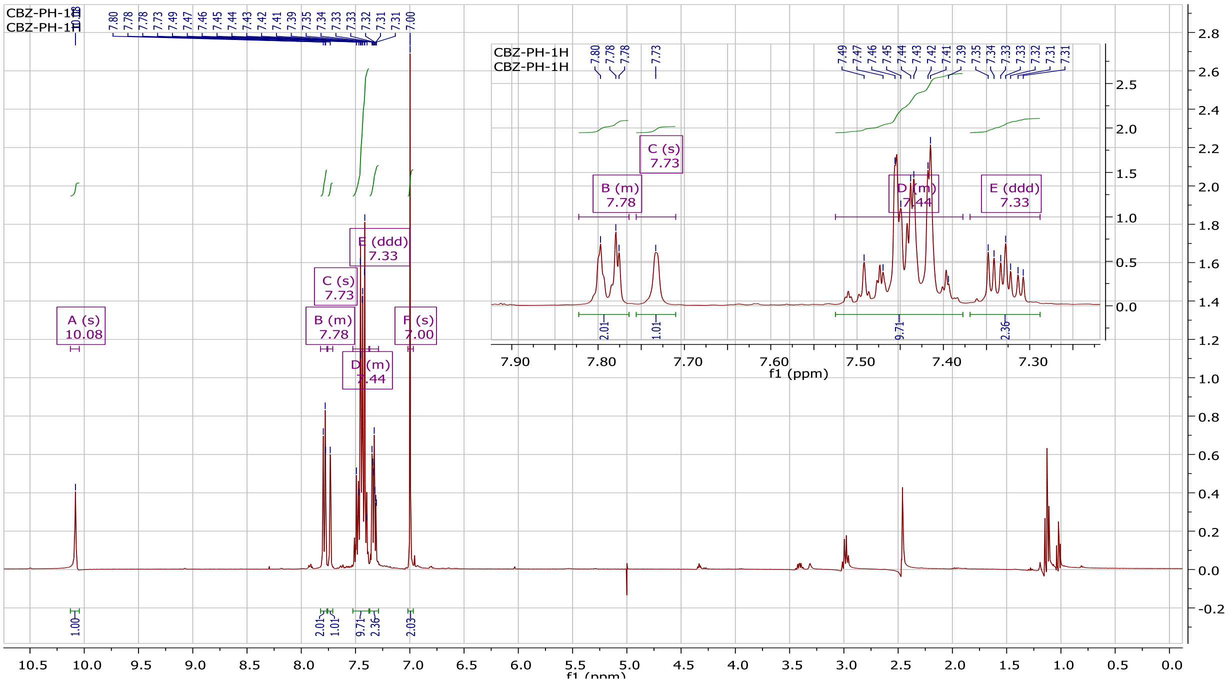

Figure S1: ^{1}H-NMR spectra of compound **4a**

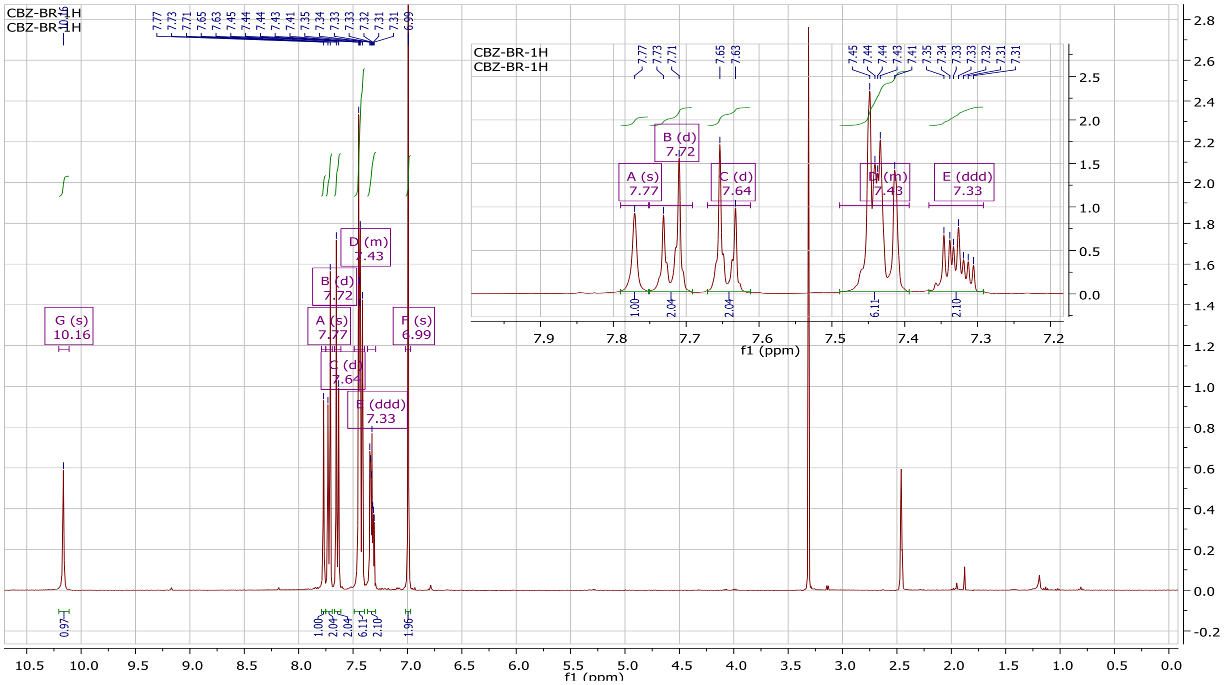

Figure S2: ^{1}H-NMR spectra of compound **4b**

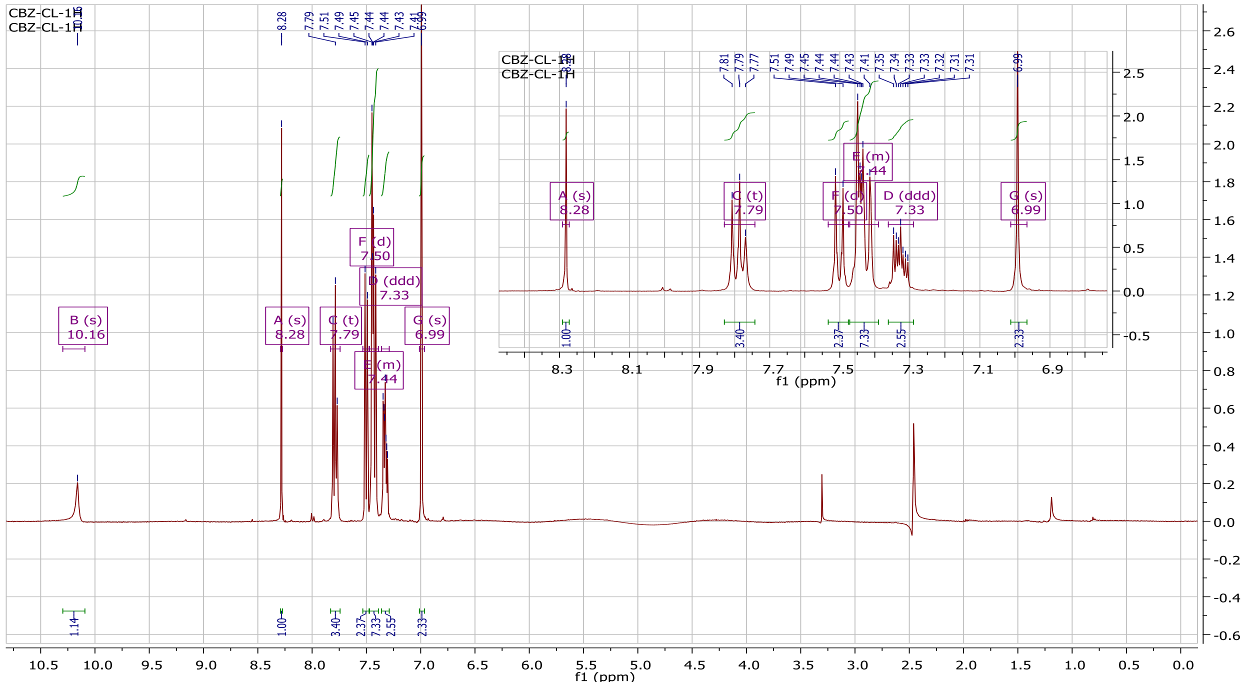

Figure S3: ^{1}H-NMR spectra of compound **4c**

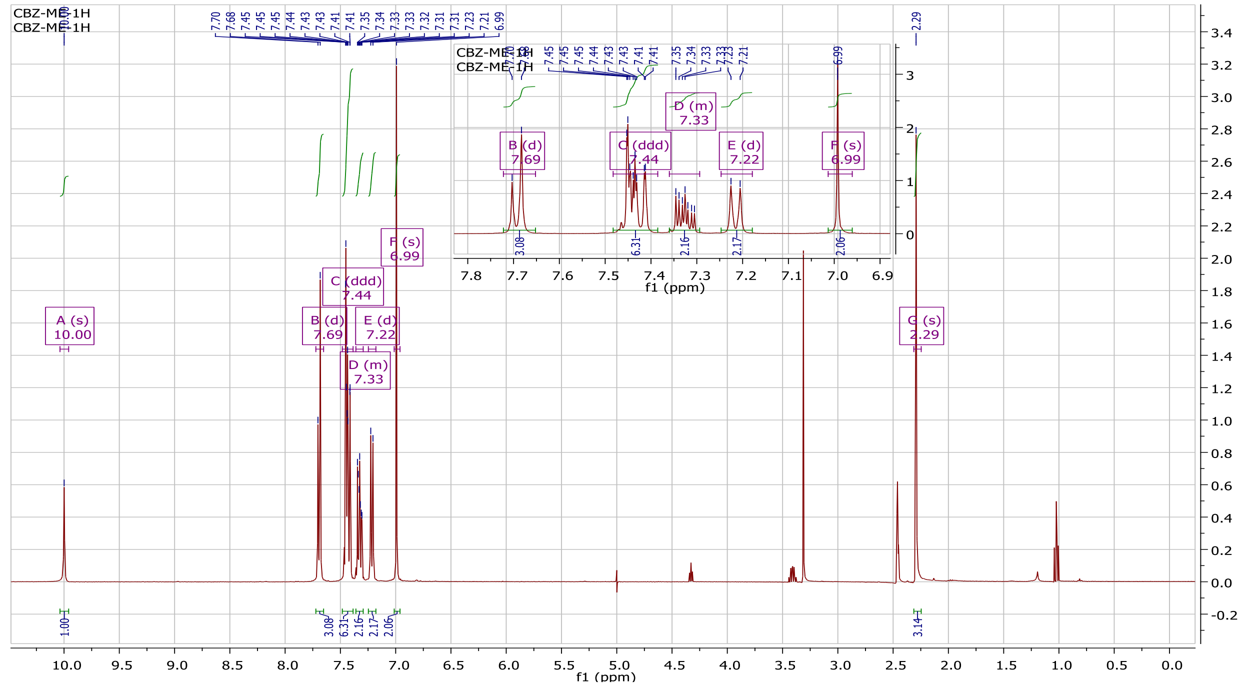

Figure S4: ^{1}H-NMR spectra of compound **4d**

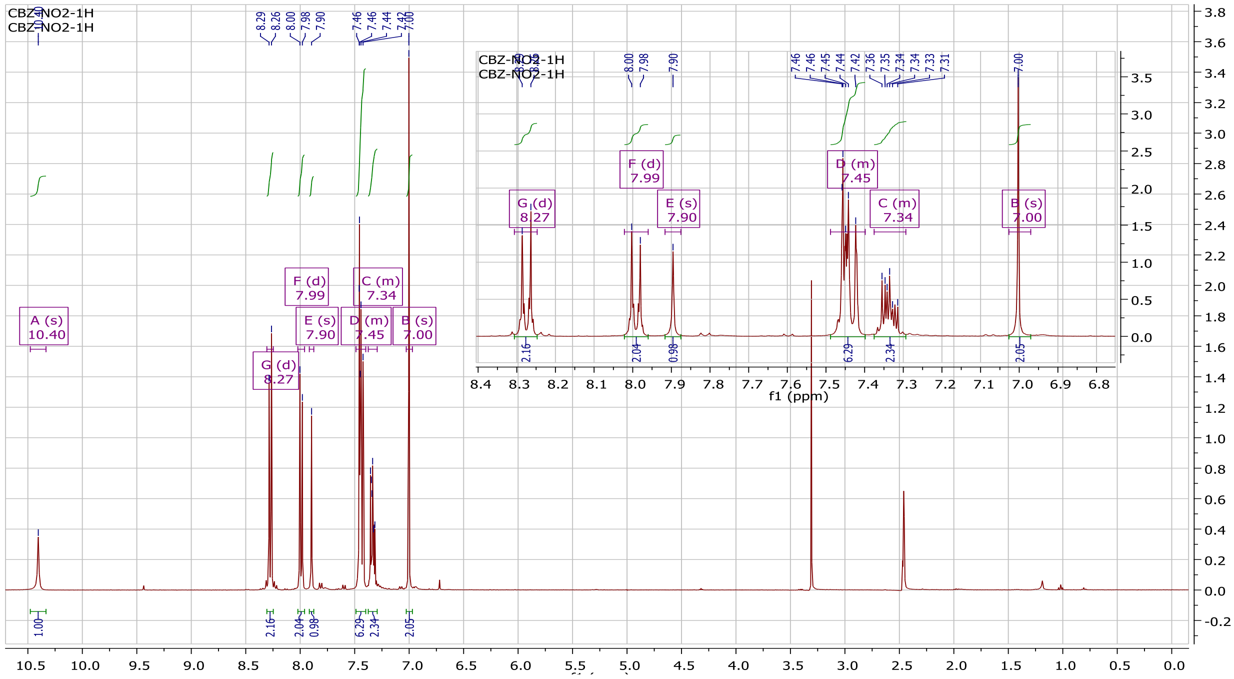

Figure S5: ^{1}H-NMR spectra of compound **4e**

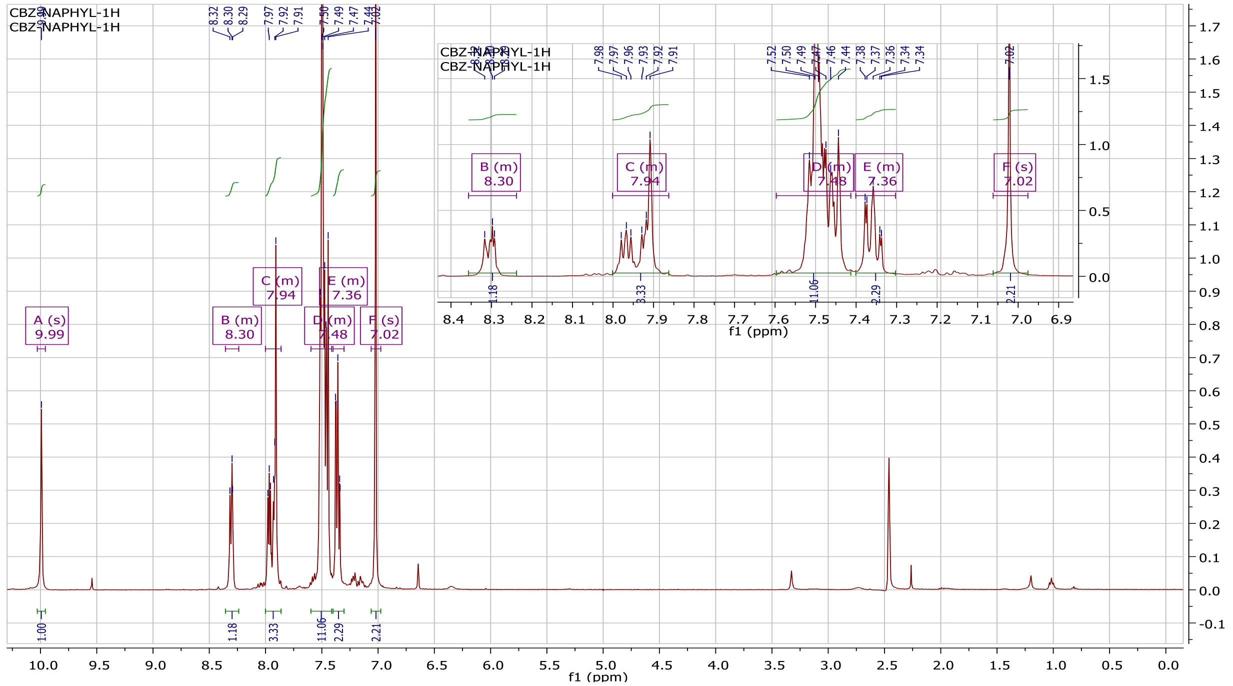

Figure S6: ^{1}H-NMR spectra of compound **4f**

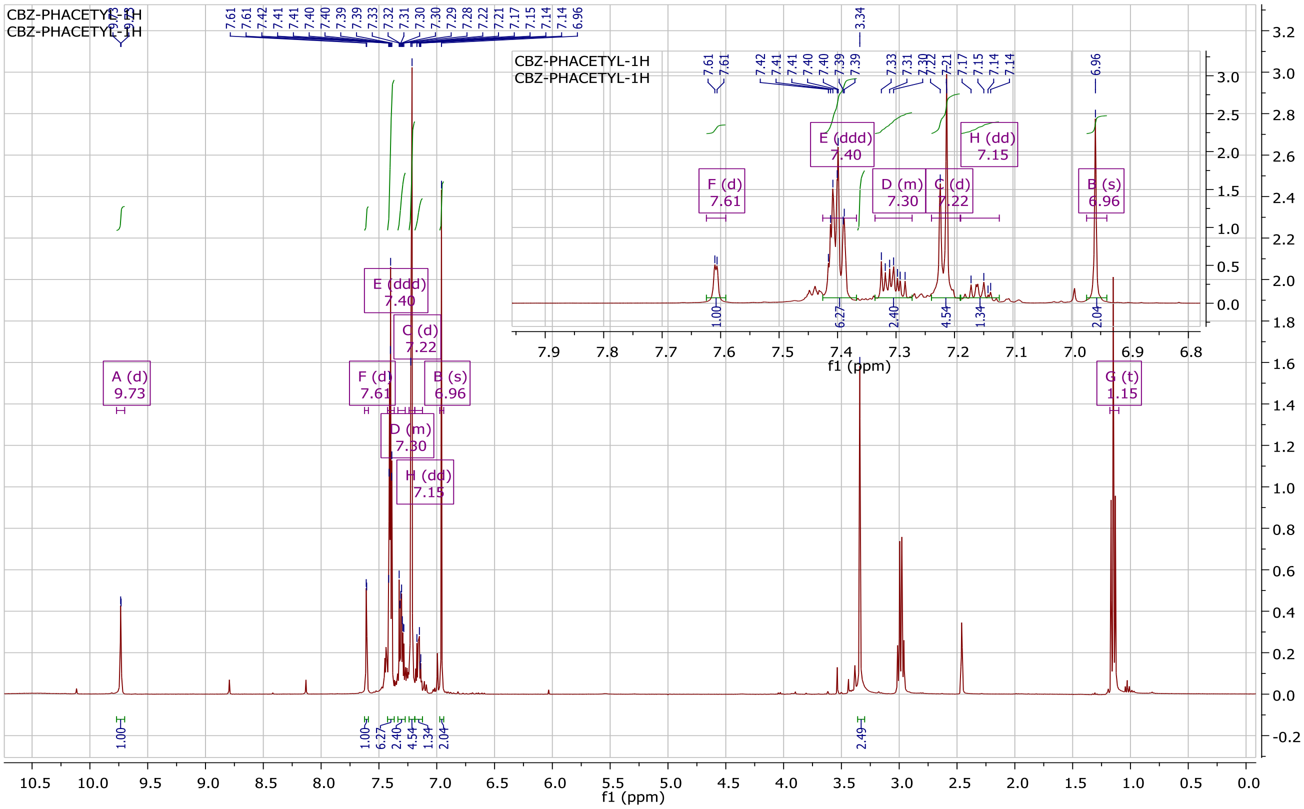

Figure S7: ^{1}H-NMR spectra of compound **4g**

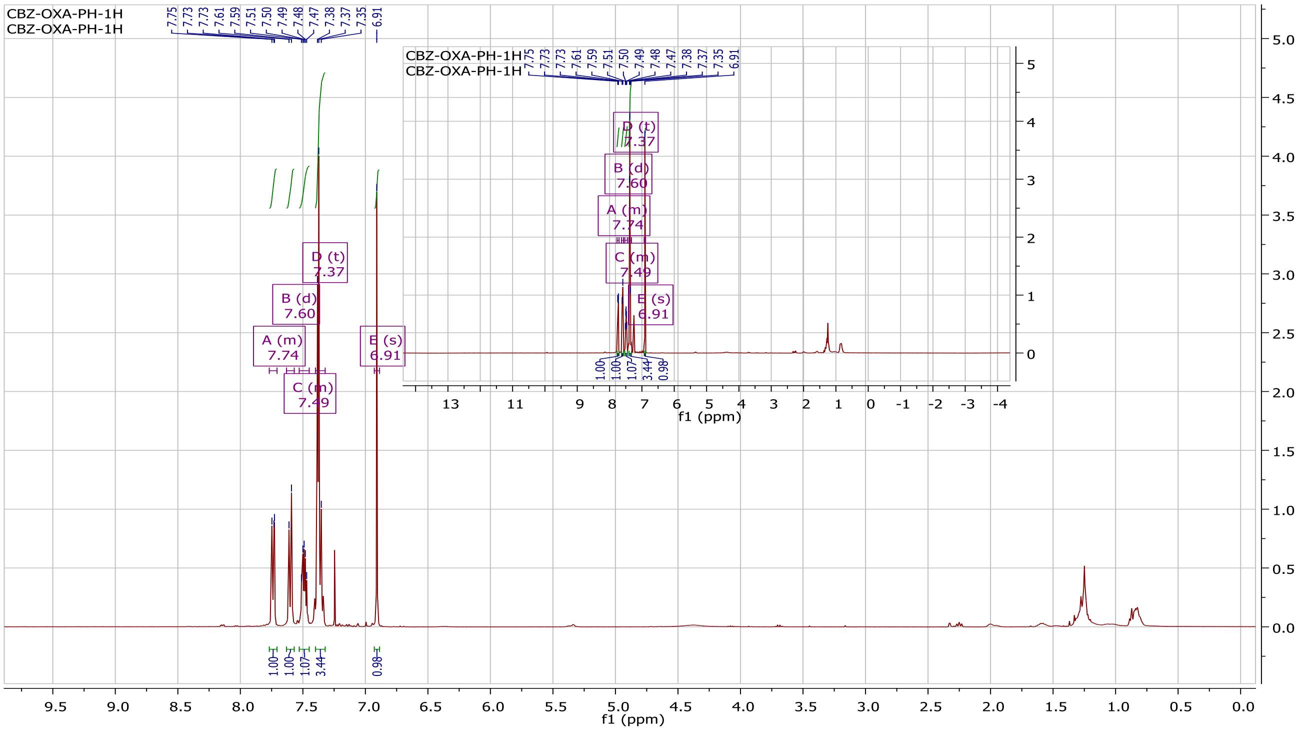

Figure S8: ^{1}H-NMR spectra of compound **5a**

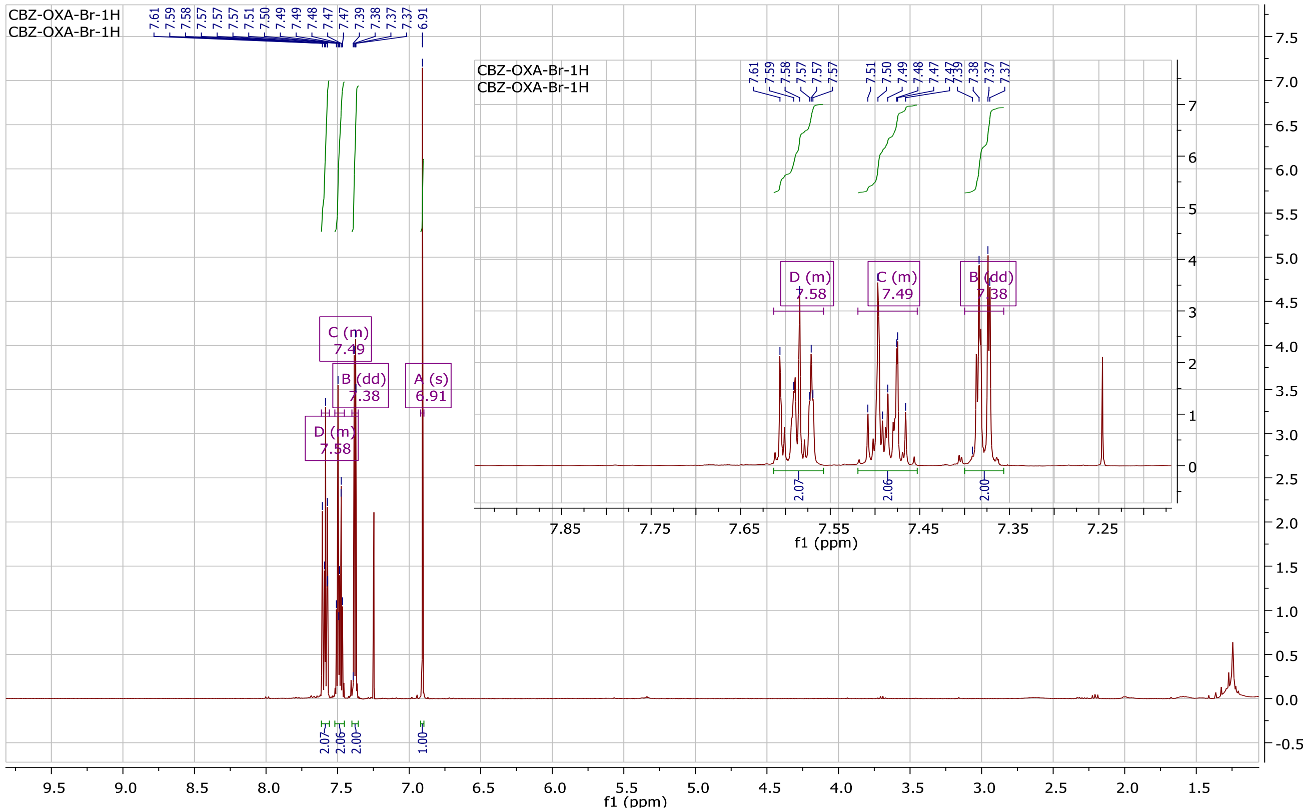

Figure S9: ^{1}H-NMR spectra of compound **5b**

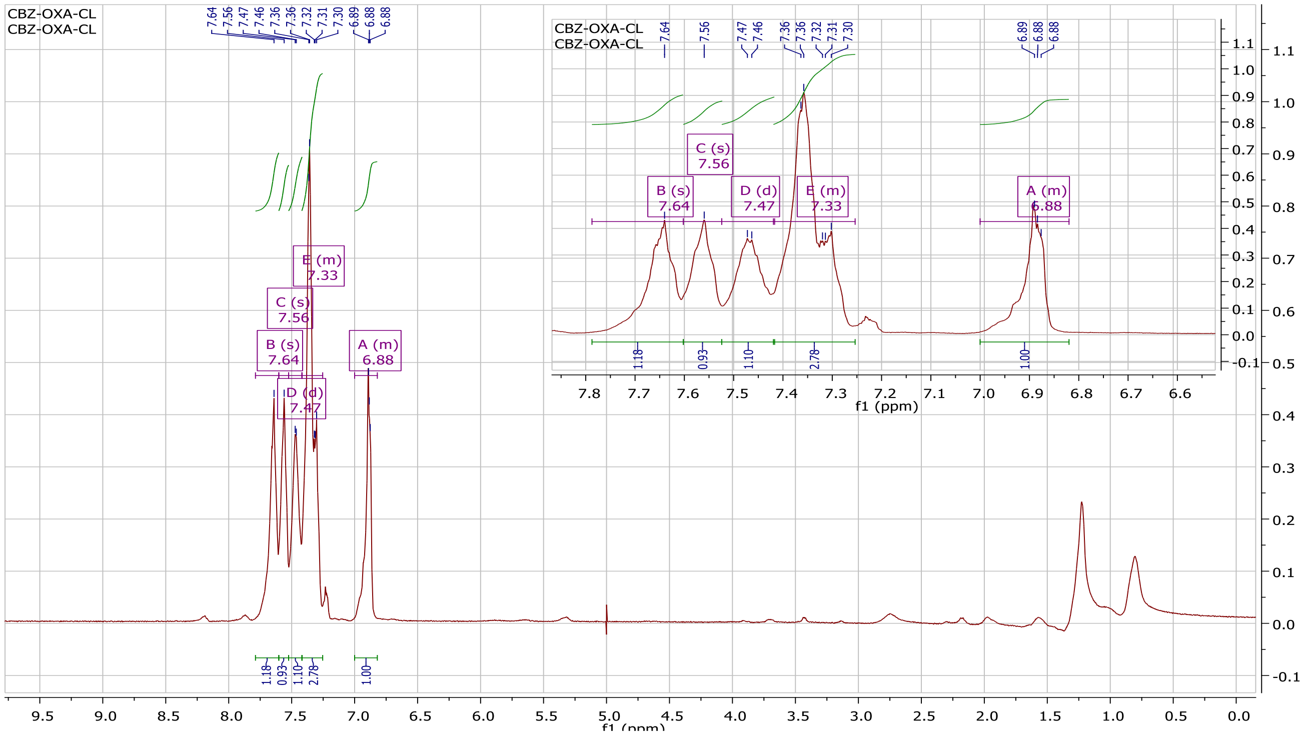

Figure S10: ^{1}H-NMR spectra of compound **5c**

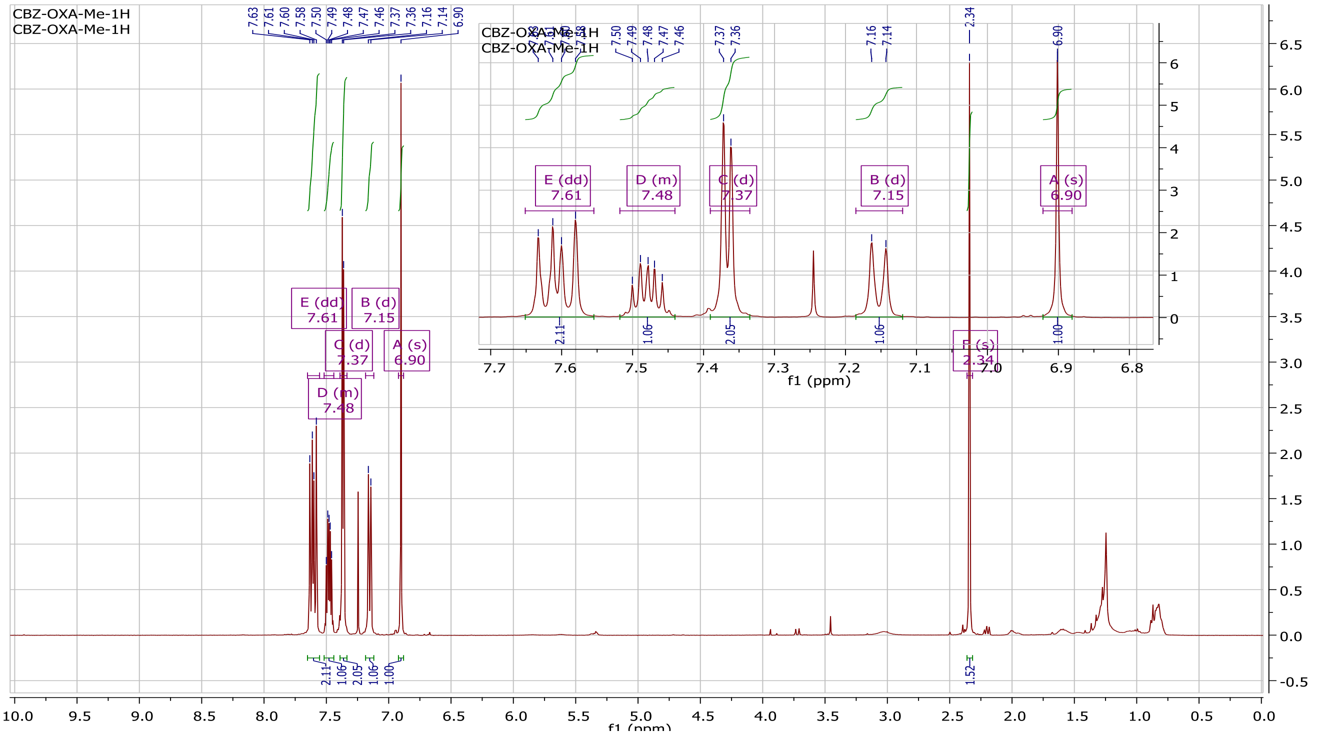

Figure S11: ^{1}H-NMR spectra of compound **5d**

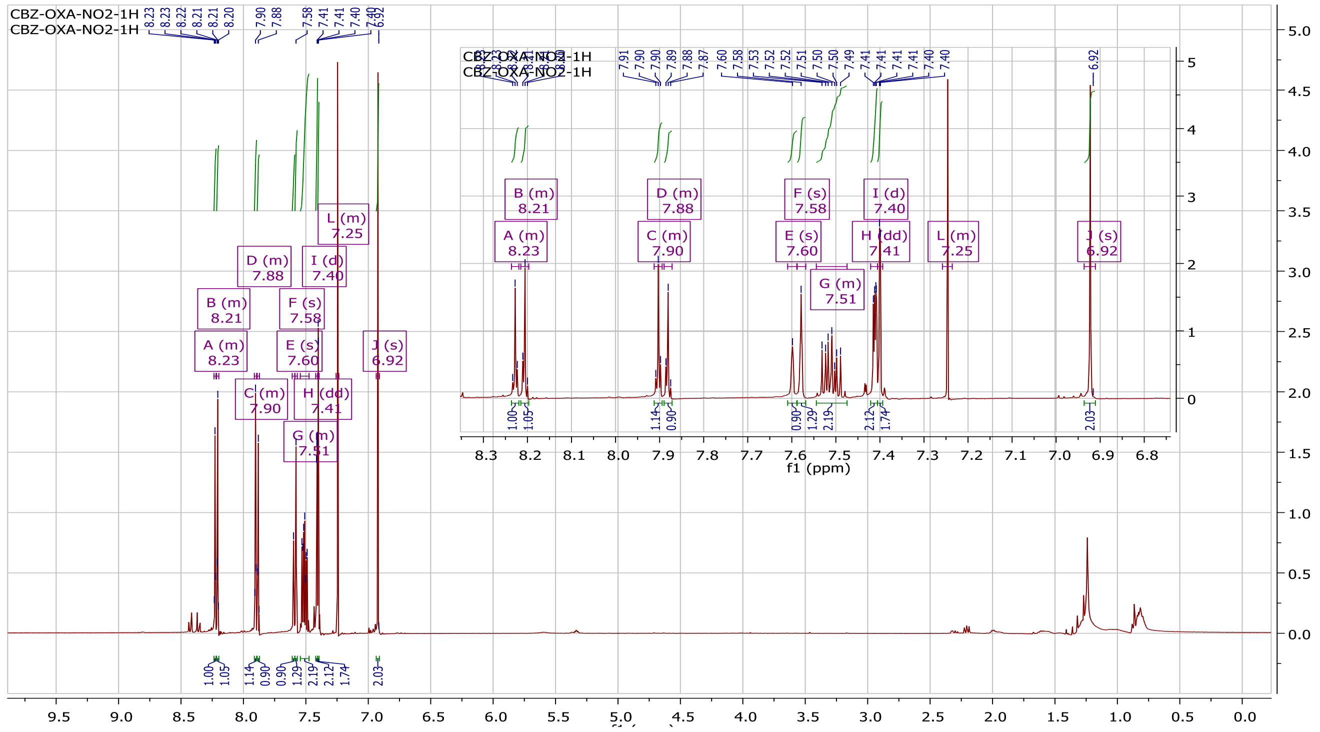

Figure S12: ^{1}H-NMR spectra of compound **5e**

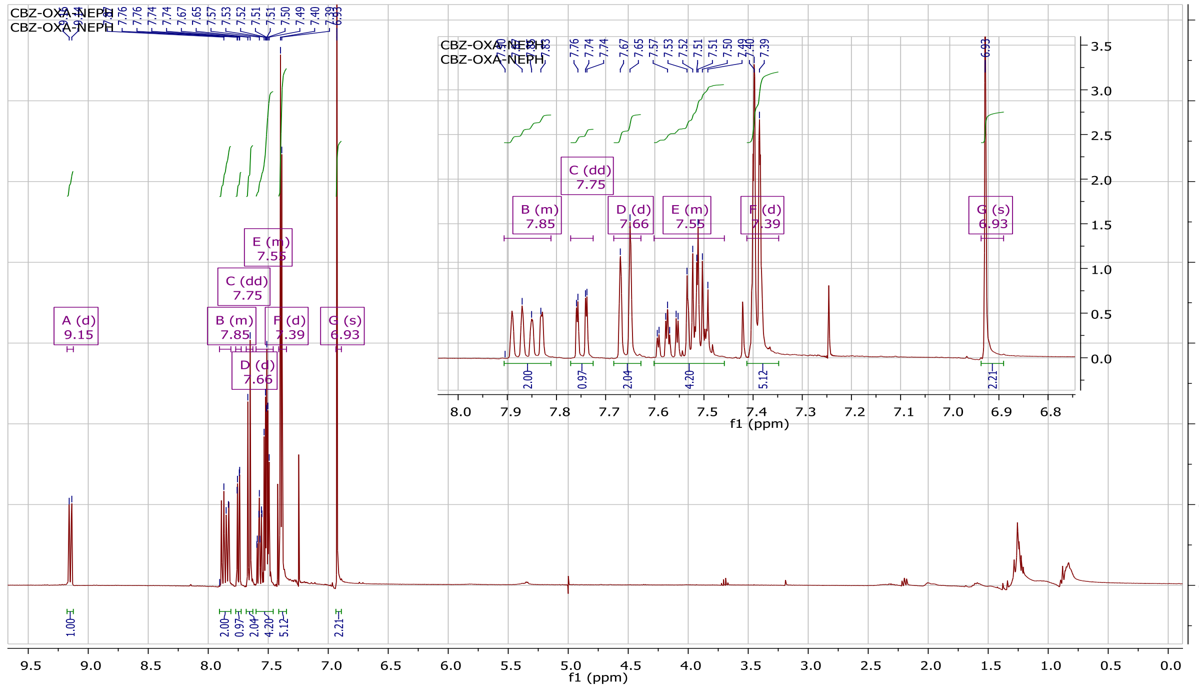

Figure S13: ^{1}H-NMR spectra of compound **5f**

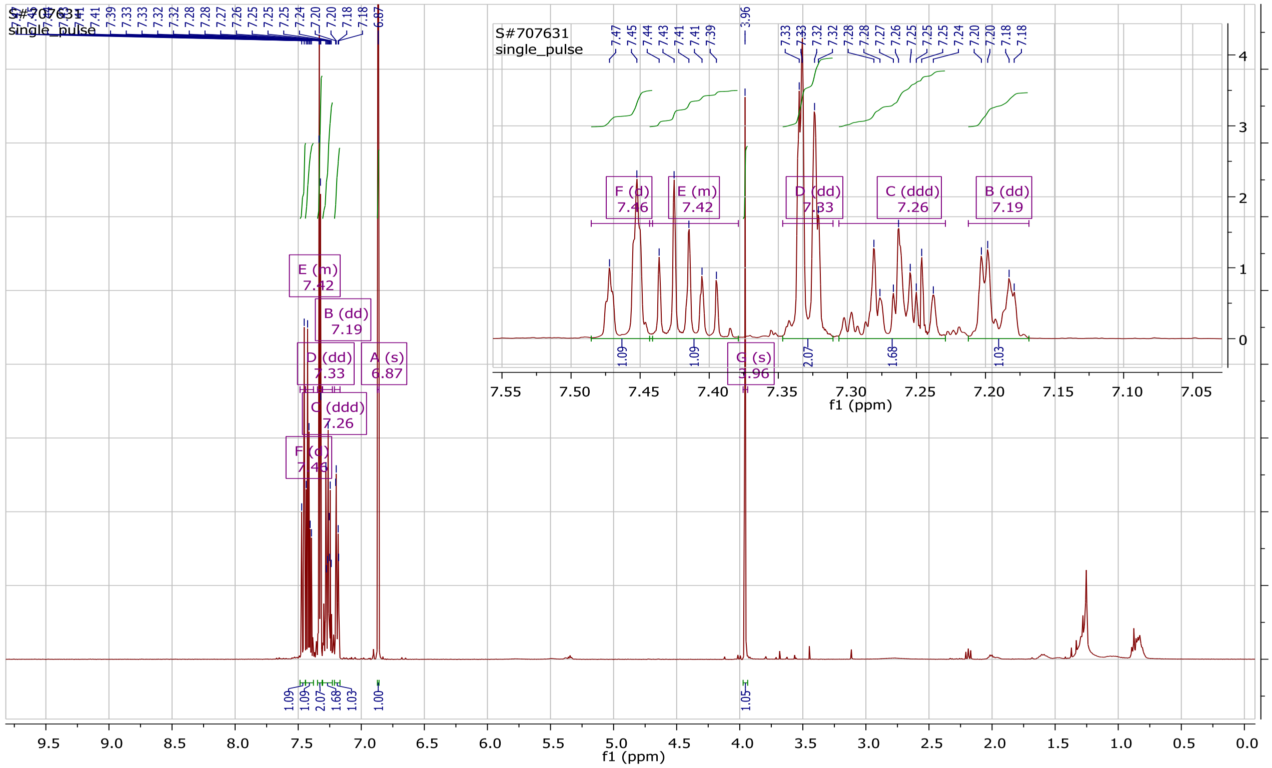

Figure S14: ^{1}H-NMR spectra of compound **5g**

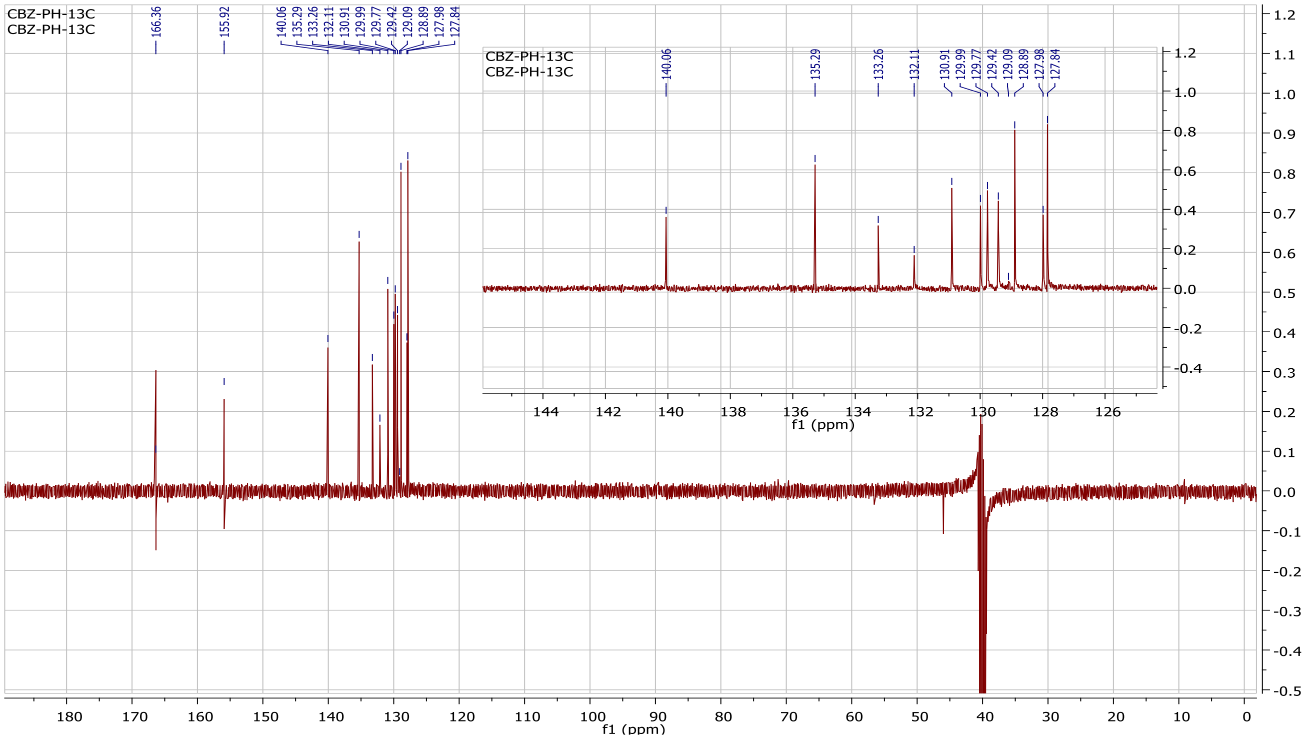

Figure S15: ^{13}C-NMR spectra of compound **4a**

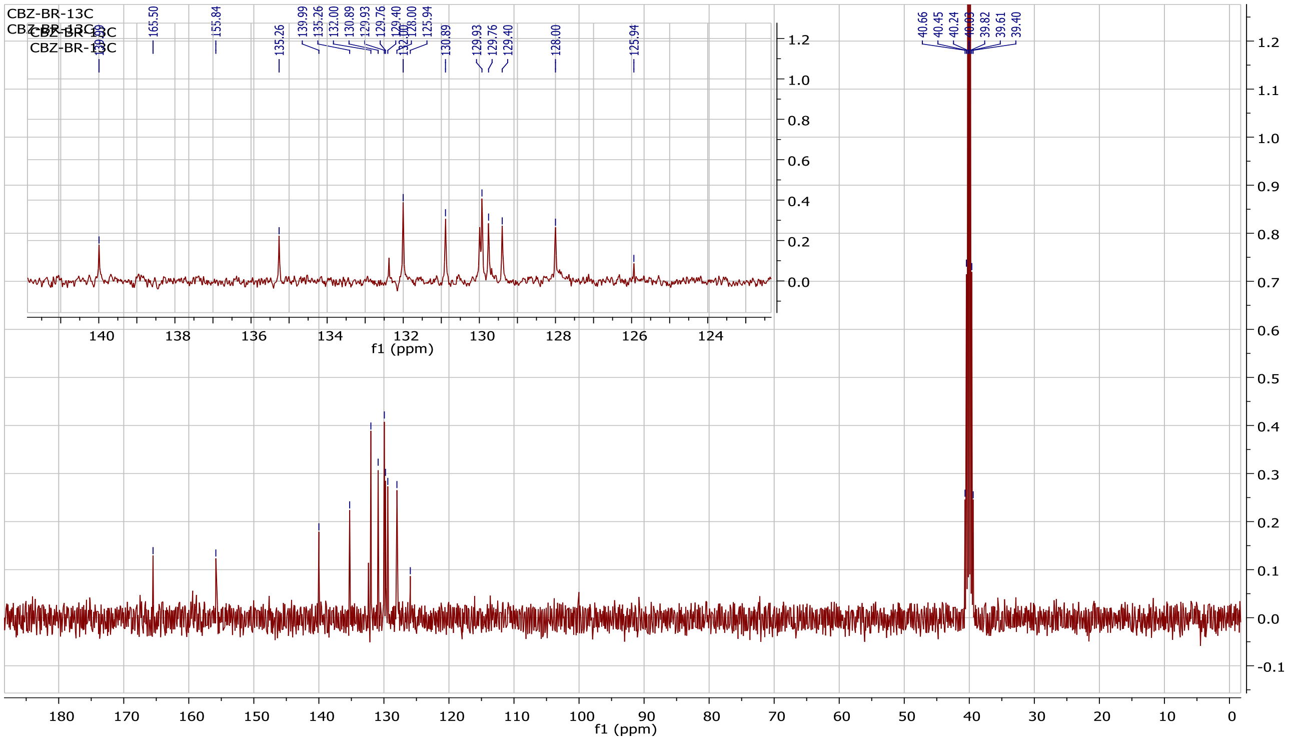

Figure S16: ^{13}C-NMR spectra of compound **4b**

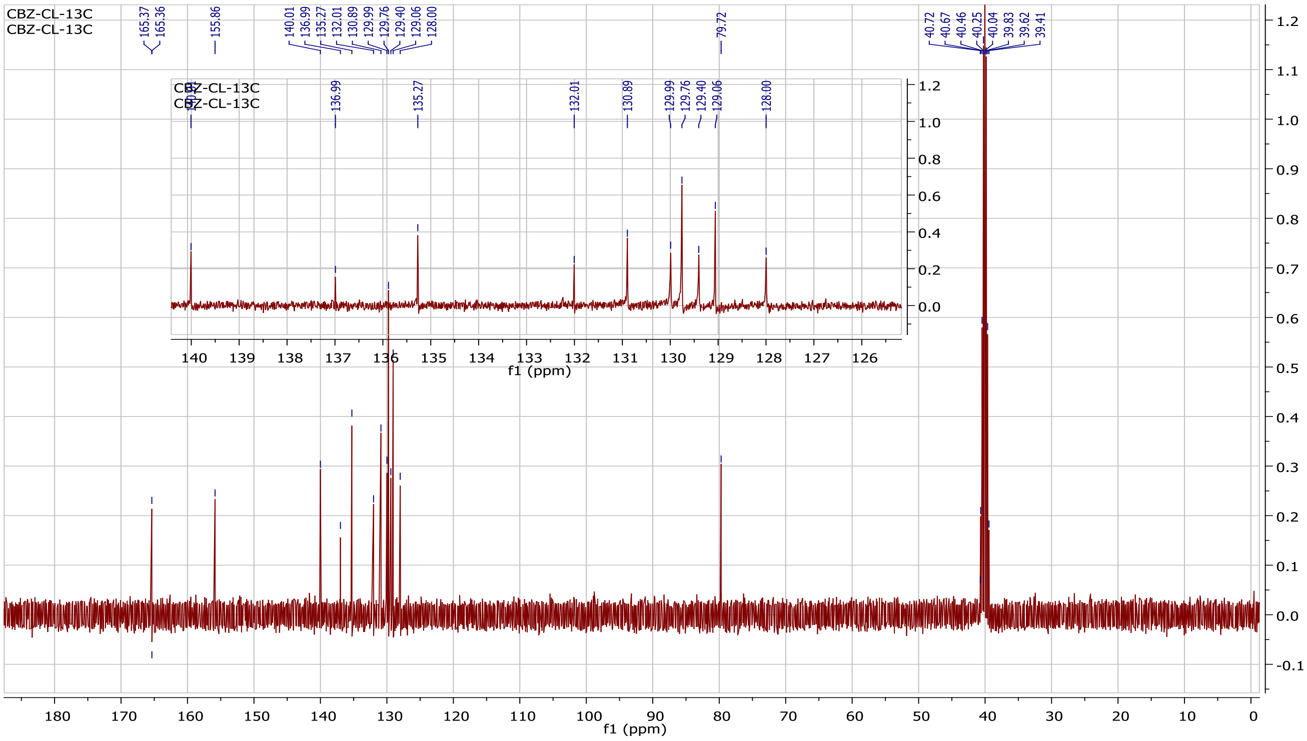

Figure S17: ^{13}C-NMR spectra of compound **4c**

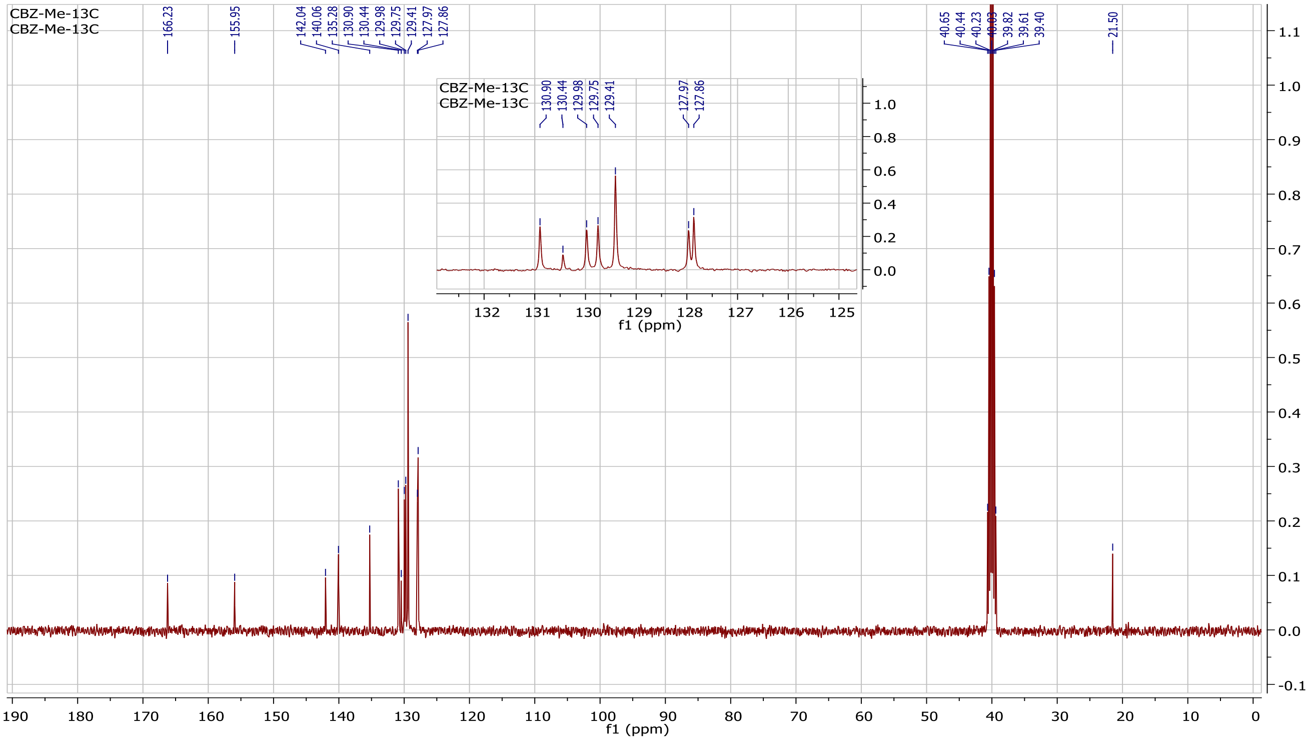

Figure S18: ^{13}C-NMR spectra of compound **4d**

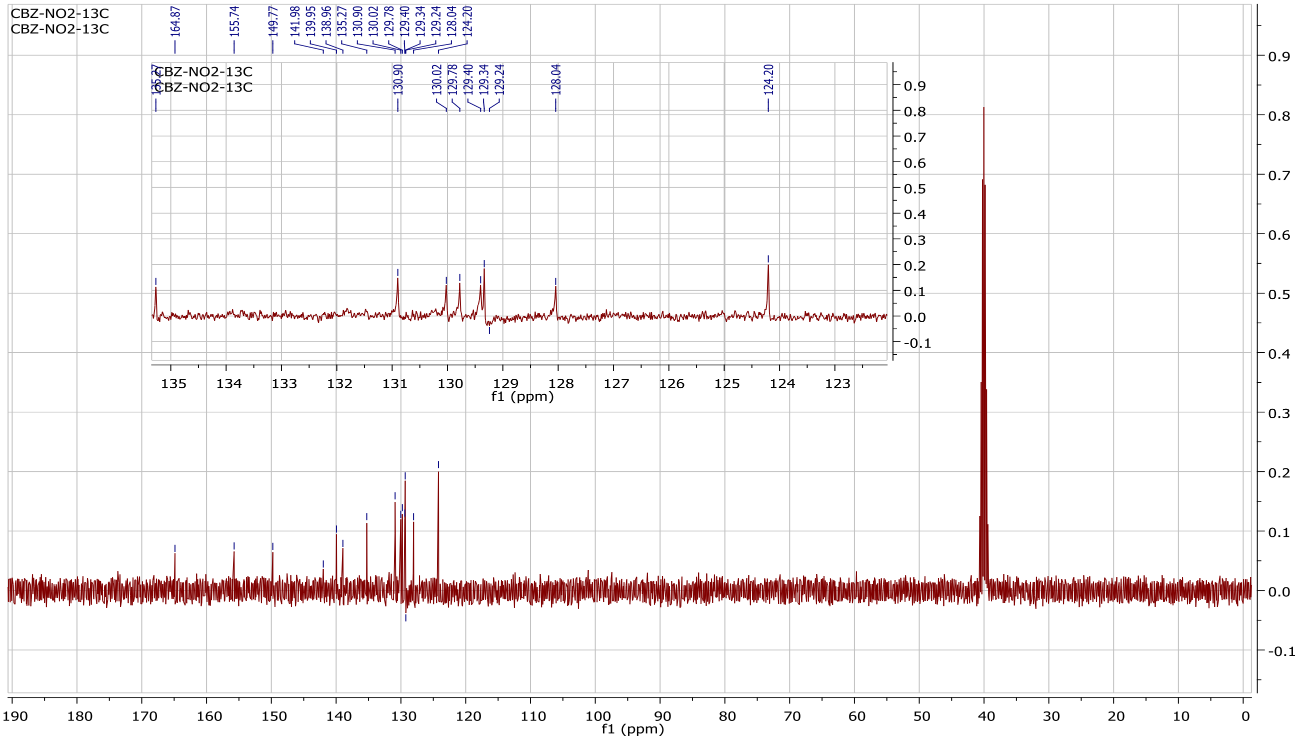

Figure S19: ^{13}C-NMR spectra of compound **4e**

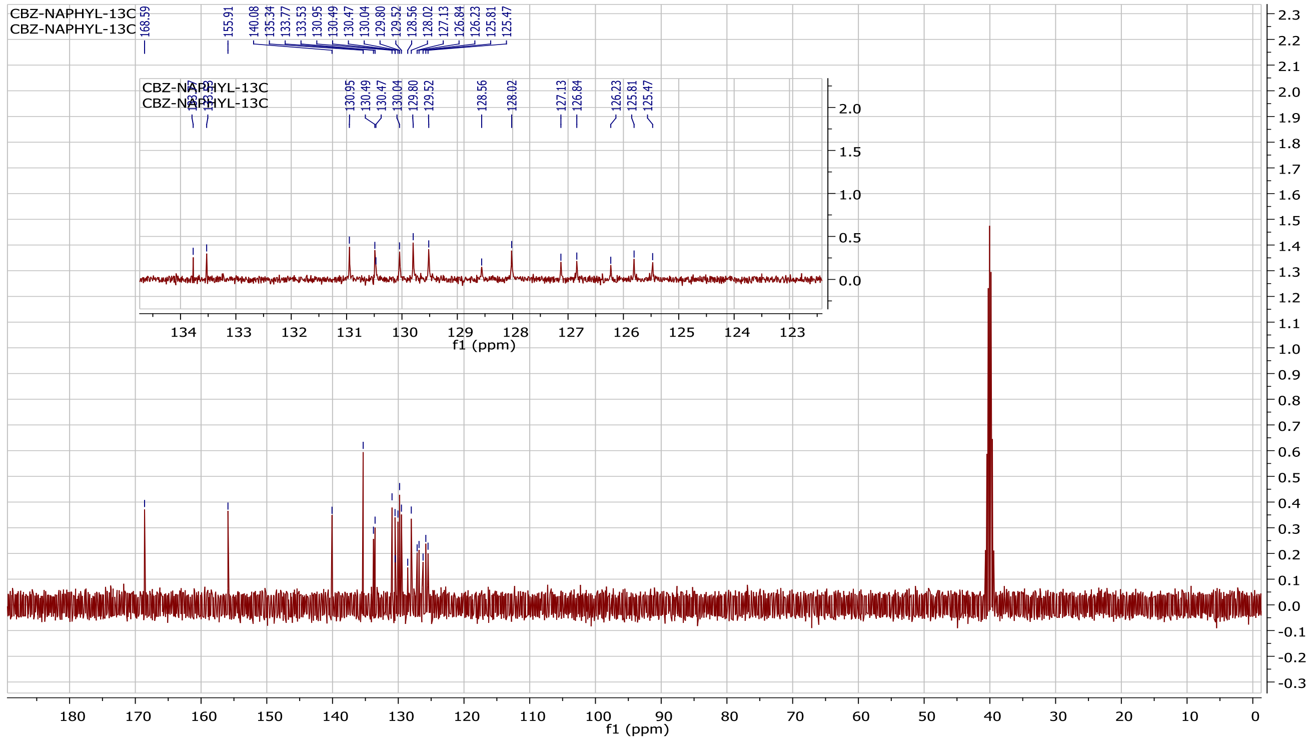

Figure S20: ^{13}C-NMR spectra of compound **4f**

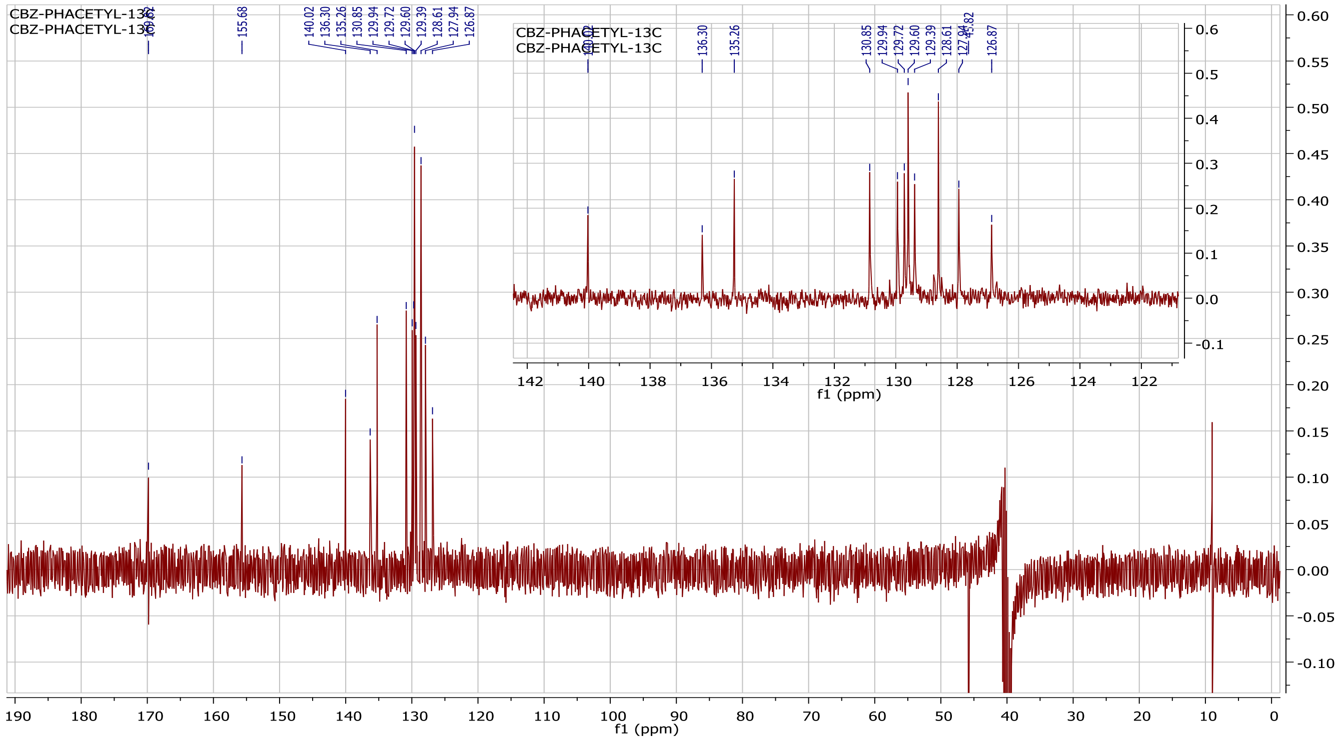

Figure S21: ^{13}C-NMR spectra of compound **4g**

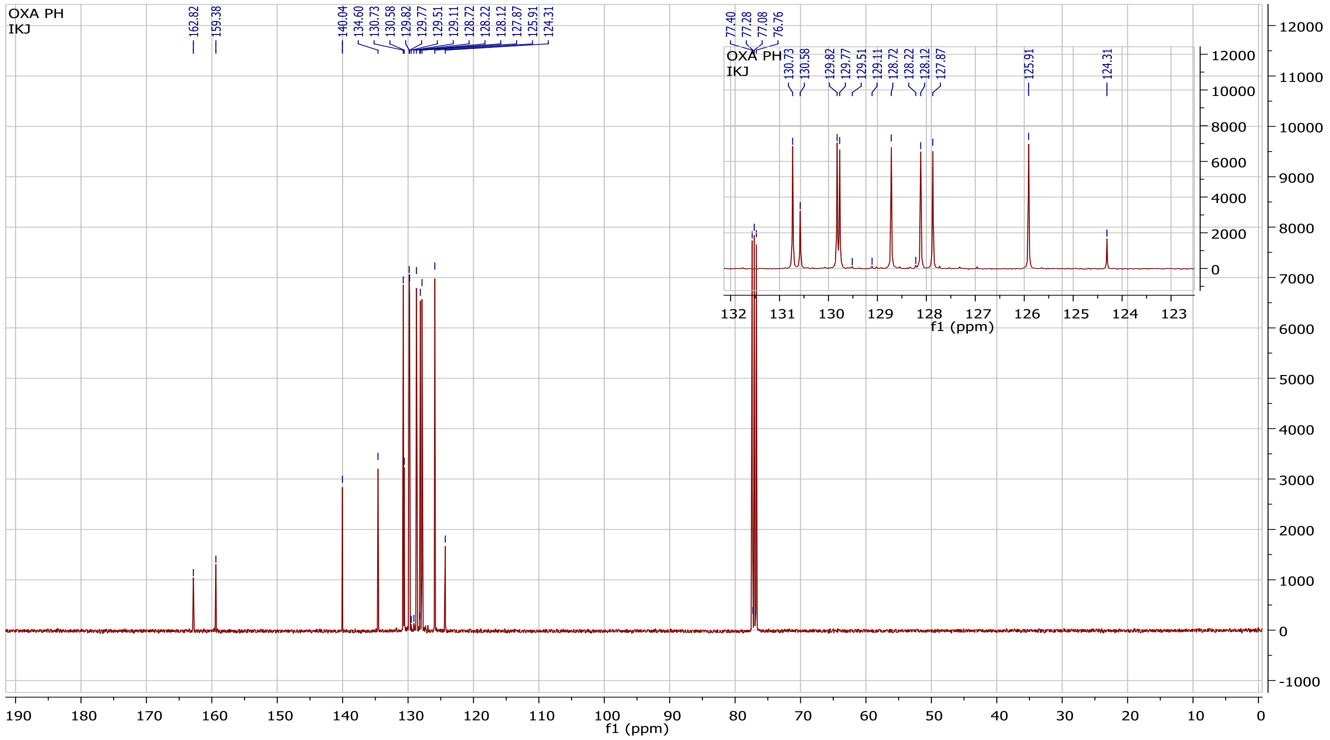

Figure S22: ^{13}C-NMR spectra of compound **5a**

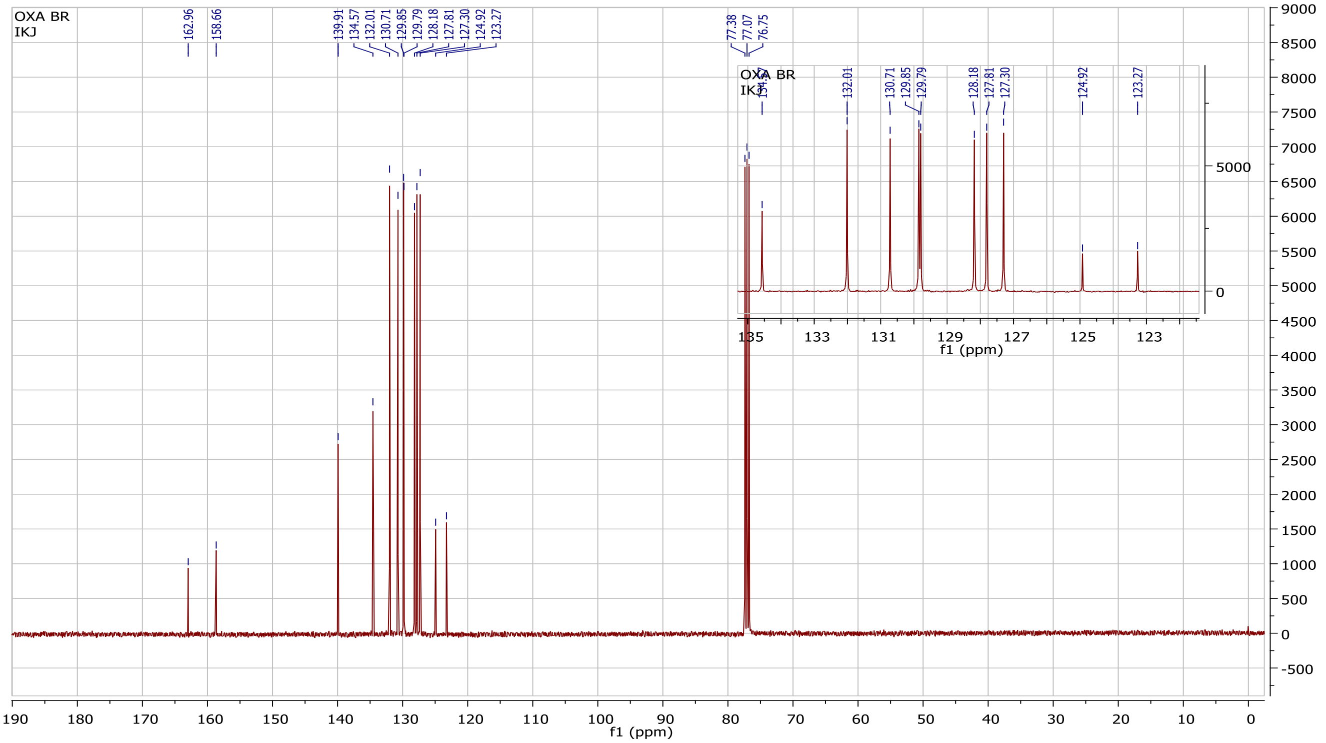

Figure S23: ^{13}C-NMR spectra of compound **5b**

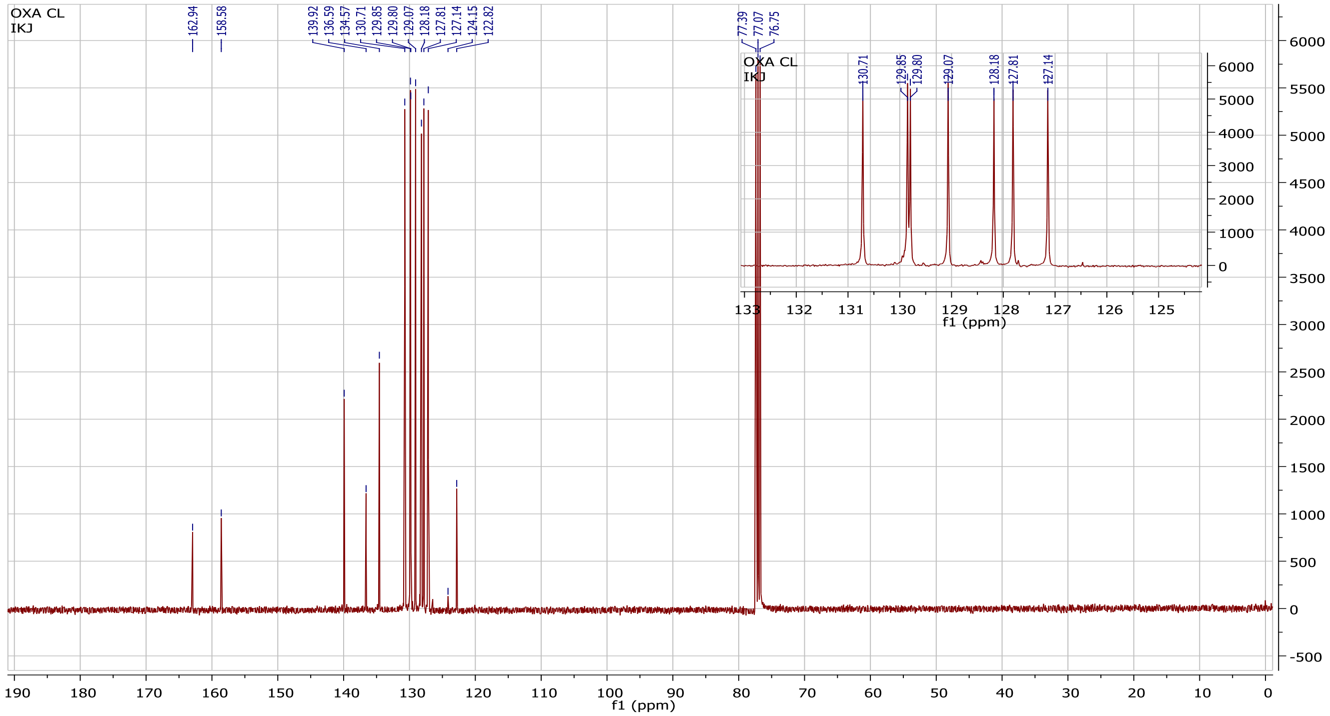

Figure S24: ^{13}C-NMR spectra of compound **5c**

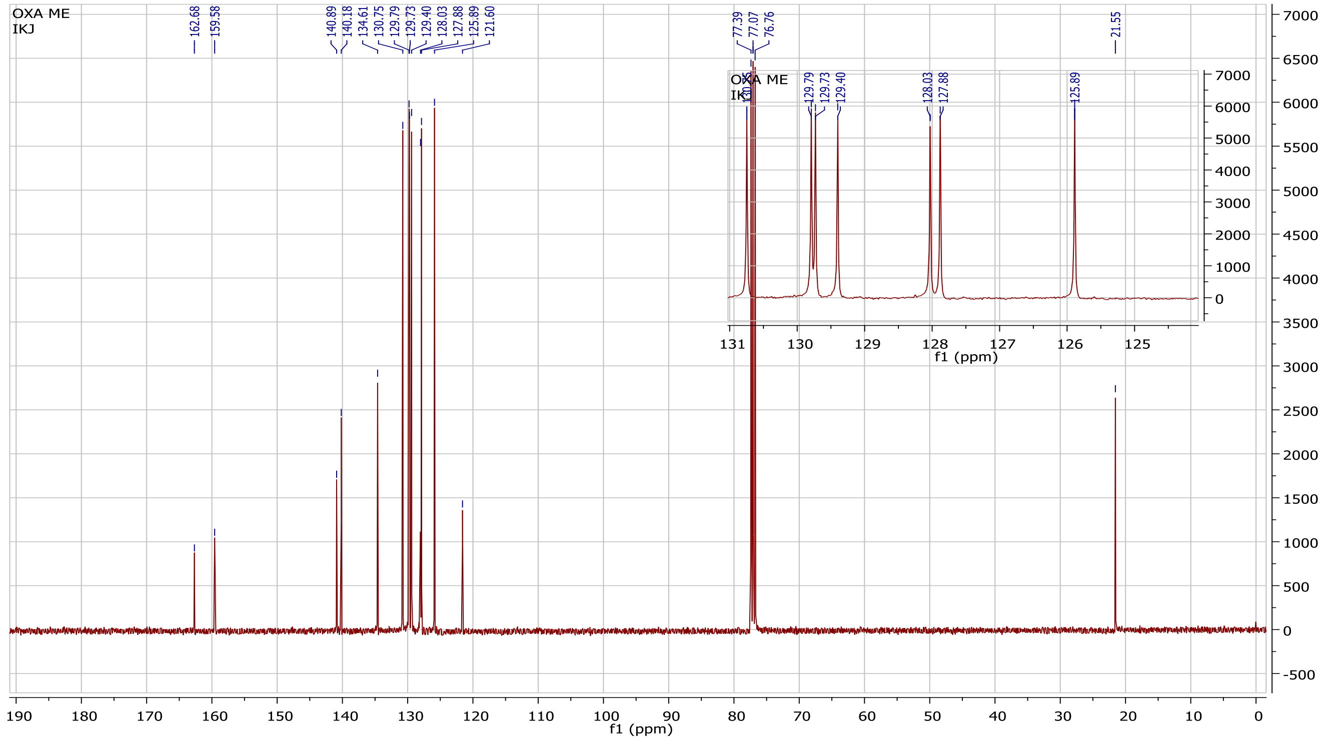

Figure S25: ^{13}C-NMR spectra of compound **5d**

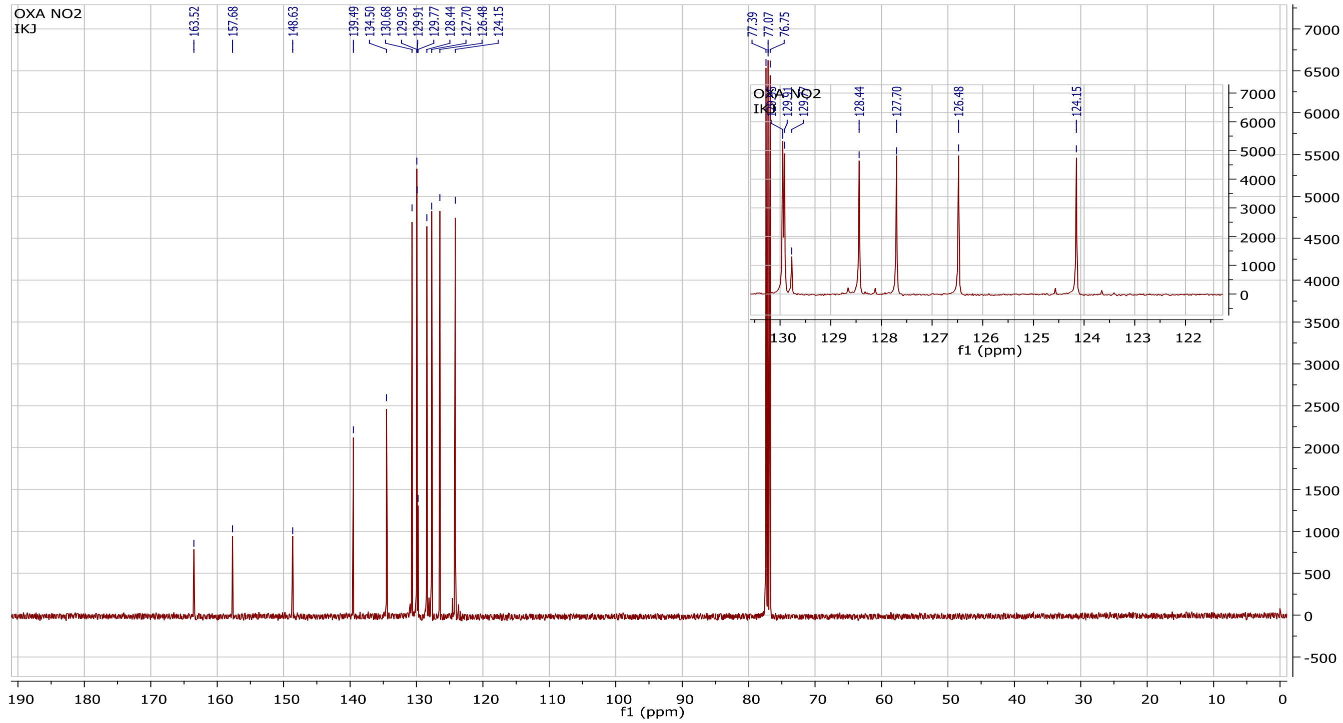

Figure S26: ^{13}C-NMR spectra of compound **5e**

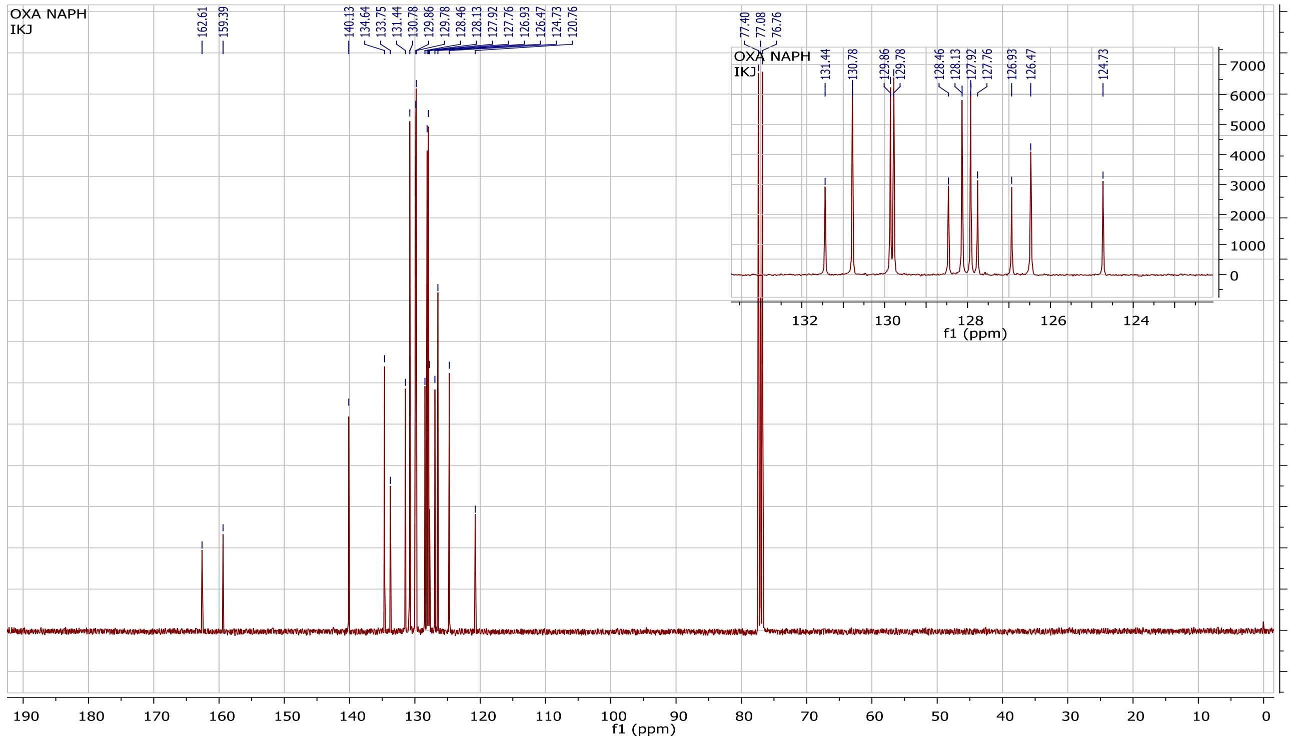

Figure S27: ^{13}C-NMR spectra of compound **5f**

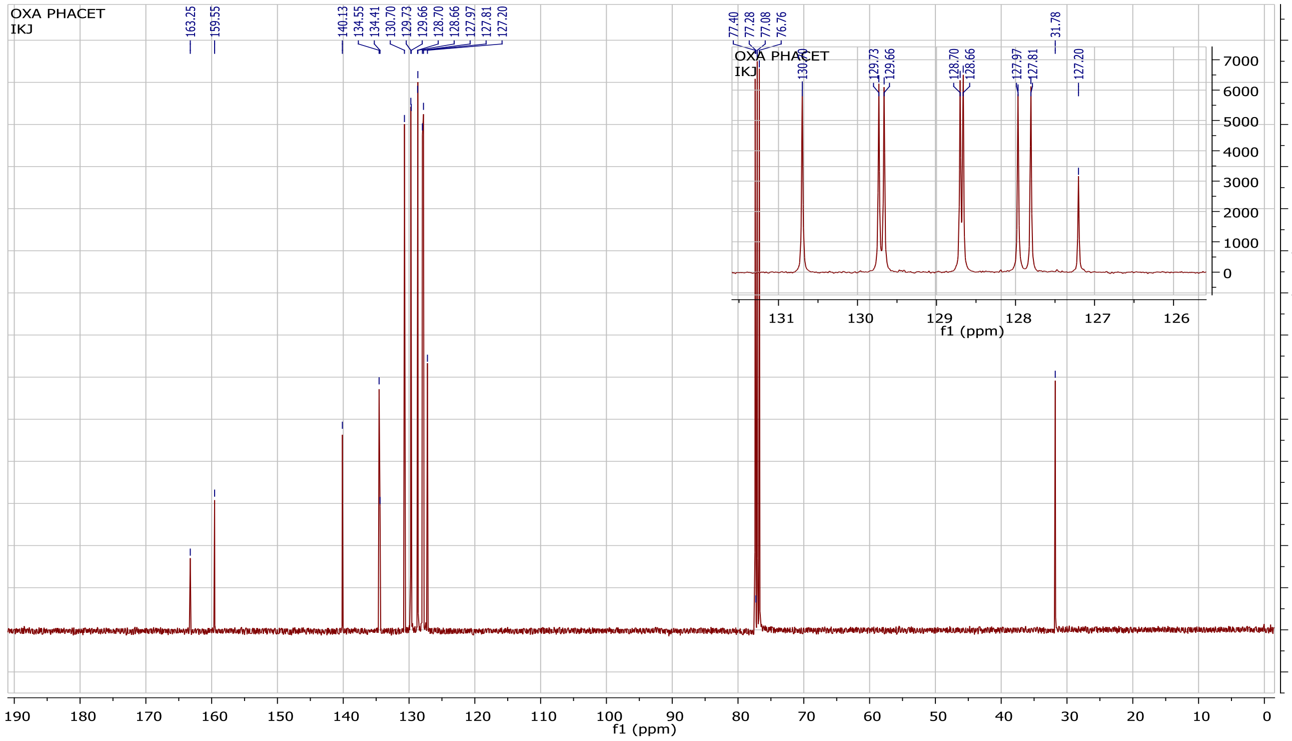

Figure S28: ^{13}C-NMR spectra of compound **5g**

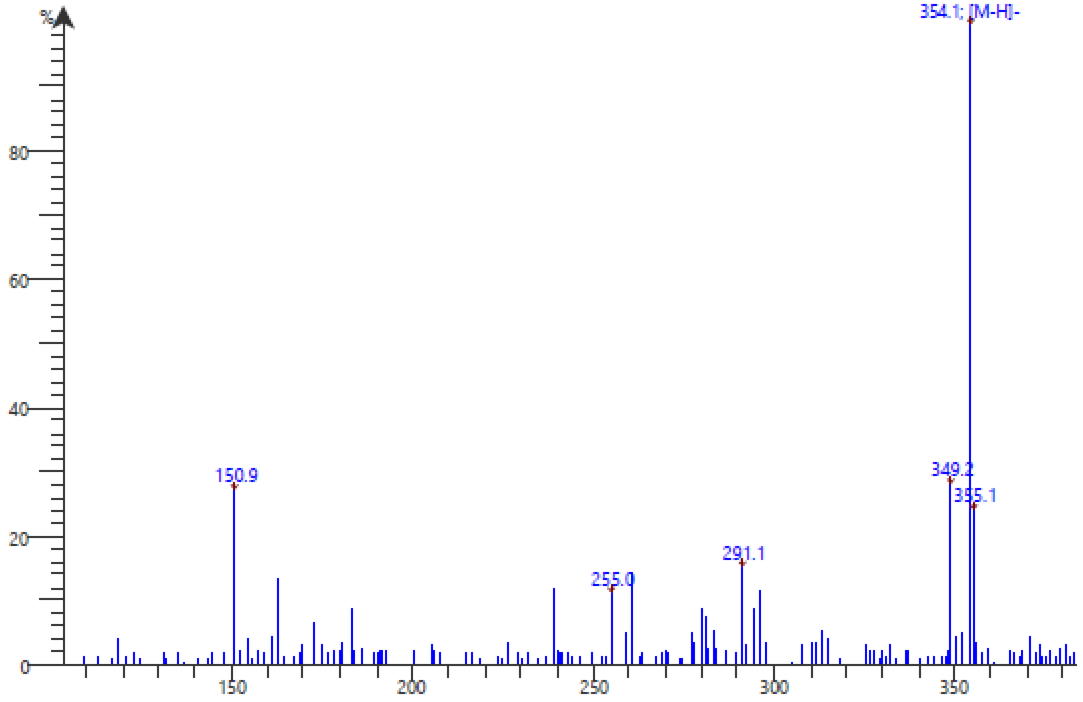

Figure S29: Mass spectra of compound **4a**

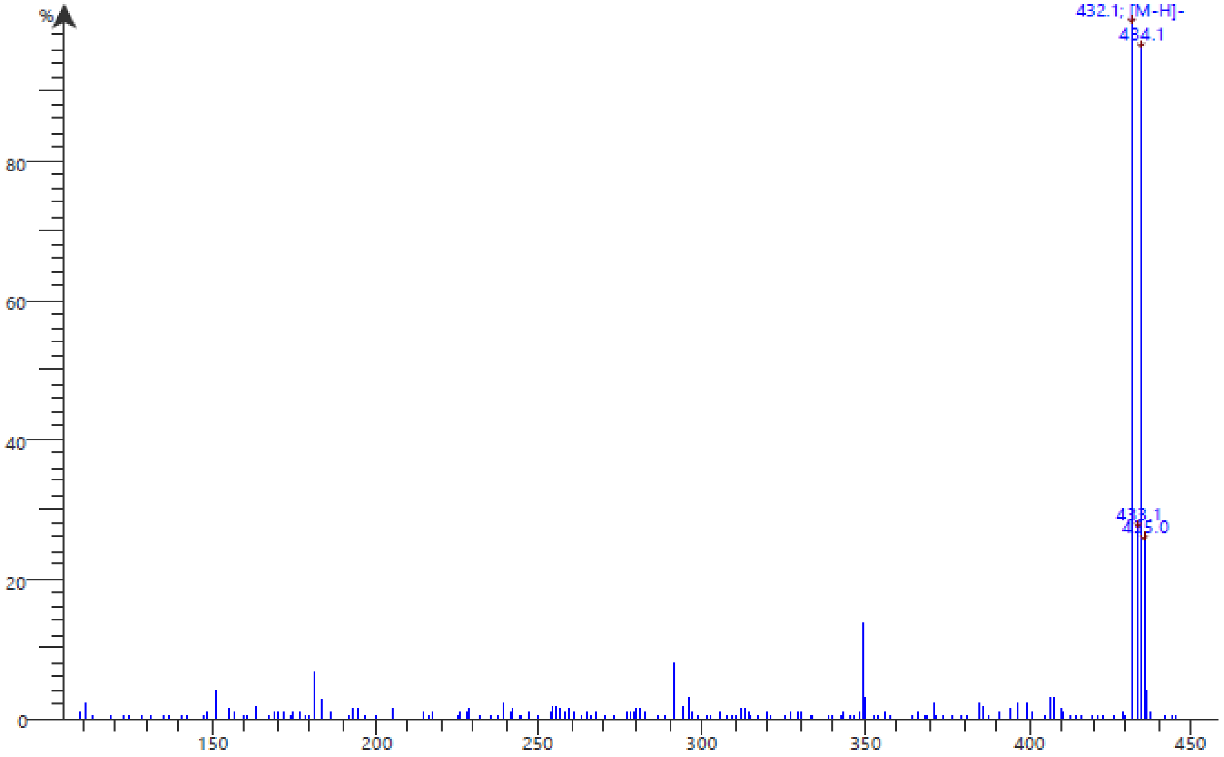

Figure S30: Mass spectra of compound **4b**

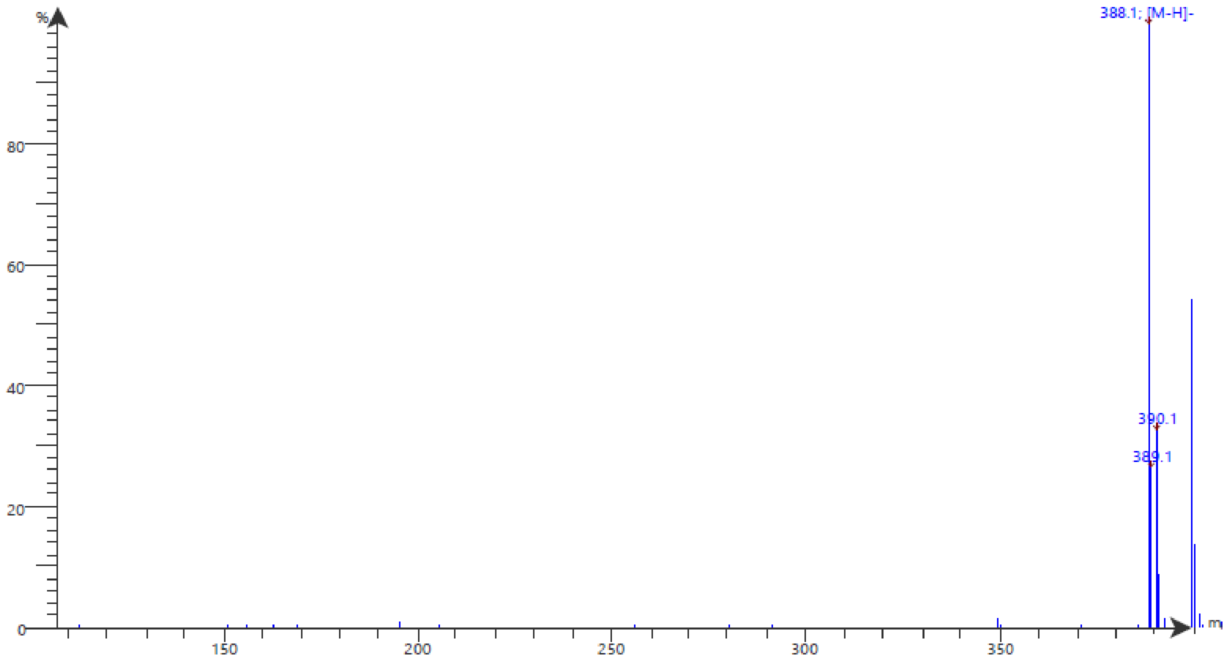

Figure S31: Mass spectra of compound **4c**

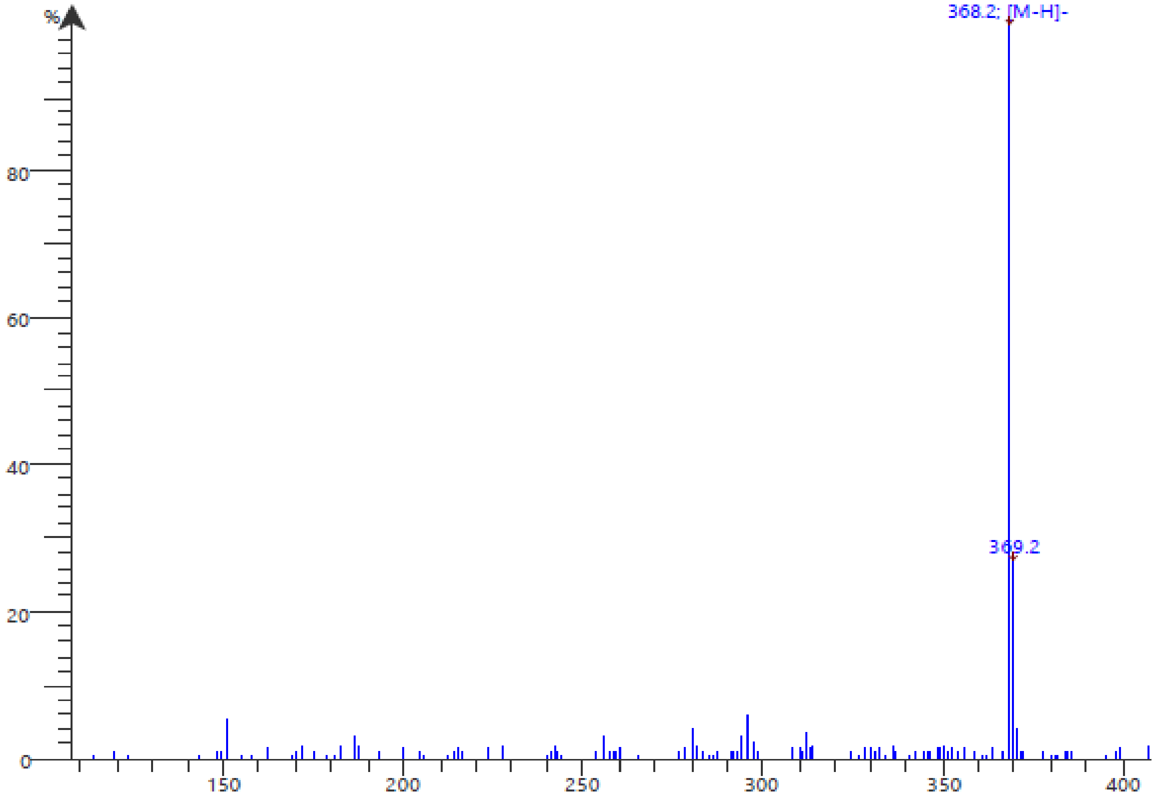

Figure S32: Mass spectra of compound **4d**

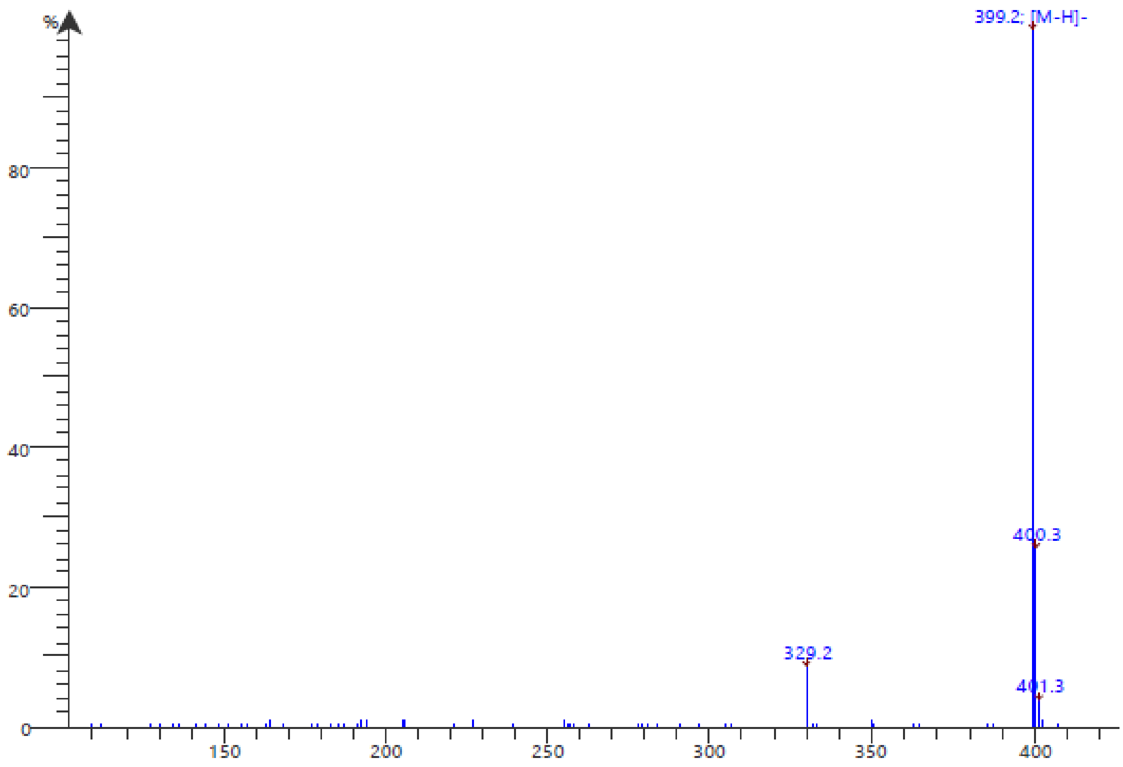

Figure S33: Mass spectra of compound **4e**

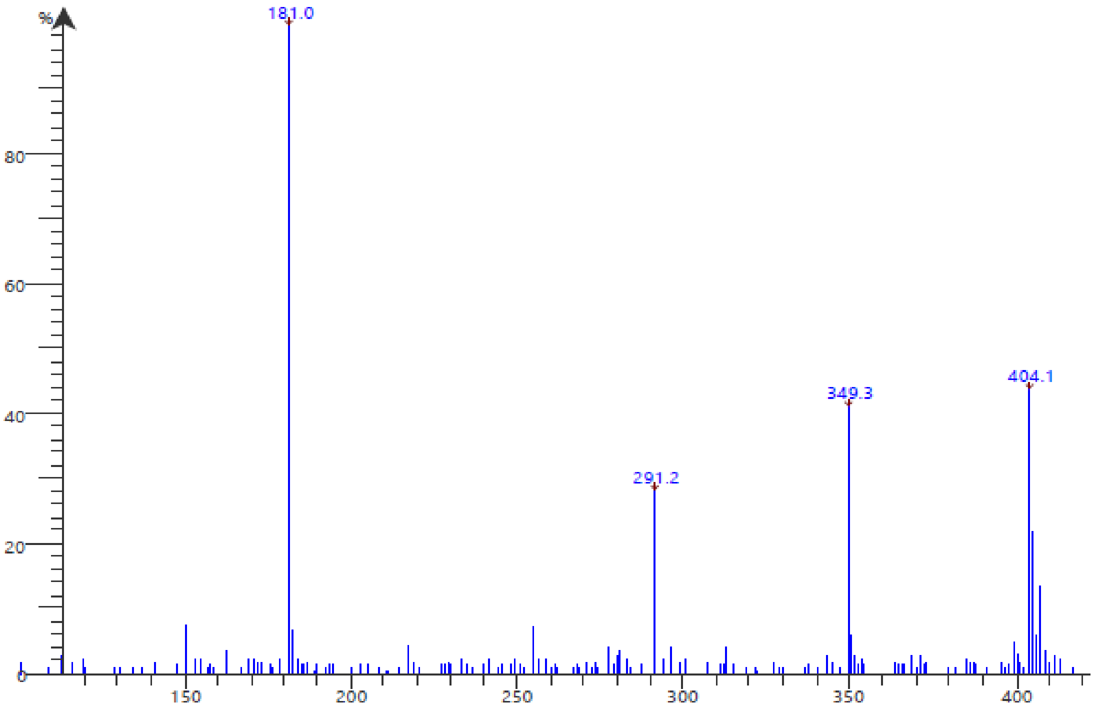

Figure S34: Mass spectra of compound **4f**

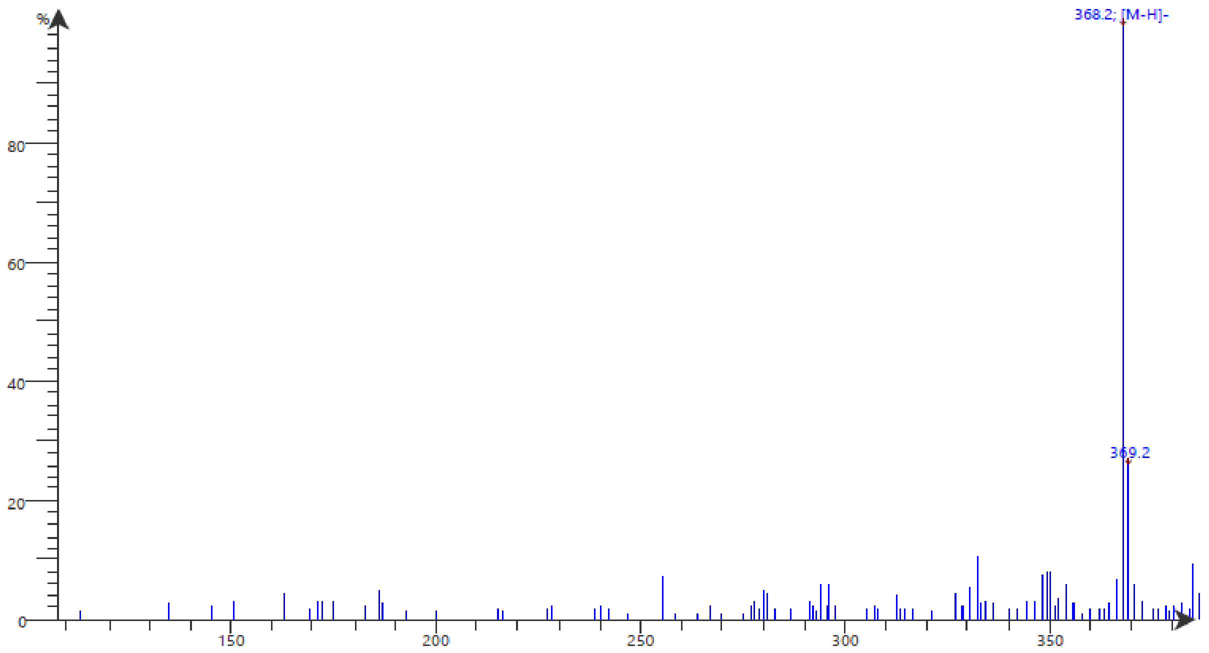

Figure S35: Mass spectra of compound **4g**

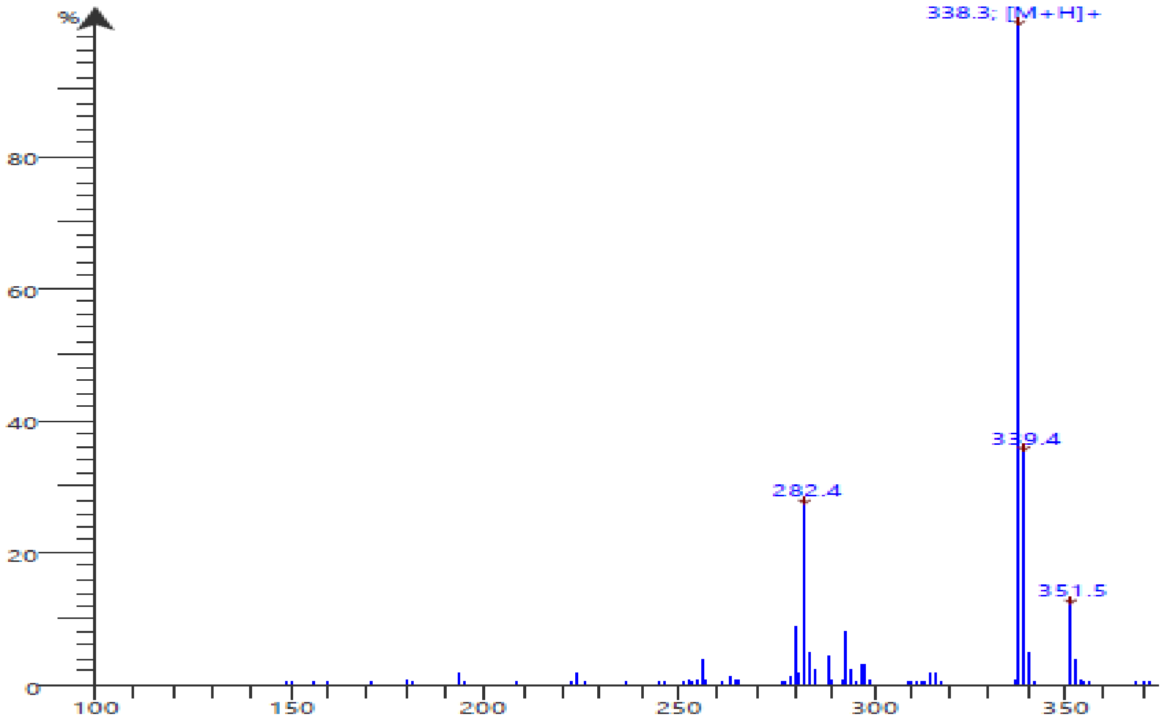

Figure S36: Mass spectra of compound **5a**

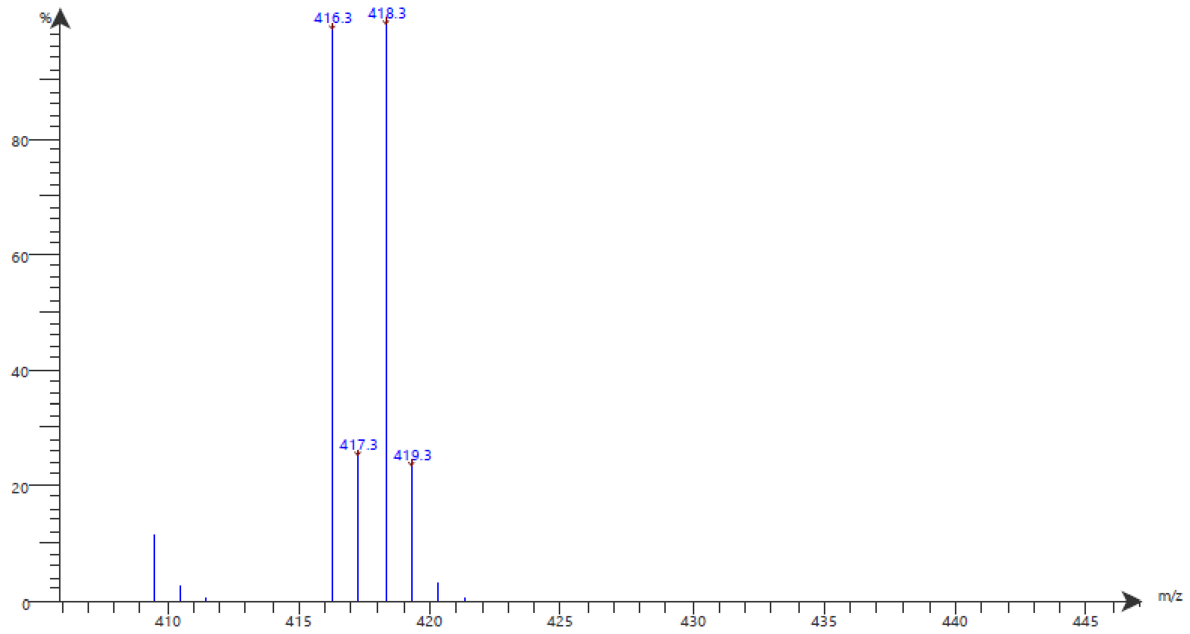

Figure S37: Mass spectra of compound **5b**

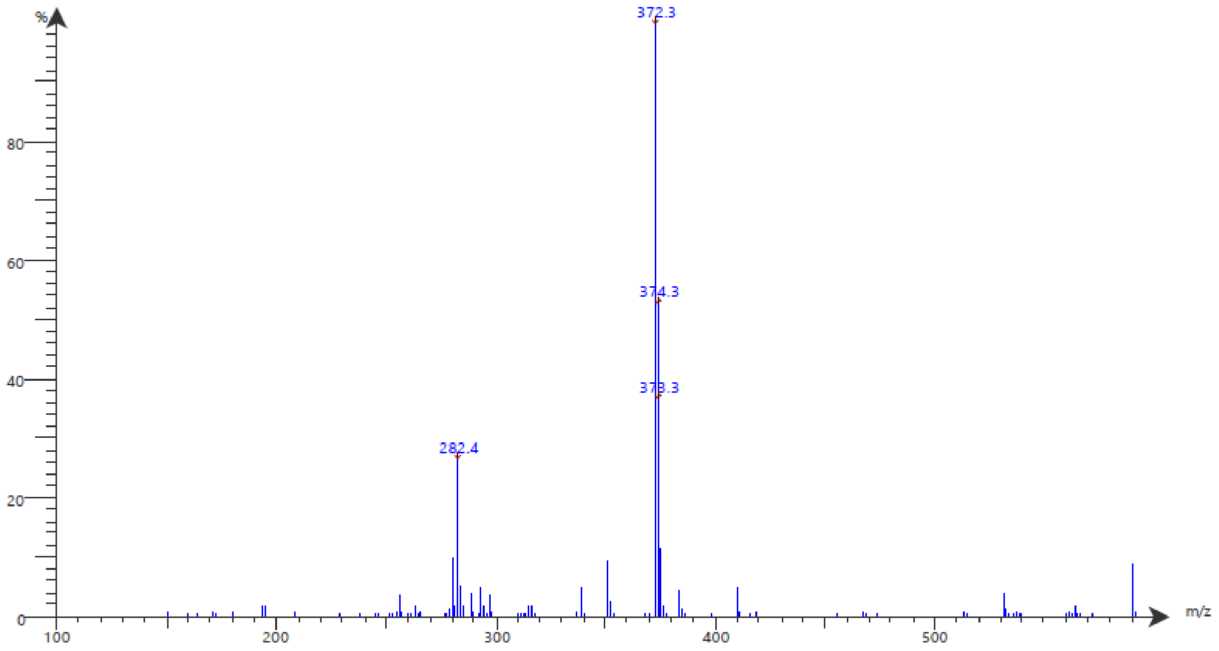

Figure S38: Mass spectra of compound **5c**

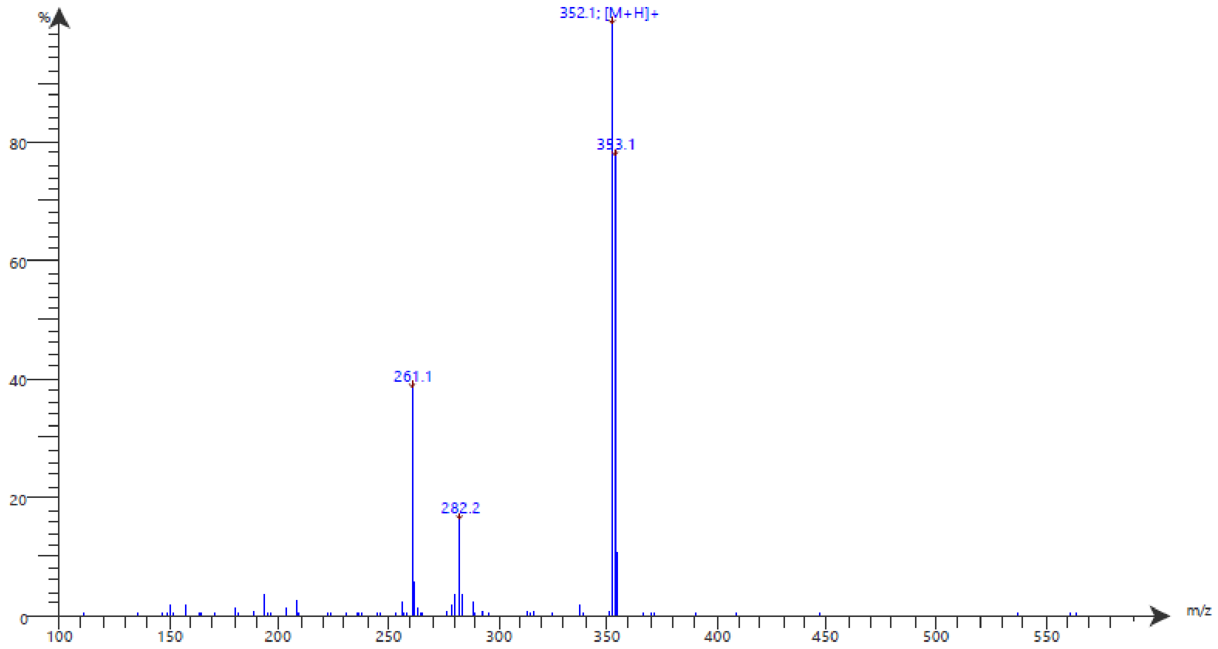

Figure S39: Mass spectra of compound **5d**

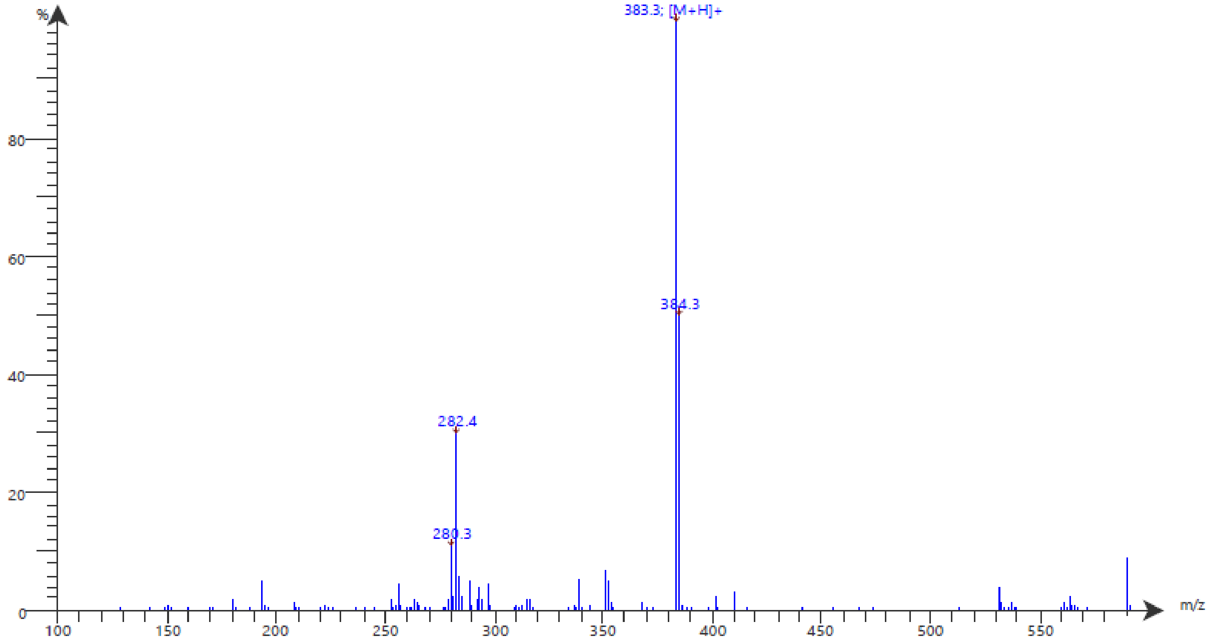

Figure S40: Mass spectra of compound **5e**

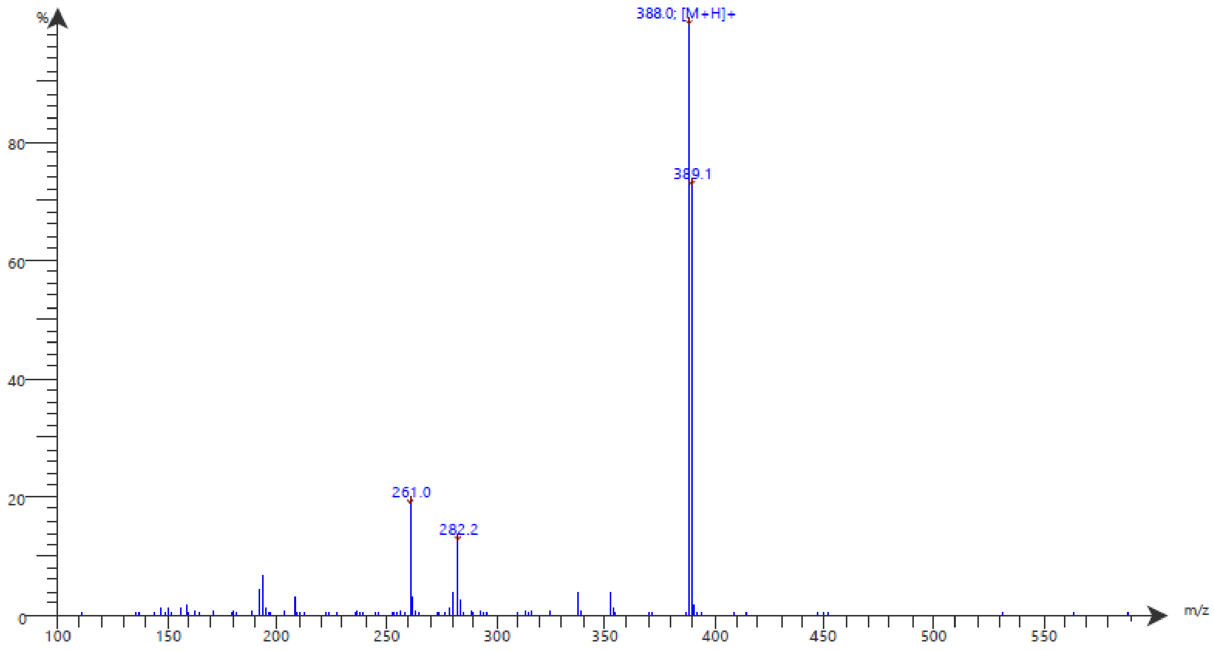

Figure S41: Mass spectra of compound **5f**

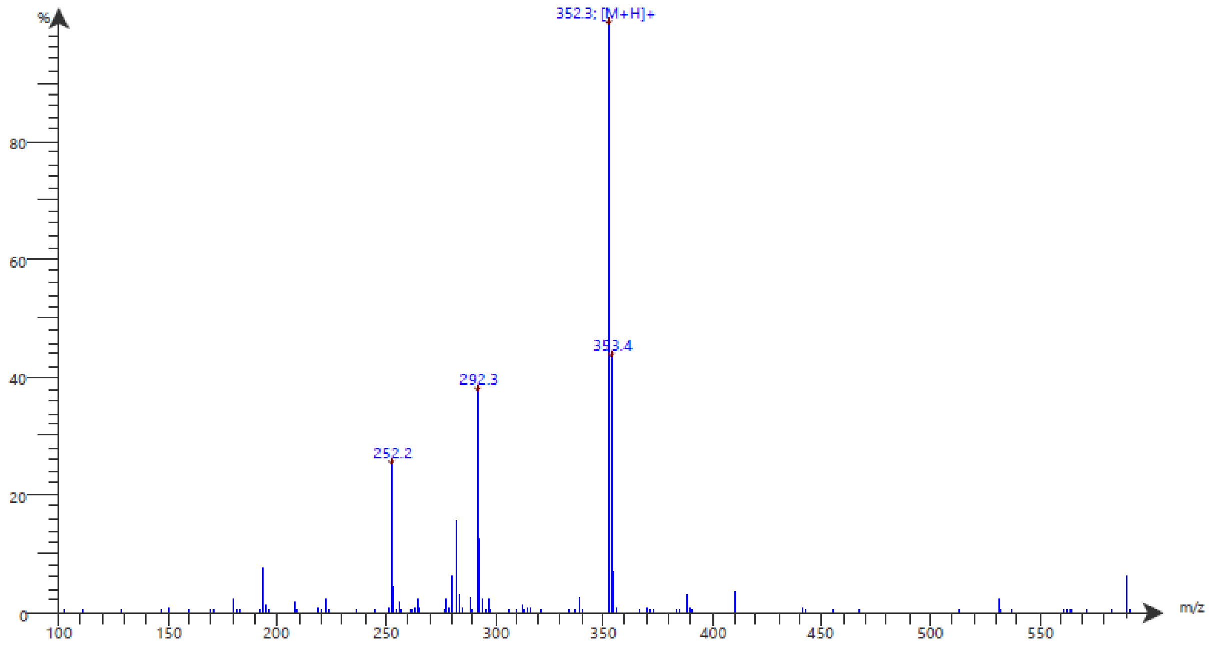

Figure S42: Mass spectra of compound **5g**

SI 3. Cell cycle analysis

Leukemia SR cells were grown in six-well plates (each one contains 2 x 105 cells per well) containing 10% fetal bovine serum and incubated for 24 h at 37ºC and 5% CO2. The medium was replaced with (DMSO 1% v/v) containing the 3.1 µM of compound **5e**, then incubated for 48 h, collected and washed with cold phosphate buffered saline (PBS). After fixation of the collected cells with ice-cold absolute ethanol (70%), the cells were rinsed with PBS then stained with the DNA fluorochrome PI, kept for 15 min at 37ºC. Then samples were analyzed with a FACS Caliber flow cytometer.

SI 4. Detection of apoptosis

Apoptotic activity of compound **5e** was evaluated using Annexin V-FITC/PI apoptosis detection kit. Leukemia SR cells (2 x 105) were seeded and incubated with compound **5e** for 48 h, trypsinized, collected and washed with phosphate-buffered saline (PBS) several times; SR cells were stained with Annexin V fluorescein isothiocyanate (FITC) and counterstained with propidium iodide (PI) for 15 min at 37ºC in the dark using the apoptosis detection kit (BD Biosciences, San Jose, CA) according to the manufacturer's protocol. Then, Annexin V-FITC and PI binding were analysed by a FACS Caliber flow cytometer.

SI 5. *In vivo* assay

SI 5.1. Animals and tumor cell line

Adult female Swiss albino mice purchased from Theodor Bilharzia Research Institute, Giza, Egypt, with an average body weight of (18-23) g was used. Mice were housed under constant conditions of 12 h light/dark cycle in a temperature under conditions of controlled humidity (22 ± 2 °C), with free access to standard laboratory mice food and water.

All procedures related to care and maintenance of the animals were performed according to the international guiding principles for animal research and approved by Faculty of Science, Suez Canal University bioethics and animal ethics committee (Approval number REC-01-2022).

Solid Ehrlich carcinoma (SEC) were got from the National Cancer Institute (Cairo University, Egypt). The tumor cell line was proliferated in mice through serial intraperitoneal (I.P.) transplantation of a volume of 0.2 mL physiological saline contains 1×10^6 viable cells for 24 h. SEC cells were collected 7 days after I.P. implantation. The harvested cells were diluted with saline to obtain a concentration of 5×10^6 viable SEC cells/mL. A volume of 0.2 mL saline contains 1×10^6 SEC cells

that were I.P. implanted into each normal mouse. SEC cells (1×10^6 tumor cells/mouse) were implanted subcutaneously into the right thigh of the hind limb.

The experimental animals were randomly divided into four groups. Group 1 served as the normal saline control. Group 2 served as the SEC control (1×10^6 cells/mouse). Group 3 served as the compound-treated group (6 mg/kg B.Wt., I.P.). Group 4 received the standard anticancer drug of Doxorubicin (DOX) (6 mg/kg BW, I.P.) and is considered as a reference control. Body weight and survival were recorded daily until the 24th day in both treated and control groups. At the end of experiment, the blood of each group was collected under light anesthesia for estimation of hematological and biochemical assays. The anesthetized animals were then sacrificed for evaluation of the antitumor activity and histopathological examination.

SI 5.2. Antitumor potentiality

It includes tumor volume, weight, and tumor inhibition ration (TIR%). Time interval measurements of tumor volume using digital Vernier caliper (Tricle Brand, Shanghai, China). Measure tumor length and width using clipper and then calculate tumor volume using formulations V = (L x W x W)/2, where V is tumor volume, W is tumor width, L is tumor length. While TIR% was calculated according to the following equation: $\frac{Tumor\ volume\ (Control) - Tumor\ volume\ (treated)}{Tumor\ volume\ (control)} x\ 100$.

SI 5.3. Blood assays

At the end of the experiment, animals from different groups were sacrificed, and blood samples were collected for determination of liver enzymes ALT, AST levels. Activities of aspartate aminotransferase (AST) and alanine aminotransferase (ALT) were evaluated using commercial kits (ELITech clinical systems, France). Serum albumin level was determined by kit purchased from STANBIO Company (USA).

SI 5.4. Histopathological study

Specimens of liver-sacrificed mice were fixed in 10% saline formalin. The fixed liver specimens were dehydrated in ascending series of ethyl alcohol and embedded in paraffin. Sections at 5 mm thicknesses were stained with hematoxylin and eosin and examined under light microscopy.

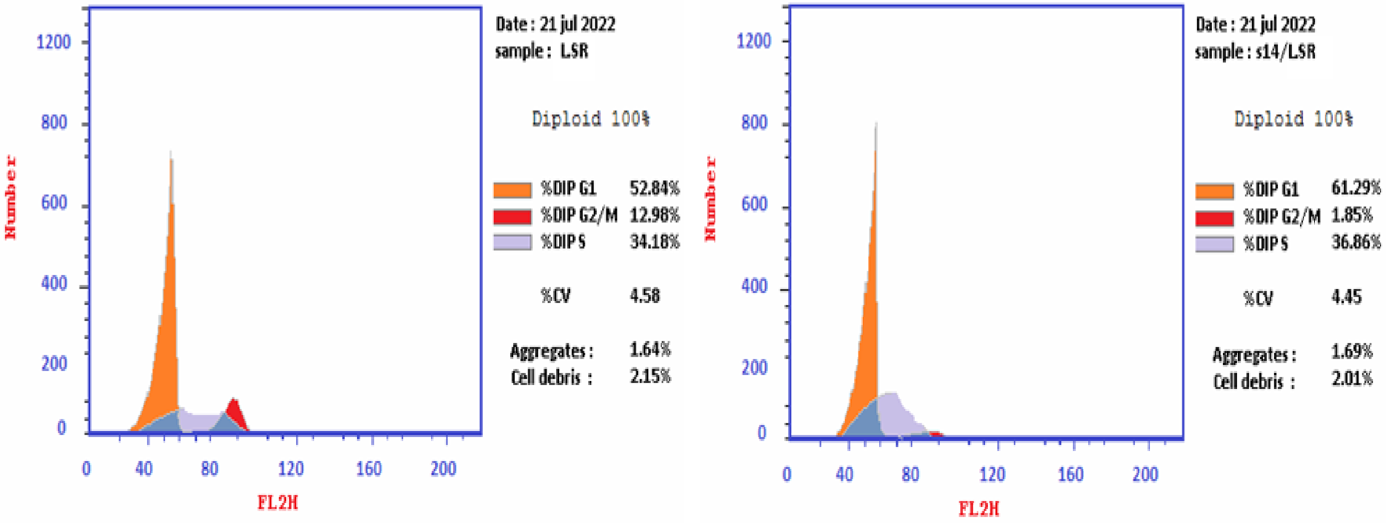

Figure SI 1. Cell cycle histograms of SR cells treated with compound **5e**. "Q1: Necrosis, Q2: Late apoptosis, and Q4: Early apoptosis".

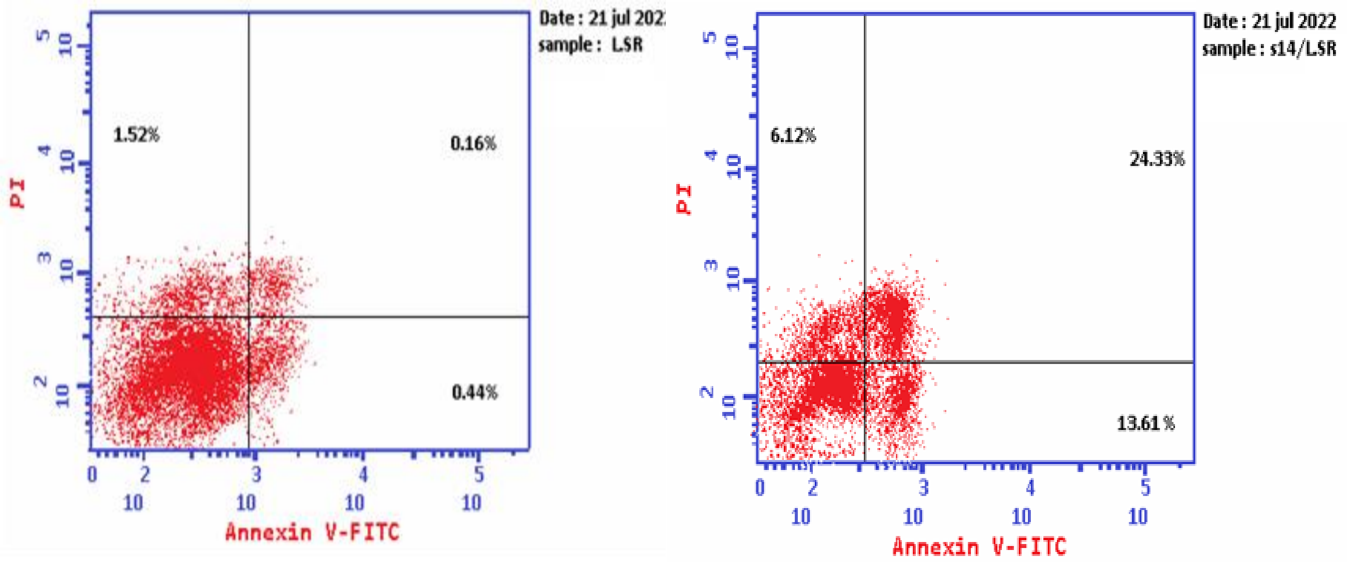

Figure SI 2. Annexin V/PI staining histograms of compound **5e** in SR cells.

Table SI 1. Docking binding scores (-CDOCKER INTERACTION ENERGY) for the compounds **4a-g** and **5a-g** in the active site of DNA-Topo II complex and the reference compound doxorubicin (PDB code: 3QX3).

Compound	-CDOCKER INTERACTION ENERGY (kcal/mol)
**4a**	-35.90
**4b**	-33.00
**4c**	-34.30
**4d**	-33.60
**4e**	-35.50
**4f**	-34.20
**4g**	-35.20
**5a**	-37.20
**5b**	-35.80
**5c**	-35.00
**5d**	-35.00
**5e**	-39.60
**5f**	-38.20
**5g**	-39.00
**Doxorubicin**	-48.30

Table SI 2. 2 D and 3 D interactions for compounds (**4a-g** and **5a-g**) at the DNA–topo II complex active site (PDB code 3QX3).

Compd.	2 D interaction	3 D interaction
**4a**	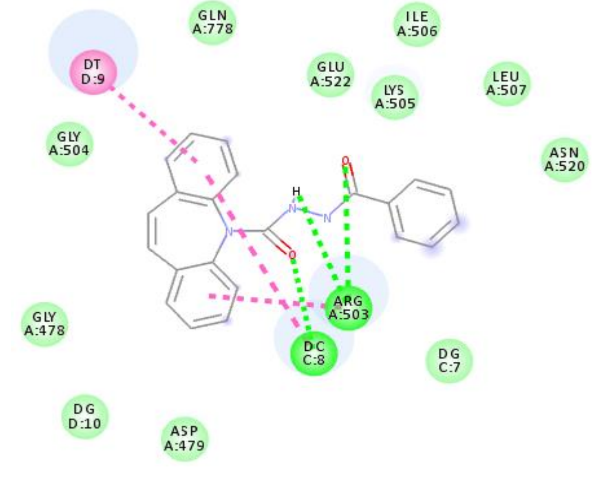	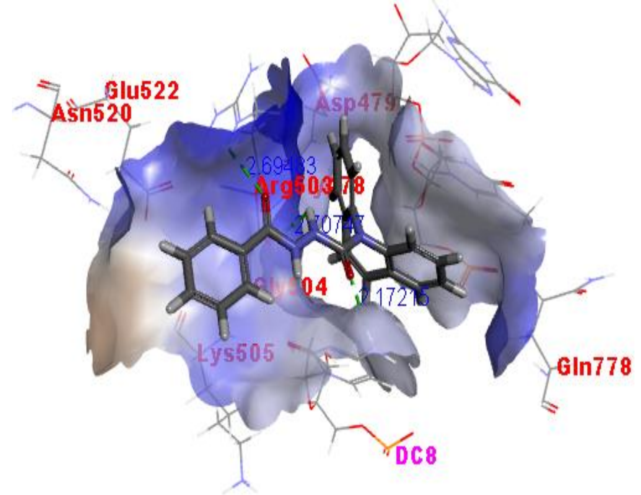
**4b**	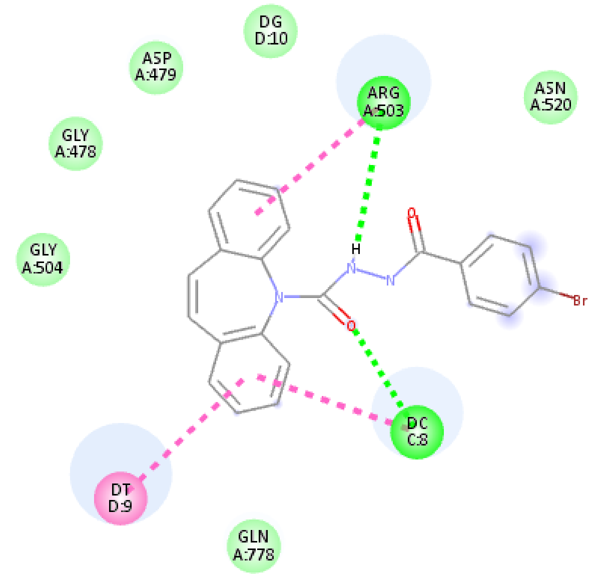	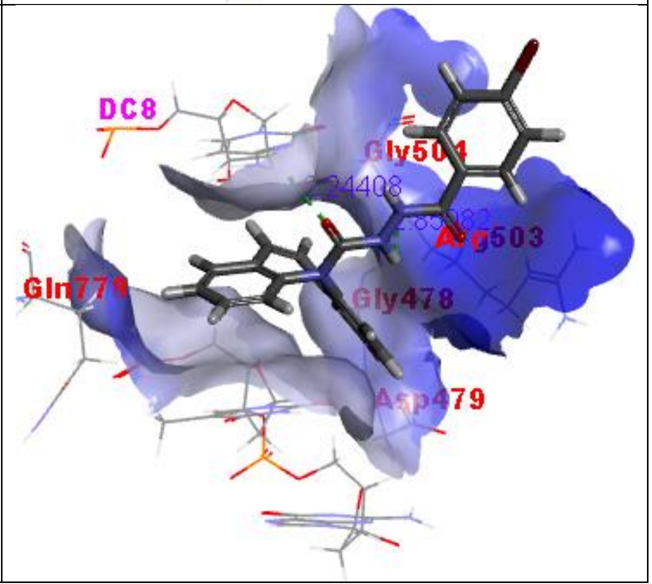

<table>
<tr><td>4c</td><td>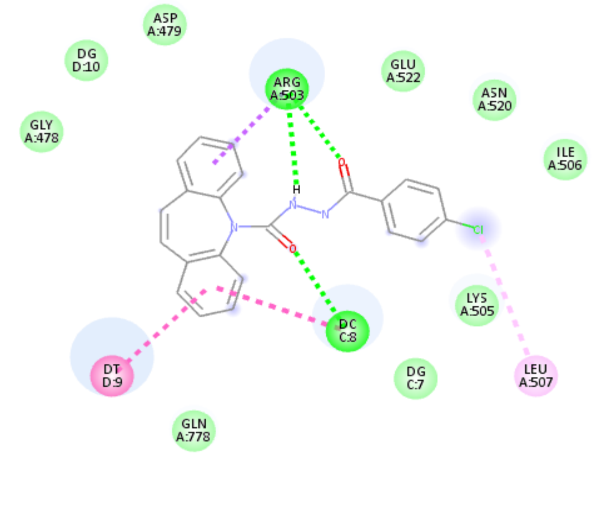</td><td>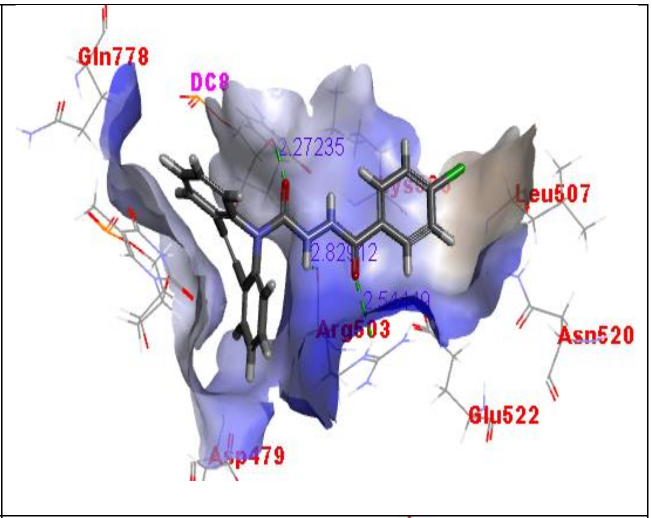</td></tr>
<tr><td>4d</td><td>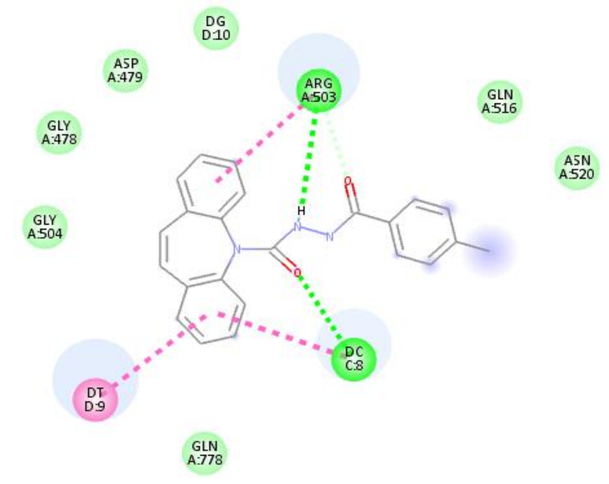</td><td>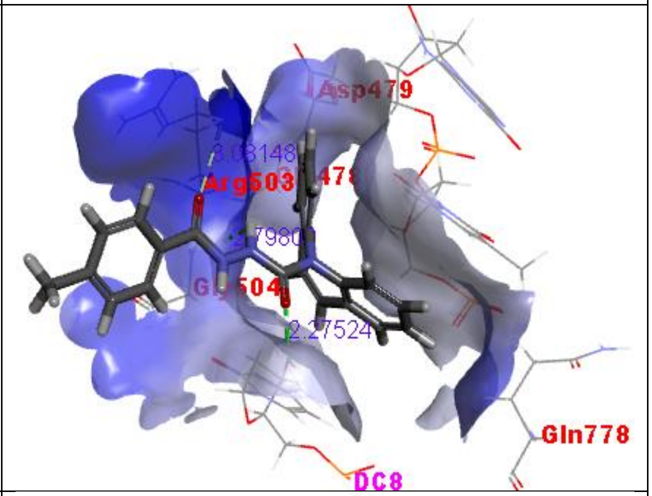</td></tr>
<tr><td>4e</td><td>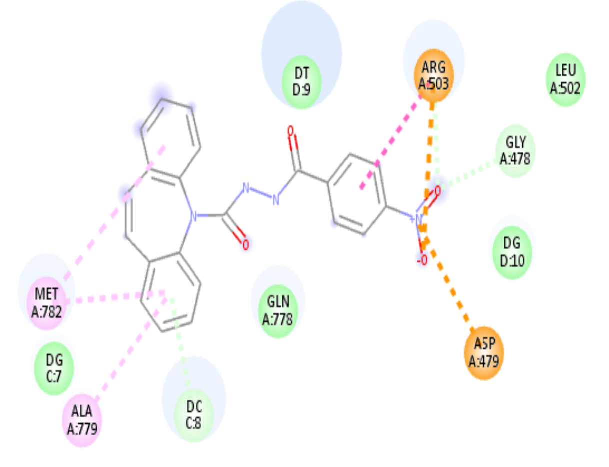</td><td>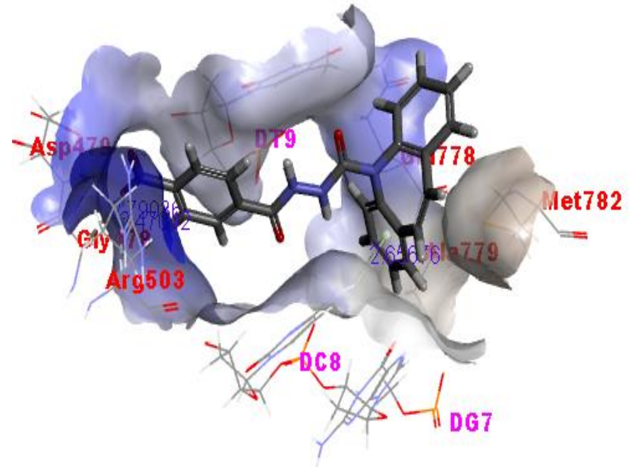</td></tr>
</table>

<table>
<tr><td>4f</td><td>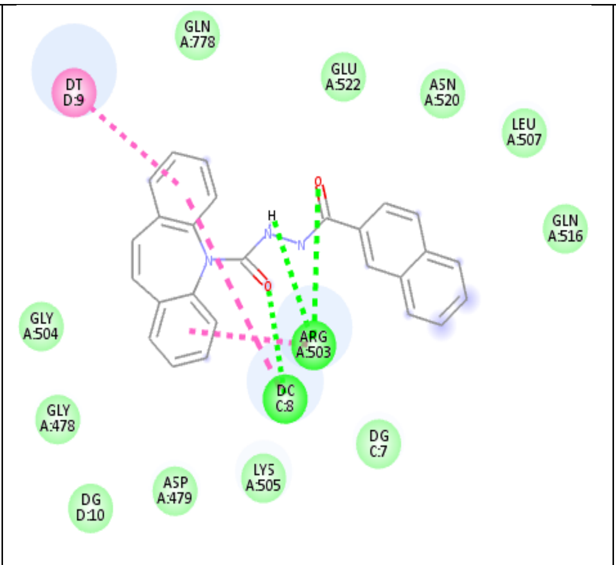
</td><td>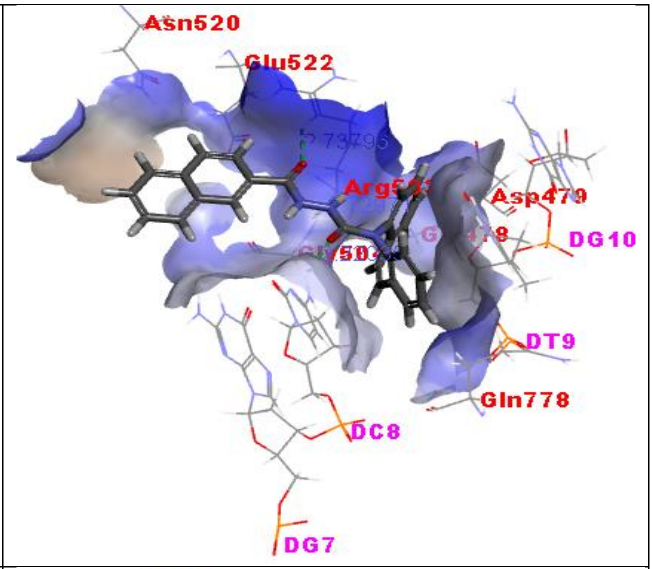
</td></tr>
<tr><td>4g</td><td>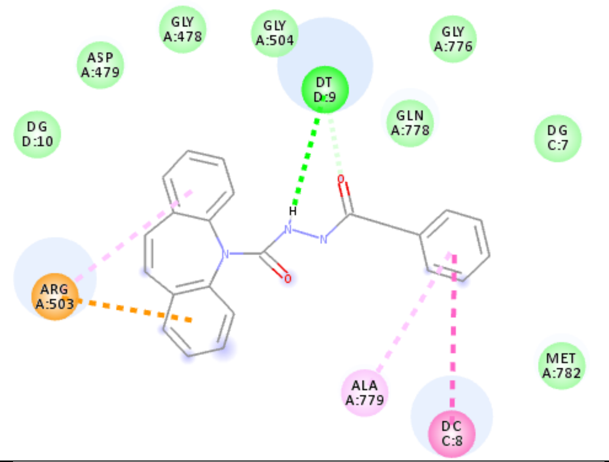
</td><td>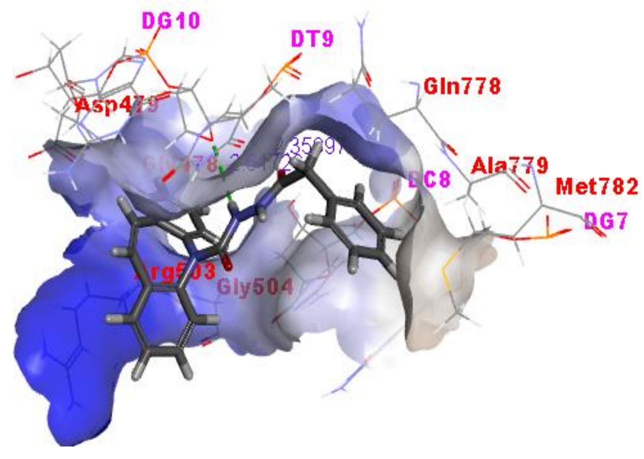
</td></tr>
<tr><td>5a</td><td>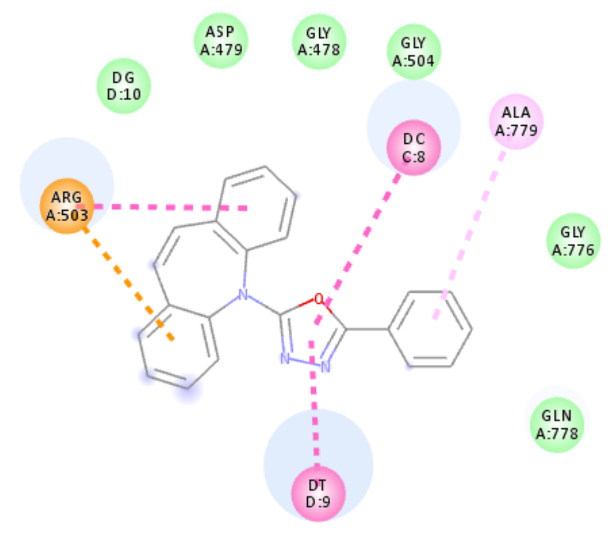
</td><td>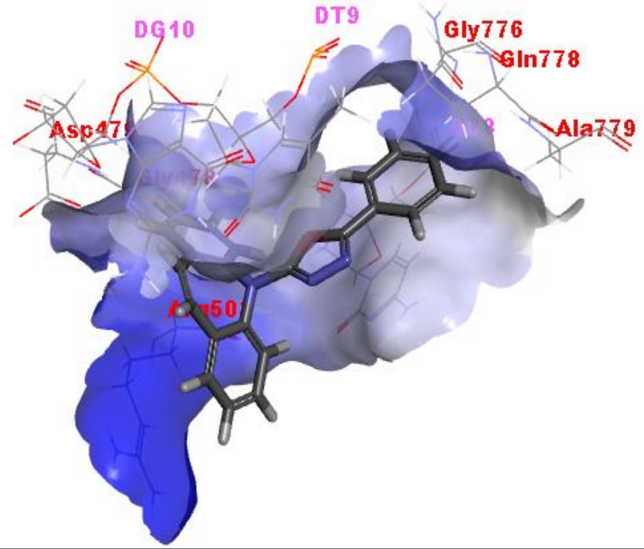
</td></tr>
</table>

5b

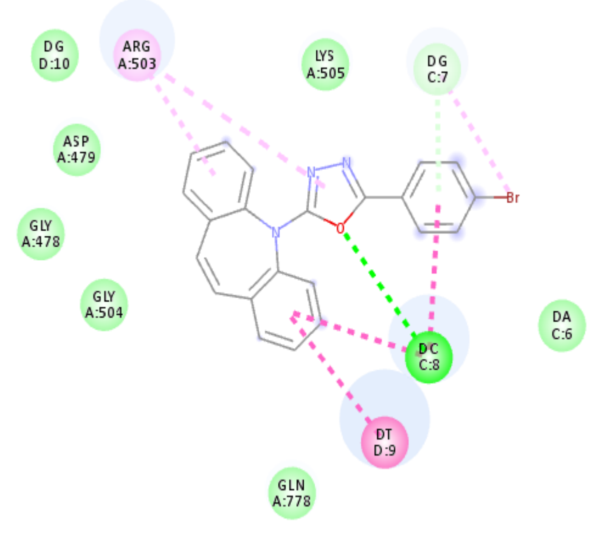
DG D:10
ARG A:503
LYS A:505
DG C:7
ASP A:479
GLY A:478
GLY A:504
DA C:6
DC C:8
DT D:9
GLN A:778
Br

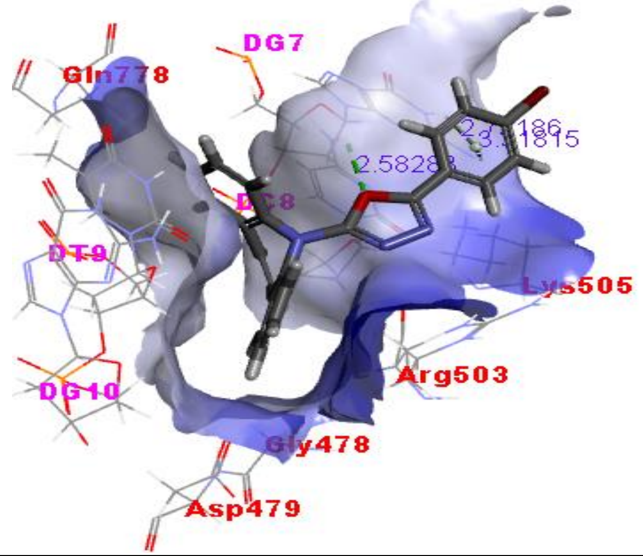
DG7
Gln778
2.5828
DC8
DT9
Lys505
Arg503
DG10
Gly478
Asp479

5c

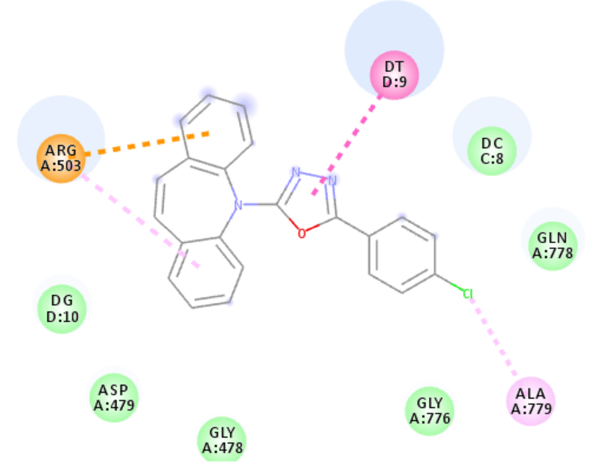
DT D:9
ARG A:503
DC C:8
GLN A:778
DG D:10
Cl
ASP A:479
GLY A:776
ALA A:779
GLY A:478

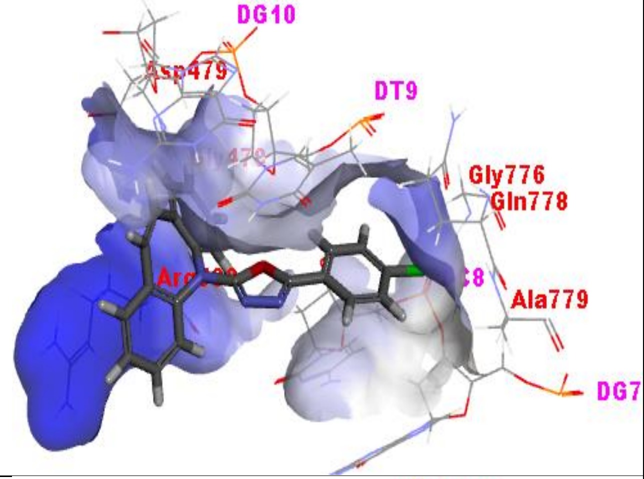
DG10
Asp479
DT9
Gly776
Gln778
Ala779
DG7

5d

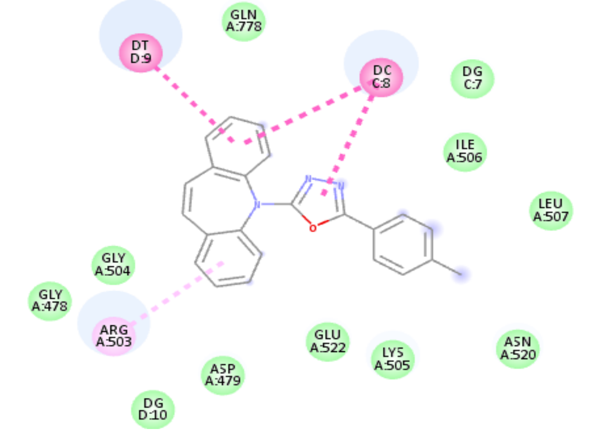
DT D:9
GLN A:778
DC C:8
DG C:7
ILE A:506
LEU A:507
GLY A:504
GLY A:478
ARG A:503
GLU A:522
LYS A:505
ASN A:520
ASP A:479
DG D:10

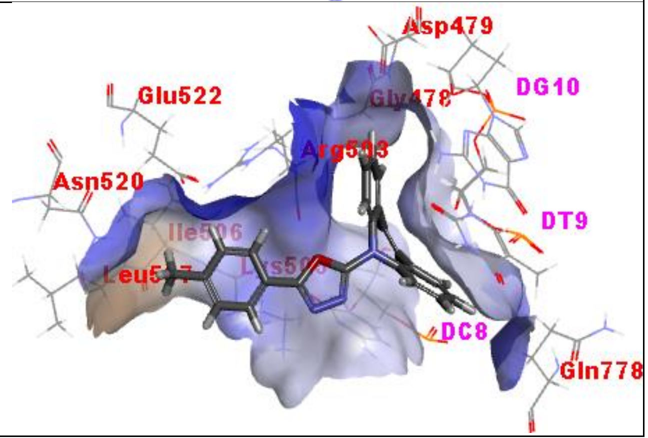
Asp479
Glu522
Gly478
DG10
Arg503
Asn520
Ile506
DT9
DC8
Gln778

<table>
<tr><td>5e</td><td>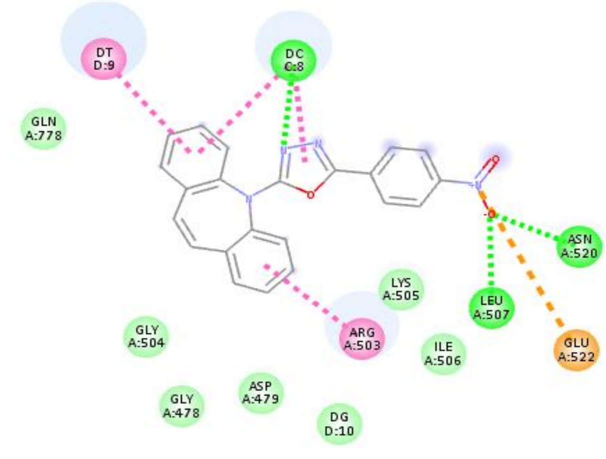
</td><td>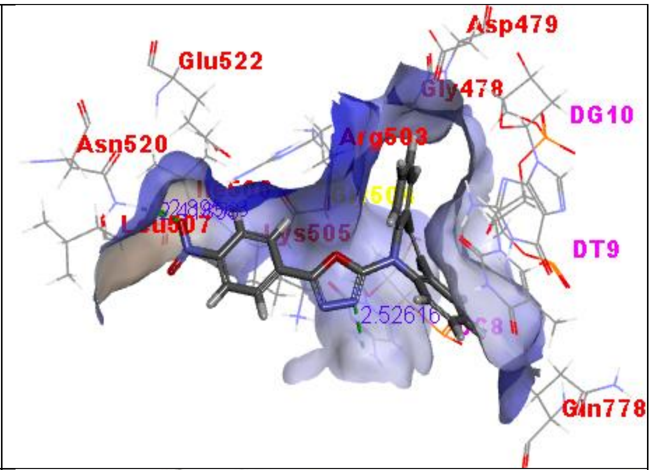
</td></tr>
<tr><td>5f</td><td>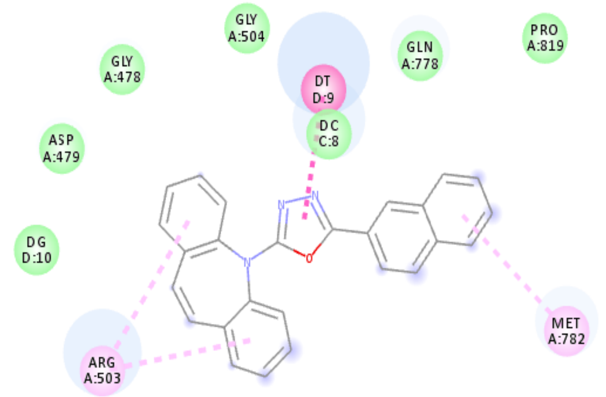
</td><td>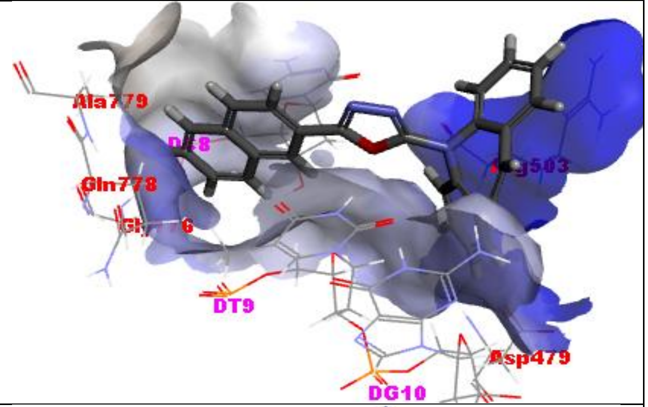
</td></tr>
<tr><td>5g</td><td>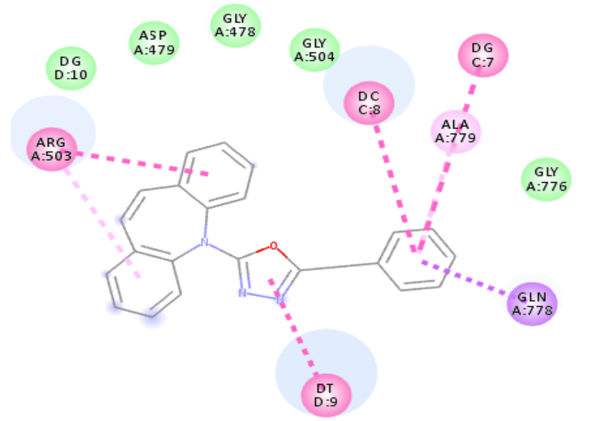
</td><td>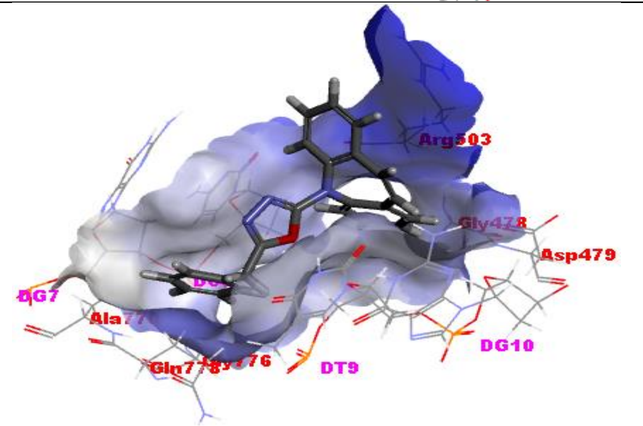
</td></tr>
<tr><td>Co-crystallize ligand (EVP)</td><td>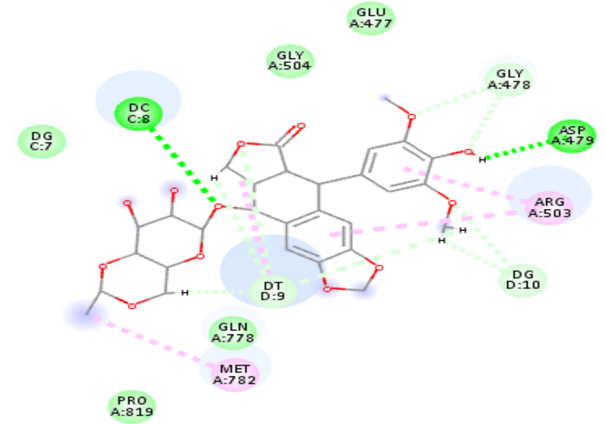
</td><td>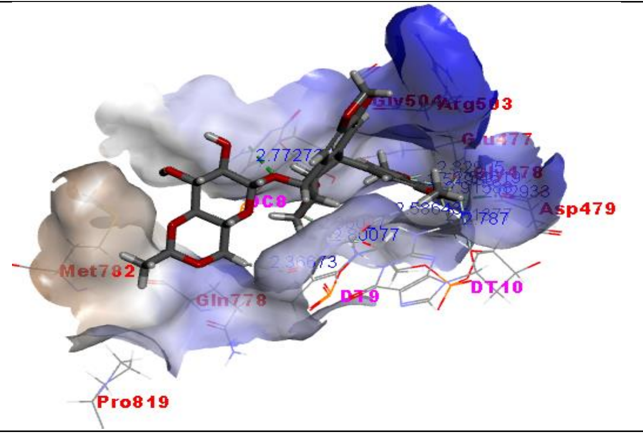
</td></tr>
</table>

**Doxorubicin**	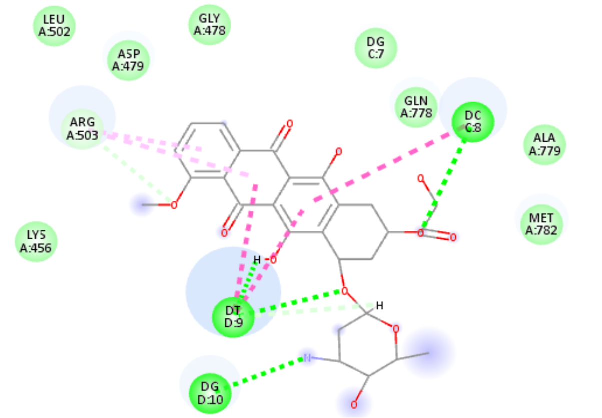	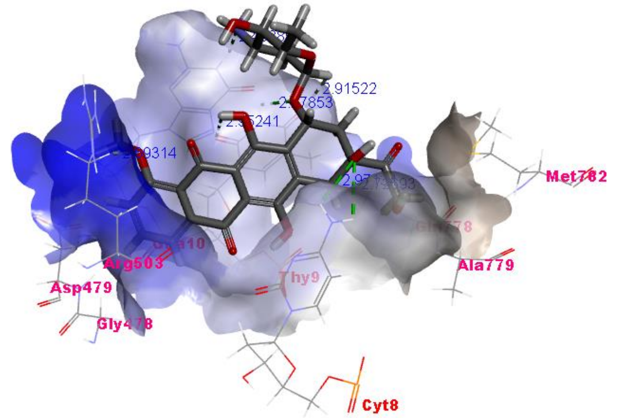

Table SI 3. Physicochemical parameters of the synthesized compounds **4a-g**.

Comp.	M.wt	NRB	HBA	HBD	TPSA (Å^2)	Lipiniski	Log p	Water solubility	MR
**4a**	389.83	5	2	2	61.44	Yes, 1 violation	4.01	MS	113.67
**4b**	369.42	5	2	2	61.44	Yes, 1 violation	3.67	MS	113.62
**4c**	400.39	6	4	2	107.26	Yes, 0 violation	2.90	MS	117.48
**4d**	405.45	5	2	2	61.44	Yes, 1 violation	4.40	PS	126.16
**4e**	369.42	6	2	2	61.44	Yes, 0 violation	3.40	MS	113.24
**4f**	337.37	2	3	0	42.16	Yes, 1 violation	4.48	MS	106.28
**4g**	416.27	2	3	0	42.16	Yes, 1 violation	5.17	PS	113.98

Num. of rotable bond (NRB); Num. H. bond donor (HBD); Num. H. bond Acceptor (HBA); Topological polar surface area (TPSA); Molar Refractivity (MR).

Table SI 4. Pharmacokinetic parameters and ADMET study of the synthesized compounds **4a-g**.

Comp.	ADMET Parameters					PAINS	Synthetic accessibility
	Log K_P (Skin permeation)	BBB permeant	CY1A2 inhibitor	GI absorption	p-gp substrate		
**4a**	-5.38 cm/s	Yes	yes	high	No	0 alert	3.44
**4b**	-5.44 cm/s	Yes	No	high	Yes	0 alert	3.57
**4c**	-6.01 cm/s	No	Yes	high	No	0 alert	3.57
**4d**	-4.81 cm/s	Yes	No	high	No	0 alert	3.70
**4e**	-5.75 cm/s	Yes	No	high	No	0 alert	3.63
**4f**	-4.47 cm/s	Yes	Yes	high	No	0 alert	3.54
**4g**	-4.46 cm/s	Yes	Yes	high	No	0 alert	3.57

Gastrointestinal absorption, GI absorption; blood-brain barrier, BBB; pan-assay interference structure, PAINS.
